# Iron Overload Mediates the Differential Cell Fate of Astrocytes from Neurons and Its Regulatory Mechanisms in Ischemic Stroke

**DOI:** 10.1002/advs.202507384

**Published:** 2025-11-06

**Authors:** Yi Guo, Yue Wang, Yong Ni, Bin Bo, Jinzhi He, Yongming Zhu, Aiping Qin, Xianyong Zhou, Huaping Du, Yuan Liu, Tianyao Wang, Yudu Li, Yibo Zhao, Zengai Chen, Zhipei Liang, Yao Li, Yuan Xu, Huiling Zhang

**Affiliations:** ^1^ Jiangsu Key Laboratory of Drug Discovery and Translational Research for Brain Diseases, Suzhou International Joint Laboratory for Diagnosis and Treatment of Brain Diseases Department of Pharmacology, College of Pharmaceutical Sciences Soochow University Suzhou Jiangsu 215123 China; ^2^ Department of Neurology Suzhou Ninth People's Hospital Suzhou Ninth Hospital Affiliated to Soochow University Soochow University Suzhou 215200 China; ^3^ Department of Pain and Suzhou Clinical Research Center of Neurological Disease the Second Affiliated Hospital of Soochow University Suzhou Jiangsu 215000 China; ^4^ National Engineering Research Center of Advanced Magnetic Resonance Technologies for Diagnosis and Therapy (NERC‐AMRT) School of Biomedical Engineering Shanghai Jiao Tong University Shanghai 200000 China; ^5^ Department of Pharmacy Jiangsu Health Vocational College Nanjing Jiangsu 210000 China; ^6^ Radiology Department Renji Hospital Shanghai Jiao Tong University of Medicine Shanghai 200000 China; ^7^ Beckman Institute for Advanced Science and Technology University of Illinois at Urbana‐Champaign Urbana IL 61801 USA; ^8^ Department of Bioengineering University of Illinois at Urbana‐Champaign Urbana IL 61801 USA; ^9^ National Center for Supercomputing Applications University of Illinois at Urbana‐Champaign Urbana IL 61801 USA; ^10^ Department of Electrical and Computer Engineering University of Illinois at Urbana‐Champaign Urbana IL 61801 USA; ^11^ Institute of Medical Robotics Shanghai Jiao Tong University Shanghai 200000 China

**Keywords:** astrocytes, iron overload, ischemic stroke, neuron, TfR1 palmitoylation

## Abstract

Iron accumulation and ferroptosis occur in the brain following ischemic stroke. However, the relationship between iron overload and cell type‐specific fates remains largely unclear. Here, iron deposition and neuronal loss are reported within the perilesional cortex of three patients with ischemic stroke at both acute and subacute stages. It is identified that ischemia/reperfusion‐induced iron overload triggers ferroptosis predominantly in neurons and to a lesser extent in astrocytes, whereas most astrocytes undergo reactive proliferation. Mechanistically, the reduced or elevated Nrf2/GPX4 and SLC7A11 levels in neurons or astrocytes, respectively, account for these distinct iron overload‐induced cellular fates. Moreover, iron overload promotes astrogliosis by enhancing the transcriptional activities of several proliferation‐related genes. Using mice with partial knockout of the transferrin receptor 1 (TfR1) gene *Tfrc*, astrocyte‐specific *Tfrc* knockdown, and conditional astrocytic *Cpt1a* partial knockout (to induce fatty acid metabolism disorders), it is revealed that increased TfR1 palmitoylation and clathrin‐mediated endocytosis drive astrocytic iron overload. Notably, ischemia/reperfusion‐induced elevation of palmitic acid is associated with enhanced TfR1 palmitoylation. Treatment with antioxidants or iron chelators mitigates ischemic brain injury. Together, these findings provide a comprehensive framework linking ischemia/reperfusion‐induced iron overload to cell type‐specific fates. TfR1 palmitoylation emerges as a potential target for ischemic stroke therapy.

## Graphical abstract

Upon ischemia/reperfusion‐induced iron overload condition, neurons and astrocytes exhibit similar oxidative stress changes but opposite alterations in endogenous antioxidant defense mechanisms, leading to divergent ferroptotic outcomes. The iron deposition and neuronal loss are increased within the perilesional cortex of stroke patients. Mechanistically, increased TfR1 palmitoylation modified by palmitic acid promotes TfR1‐mediated iron endocytosis and iron overload in astrocytes.

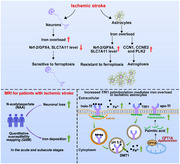

## 1 Introduction

Stroke is the second leading cause of death in the world, including ischemic stroke and hemorrhagic stroke. Ischemic stroke accounts for the major type of all strokes.^[^ ^1^ ^]^ Currently, although some progress has been achieved in helping patients recover from ischemic stroke with the advancement in recanalization therapy by using both thrombolytic agents such as alteplase (tPA) and tenecteplase (TNK‐tPA) and endovascular thrombectomy, there is still an urgent need for discovering new drug targets and brain cytoprotective agents in order to protect the brain from injury prior to and during or after recanalization, to extend the therapeutic time window for intervention and further to improve functional outcome.^[^ ^2^, ^3^ ^]^

Neuronal cell death and glial activation, and glial scar formation are two major pathohistological features of ischemic stroke. Following ischemic stroke, cells in the ischemic core rapidly die, including neurons and astrocytes, however, in the peri‐infarct area, neurons suffer severe damage and a substantial portion of them die, whereas astrocytes are transformed into reactive astrocytes showing hypertrophy and proliferation, finally forming a glial scar in the chronic phase of ischemic stroke.^[^ ^4^, ^5^, ^6^ ^]^ Reactive astrocytes, the major cellular component of the glial scar, are associated with abnormal neuronal activity and neuronal cell death in ischemic stroke. However, the pathophysiological mechanisms for differential fates between neurons and astrocytes in the peri‐infarct area are still largely unclear until now.

Ferroptosis is an iron‐dependent programmed cell death, characterized by the imbalance between oxidative stress and anti‐oxidative defense and the accumulation of lipid peroxides.^[^ ^7^, ^8^, ^9^ ^]^ Cystine/glutamate antiporter (System Xc^−^)‐glutathione (GSH)‐glutathione peroxidase 4 (GPX4) is the major classical ferroptosis defense system, which plays a protective role against ferroptosis by alleviating lipid peroxidation.^[^ ^10^ ^]^ GPX4 is the most crucial enzyme for attenuating lipid hydroperoxide accumulation in most cells via reducing potentially toxic lipid hydroperoxides to less dangerous lipid alcohols.^[^ ^11^ ^]^ The cell surface System Xc^−^ cystine/glutamate antiporter imports cystine in exchange for glutamate of which solute carrier family 7 member 11 (SLC7A11) is a key functional subunit. In the cytosol, cystine is rapidly reduced to cysteine. Cysteine is used to synthesize GSH. GSH is the cofactor for GPX4. It has been demonstrated that ischemic stroke induces significant down‐regulation of System Xc^−^‐GSH‐GPX4 levels in neurons, leading to neuronal ferroptosis.^[^ ^12^ ^]^ Nuclear factor erythroid 2‐related factor 2 (Nrf‐2) is a transcription factor that regulates the expression of its target genes associated with endogenous antioxidant defense systems, including GPX4 and SLC7A11, preventing cells from excessive oxidative stress response and ferroptosis.^[^ ^13^, ^14^, ^15^ ^]^ Therefore, we raised the question of whether the different cell fates between neurons and astrocytes are due to their different response to endogenous antioxidant defense systems, Nrf‐2‐mediated *GPX4* and *SLC7A11* gene expression, upon ischemic stroke insult.

Currently, iron overload is considered a crucial factor leading to ferroptosis in ischemic stroke.^[^ ^16^, ^17^ ^]^ After ischemic stroke, the impaired blood brain barrier allows large amounts of iron enter the brain parenchyma, leading to neuronal iron overload and ferroptosis.^[^ ^18^ ^]^ Iron chelators such as deferoxamine (DFO) can prevent ischemic stroke‐induced ferroptosis.^[^ ^16^ ^]^ Iron overload directly promotes reactive oxygen species (ROS) generation via the Fenton reaction, and the polyunsaturated fatty acids (PUFAs) are oxidized by intracellular ROS, resulting in cell membrane rupture.^[^ ^19^, ^20^ ^]^

In the central nervous system, astrocytes play an important role in the regulation of brain iron homeostasis, and they have twice as much iron as neurons.^[^ ^21^, ^22^ ^]^ Iron overload in astrocytes has been found in several neurological disorders, including Alzheimer's disease (AD), Parkinson's disease (PD), and multiple sclerosis (MS).^[^ ^23^ ^]^ In addition, hypoxia preconditioning induces astrocytes to uptake extracellular iron, leading to a progressive enhancement in cellular iron content.^[^ ^24^ ^]^ However, the relationship between iron overload and astrocytic ferroptosis and reactive proliferation, and glial scar formation is still largely unknown.

Iron overload results from the imbalance of cellular iron homeostasis. The intracellular labile iron pool is modulated by the uptake, export, storage, and utilization of iron. Cellular iron uptake is primarily regulated by the transferrin/transferrin receptor (Tf‐TfR) system, which has been shown to play a role in ferroptosis.^[^ ^25^ ^]^ Transferrin receptor 1 (TfR1) is widely expressed in human cells, and it is composed of two homodimer subunits linked by disulfide bonds. Extracellular free Fe^3+^ binds to Tf, leading to Tf conformation changes and its combination with TfR1 at the cell membrane.^[^ ^26^ ^]^ The Fe^3+^‐Tf‐TfR1 complex forms vesicles through clathrin‐mediated endocytosis, and the Fe^3+^ is reduced to Fe^2+^ by STEAP3 (STEAP family member 3) under the acidic intracellular environment, and divalent metal transporter 1 (DMT1) mediates the release of Fe^2+^ from the endosome to the labile iron pool in the cytoplasm. Meanwhile, the Tf‐TfR1 complex translocates to the cell surface from the Golgi apparatus, and then the Tf dissociates from TfR1 to complete Tf‐TfR1 recycling.^[^ ^27^ ^]^ A large amount of extracellular iron is transported into the cytoplasm in case of the acceleration of Tf‐TfR1 recycle, leading to cellular iron overload.^[^ ^28^ ^]^ Palmitoylation is a post‐translational lipid modification of proteins in which a fatty acyl chain (a 16‐carbon palmitic acid) is attached to cysteine residues of protein. Palmitoylation is an important event that alters the localization, such as trafficking proteins to the cell membrane, stability, and function of target proteins. Dysregulation of palmitoylation has been associated with various diseases, including metabolic disorders, cancers, neurological diseases, and infections.^[^ ^29^ ^]^ Palmitoylation plays an important role in the localization of TfR1 to the cell membrane, regulating the recycling of Tf‐TfR1 and mediating iron uptake in cells. Some studies have reported that dysregulation of TfR1 palmitoylation affects Tf‐TfR1 recycle, leading to cellular iron overload in neurodegenerative disease.^[^ ^30^, ^31^ ^]^ However, how palmitoylation of TfR1 causes intracellular iron overload in reactive astrocytes of ischemic stroke is still unclear.

In this study, we identify that ischemic stroke‐induced iron overload mediates most neurons and fewer astrocytes ferroptosis, while most astrocytes experience reactive proliferation. In particular, we elucidate iron deposition and impaired neuronal function/neuronal loss within the perilesional cortex of three patients with ischemic stroke at both acute and subacute stages. The mechanisms of these different cell fates are associated with the decreased or increased Nrf‐2/GPX4 and SLC7A11 level induced by iron overload in neurons or astrocytes, respectively. By using mice with *Tfrc* heterozygote knockout, astrocyte‐specific conditional *Tfrc* knockdown or conditional astrocytic C*pt1a* partial knockout (to induce astrocytic fatty acid disorder), we uncover that after ischemic stroke, an increase in TfR1 palmitoylation and clathrin‐mediated TfR1 endocytosis results in elevated iron uptake and intracellular iron overload of astrocytes, and ischemic stroke‐induced increase in fatty acids, particularly, an elevated palmitic acid is associated with TfR1 palmitoylation enhancement. We demonstrate that antioxidant or iron chelators protect the brain against ischemic stroke injury.

## 2 Results

### 2.1 Astrocytes Have Different Cell Fates from Neurons Against Ischemic Stroke‐Induced Ferroptosis

**Figure 1:**
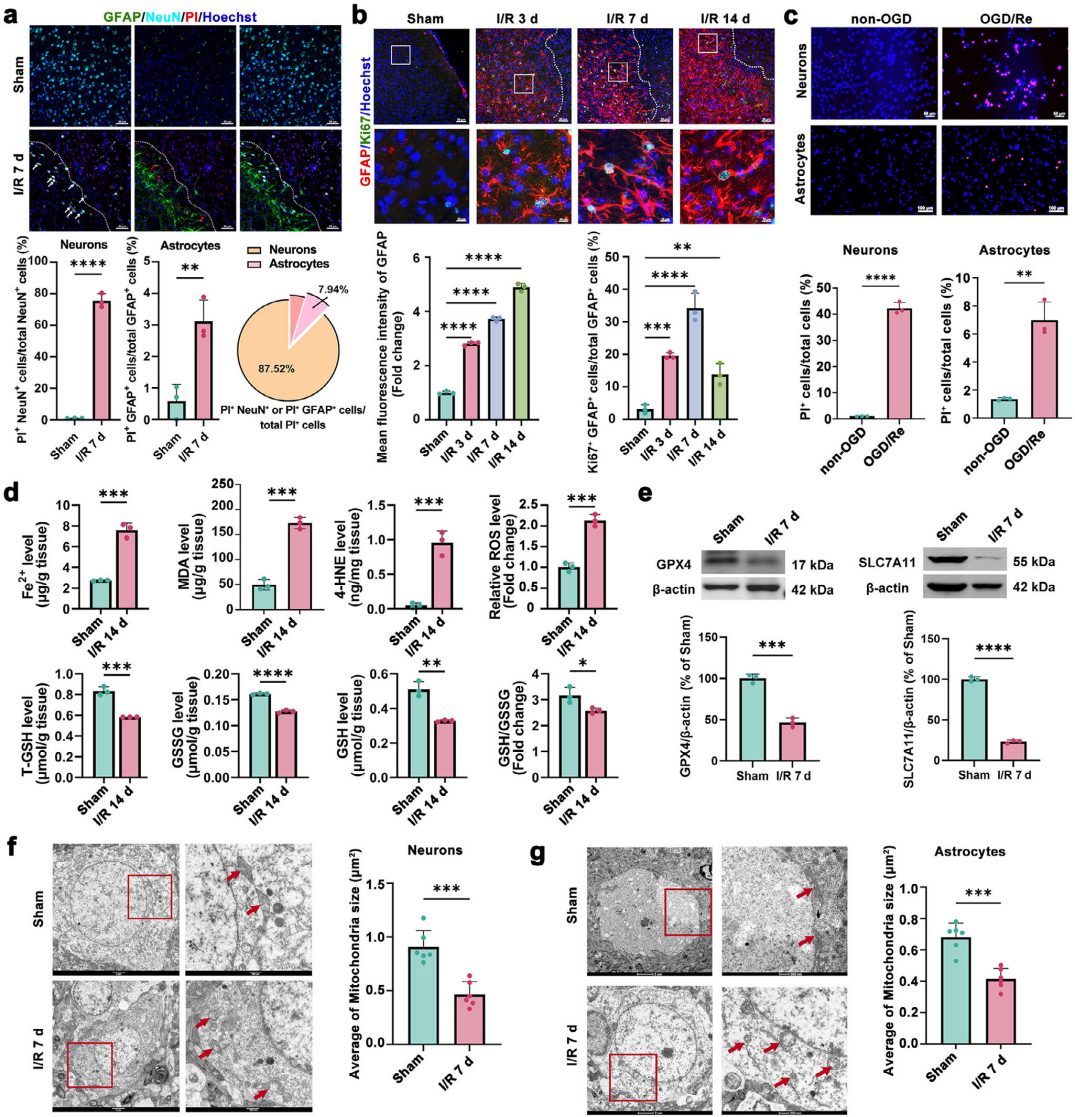
Ischemic stroke induces different cell fates of ferroptosis between neurons and astrocytes. a) PI staining (PI^+^, red) was used to detect the neuronal (NeuN, cyan) and astrocytic (GFAP, green) death in the peri‐infarct area 7 d after I/R. Cell nuclei (Hoechst): blue. White dotted lines, infarction border zones (IBZ). The ratio of PI^+^ NeuN^+^ cells/total NeuN^+^ cells, PI^+^ GFAP^+^ cells/total GFAP^+^ cells, and PI^+^ NeuN^+^ cells or PI^+^ GFAP^+^ cells/total PI^+^ cells were analyzed, respectively. Scale bar, 50 µm. b) Immunostaining of GFAP (red) and Ki67 (green), a proliferation marker in mice peri‐infarct area at d 3, d 7, and d 14 post‐I/R. Hoechst: blue. The GFAP fluorescence intensity and ratio of Ki67^+^ GFAP^+^ cells/total GFAP^+^ cells were analyzed. Scale bars, 50 or 10 µm. c) PI staining was used to detect cell death (PI^+^, red) in OGD 6 h‐Re 24 h‐treated HT22 cells or OGD 3 h‐Re 24 h‐treated HA cells. Hoechst: blue. The ratio of PI^+^ cells/total cells was analyzed. Scale bars, 50 or 100 µm. d) The levels of Fe^2+^, MDA, 4‐HNE, ROS, T‐GSH, GSSG, GSH, and GSH/GSSG in the peri‐infarct area of mice after I/R 14 d. e) Western blotting analysis of GPX4 and SLC7A11 in mice peri‐infarct area 7 d after I/R. Quantification expressed as a percentage of Sham group. f,g) Transmission electron microscopy was used to detect the mitochondrial morphology in neurons (f) and astrocytes (g) in rat peri‐infarct area 7 d after I/R. The average of mitochondria size was analyzed. Scale bars, 2 µm or 500 nm. Mean ± SD. n = 3 independent biological replicates (a–e), n = 6 independent biological replicates (f,g). Student's *t* test (a,c,d–g). One‐way ANOVA followed by a post hoc Tukey's test (b). ^*^ *p* < 0.05, *^*^ *p* < 0.01, **^*^ *p* < 0.001, ***^*^ *p* < 0.0001.

Ischemic stroke leads to a mass of brain cells damage and death. It has been demonstrated that in the peri‐infarct area, amounts of neurons die, whereas most astrocytes undergo proliferation and form glial scars. Our results also showed that in the peri‐infarct area (Figure S1a, Supporting Information) of cerebral cortex, most of the neurons (75.50 ± 4.48%) were propidium iodide‐positive (PI^+^), while only a few astrocytes (3.11 ± 0.67%) were PI^+^; and in PI^+^ cells, neurons accounted for 87.52 ± 1.04%, while astrocytes accounted for 7.94 ± 2.15% (**Figure** **1**a), on the contrary, astrocytes became reactive accompanied by increased levels of glial fibrillary acidic protein (GFAP), and an upregulated ratio of Ki67^+^ cells to GFAP^+^ cells that reached a peak at day 7 post‐ischemia/reperfusion (I/R), indicating that astrocytes present a state of proliferation in the peri‐infarct area (Figure 1b; Figure S1b, Supporting Information). Similarly, after mouse hippocampal neuronal cell line (HT22) exposed to oxygen‐glucose deprivation/re‐oxygenation (OGD/Re), the ratio of PI^+^ cells to total cells was 42.28 ± 2.23%, while in OGD/Re‐treated human astrocytes (HA), the ratio of PI^+^ cells to total cells was 6.87 ± 1.20% (Figure 1c), in contrast, the level of GFAP (Figure S1c, Supporting Information) and the ratio of Ki67^+^, a proliferation marker, or EdU^+^ cells to GFAP^+^ cells were significantly increased, respectively (Figure S1d, Supporting Information), indicating that most astrocytes become reactive and present proliferation upon OGD/Re treatment. These results suggest that astrocytes own different cell fates from neurons in the peri‐infarct area, presenting most neurons and a few astrocytes die, while most astrocytes experience proliferation.

Emerging evidence implicates ferroptosis as a mechanism of ischemic stroke‐induced injury.^[^ ^32^, ^33^ ^]^ Ferroptosis is characterized by iron accumulation and lipid peroxidation. Biochemically, this process is accompanied by increased oxidative stress, such as ROS and breakdown products of lipid peroxidation, such as malondialdehyde (MDA) and 4‐hydroxynonenal (4‐HNE), and depleted antioxidative defense, such as decreased GSH/GSSG ratio, GPX4, and SLC7A11.^[^ ^9^ ^]^ Therefore, these biomolecules usually act as markers to monitor ferroptosis, including Fe^2+^, MDA, 4‐HNE and ROS as well as GSH/GSSG ratio, GPX4, and SLC7A11. In this study, we found that in the peri‐infarct area of mice cerebral cortex 7 d post‐I/R, the pro‐ferroptotic markers, such as the levels of Fe^2+^, MDA, 4‐HNE, and ROS were significantly increased, while the anti‐ferroptotic markers, such as the ratio of GSH to GSSG, the levels of GPX4, and SLC7A11 were markedly decreased (Figure 1d,e). The transmission electron microscopy results revealed that both neurons and astrocytes presented morphology of ferroptosis, showing the shrunken mitochondria size, increased mitochondrial membrane density, and decreased cristae, indicating that ferroptosis occurs in both neurons and astrocytes in the peri‐infarct area of cerebral cortex of rats 7 d after ischemic stroke (Figure 1f,g). These results identify the presence of ferroptosis in both neurons and astrocytes in the peri‐infarct area. However, combined with the above cell death ratio for neurons and astrocytes, we can infer that neurons undergo ferroptosis, while only a small part of astrocytes experience ferroptosis, and most astrocytes undergo astrogliosis, indicating that they are resistant to ferroptosis.

### 2.2 Nrf‐2‐Mediated the Different Expression of GPX4 and SLCA711 in Neurons and Astrocytes Leads to Their Different Cell Fates after Ischemic Stroke

**Figure 2:**
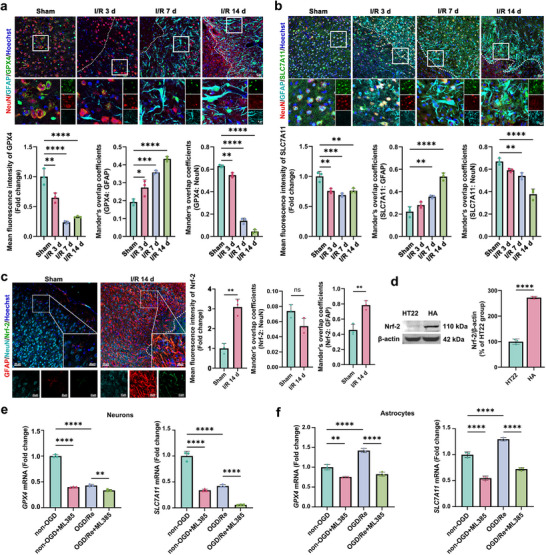
The levels of Nrf‐2, GPX4/SLC7A11 in neurons and astrocytes after ischemic stroke. a,b) Immunostaining showed the protein level of GPX4 (a) or SLC7A11 (b) in neurons and astrocytes at d 3, 7, and 14 after I/R. GFAP: cyan, NeuN: red, GPX4 or SLC7A11: green, and Hoechst: blue. White dotted lines, IBZ. The fluorescence intensity of GPX4 or SLC7A11 was analyzed, and the Mander's overlap coefficient was used to quantify colocalization analysis. Scale bars, 20 or 5 µm. c) Immunostaining showed the different protein level of Nrf‐2 in neurons and astrocytes. GFAP: red, NeuN: cyan, Nrf‐2: green, Hoechst: blue. The fluorescence intensity of Nrf‐2 was analyzed, and the Mander's overlap coefficient was used to quantify protein colocalization. Scale bars, 20 or 5 µm. d) Western blotting analysis of Nrf‐2 in HT22 and HA cells. Quantification expressed as a percentage of Nrf‐2 in HA cells to the HT22 group. e,f) RT‐qPCR analysis of the level of GPX4 and SLC7A11 mRNA in HT22 (e) and HA cells (f) with ML385 (a specific Nrf‐2 inhibitor) treatment. Mean ± SD. n = 3 independent biological replicates. One‐way ANOVA followed by a post hoc Tukey's test (a,b,e,f). Student's *t* test (c,d) ^*^ *p*<0.05, ^**^ *p* < 0.01, ^***^ *p* < 0.001, ^****^ *p* < 0.0001.

What accounts for the different fates of neurons and astrocytes against ischemic stroke‐induced ferroptosis? We first examined the pro‐ferroptotic factors in neurons and astrocytes after ischemic stroke, respectively. In OGD/Re‐treated HT22 cells or HA cells, the levels of the pro‐ferroptotic markers, such as the level of Fe^2+^, lipid peroxidation, and ROS were up‐regulated (Figure S2a–f, Supporting Information). The similar changes of Fe^2+^ and ROS were observed in primary cultured neurons and astrocytes (Figure S2g,h, Supporting Information). These results suggest that the pro‐ferroptotic markers are increased in both neurons and astrocytes after ischemic stroke. Next, we detected the levels of anti‐ferroptotic markers in neurons and astrocytes after ischemic stroke, respectively. GPX4 and SLC7A11 are two important anti‐ferroptotic biomolecules.^[^ ^11^ ^]^ We found that the protein levels of SLC7A11 and GPX4 were decreased in the peri‐infarct area, especially at d 7 and d 14 post‐I/R (**Figure** **2**a,b; Figure S3a,b, Supporting Information). However, to our surprise, the levels of GPX4 and SLC7A11 were different in neurons and astrocytes, showing that they were decreased in neurons, whereas they were increased in reactive astrocytes (Figure 2a,b; Figure S3a,b, Supporting Information). Consistent with the in vivo results, the levels of SLC7A11 and GPX4 were decreased in OGD/Re‐treated HT22 cells (Figure S3c, Supporting Information) and primary cultured neurons (Figure S3d, Supporting Information), whereas they were increased in OGD/Re‐treated HA cells (Figure S3e, Supporting Information) and primary cultured astrocytes (Figure S3f, Supporting Information) 12 h and/or 24 h post‐OGD/Re, when compared with the non‐OGD group, respectively. These results suggest that the levels of GPX4 and SLC7A11 are opposite, with down‐regulated in neurons and up‐regulated in astrocytes in the peri‐infarct area.

To prove that the reactive astrocyte in the peri‐infarct area can resist ferroptosis is likely due to their high expression of GPX4 and SLC7A11, two ferroptosis inducers, RAS‐selective lethal small molecule 3 (RSL3) and erastin were respectively used. RSL3 can trigger ferroptosis by inactivation of GPX4.^[^ ^34^ ^]^ Erastin can induce ferroptosis by inhibiting the system Xc^−^‐cystine‐glutamate antiporter at the plasma membrane.^[^ ^7^ ^]^ We first determined the optimal concentration of RSL3 or erastin to induce ferroptosis in HA cells. The results showed that RSL3 treatment with 0.1, 0.5, and 1 µm for 24 to 48 h could decrease cell viability in a dose‐dependent manner, and high concentrations of RSL3 at 10 or 100 µm produced cytotoxicity (Figure S4a, Supporting Information). In addition, RSL3 at 0.1 µm for 24 h could significantly reduce the levels of GPX4 and SLC7A11 (Figure S4b, Supporting Information). Similarly, treatment of erastin at 10 µm for 24 h also could decrease the cell viability (Figure S4a, Supporting Information) and downregulated the expressions of GPX4 and SLC7A11 (Figure S4b, Supporting Information). Thus, 0.1 µm RSL3 or 10 µm erastin was selected to suppress GPX4 and SLC7A11 in OGD/Re‐treated HA cells, respectively. Our results revealed that 0.1 µm RSL3 or 10 µm erastin treatment resulted in massive cell death and increased LDH leakage in OGD/Re‐treated HA cells (Figure S4c, Supporting Information), suggesting that inhibition of GPX4 or SLC7A11 abolishes the ability of astrocytes to resist ferroptosis, leading to astrocytes transforming from proliferation to cell death. These results reveal that up‐regulation of GPX4 and SLC7A11 is one of the critical mechanisms for reactive astrocytes to resist ischemic stroke‐induced ferroptosis.

Next, we observed the effect of ferroptotic astrocytes on normal astrocytes. HA cells were treated with or without RSL3 or erastin for 24 h, then replaced the medium with the normal culture medium and cultured for another 24 h. The cultured medium was harvested as conditioned medium (CM) and used to treat the normal cultured HA cells for 24 h, then the cell proliferation and the protein levels of GFAP, GPX4 and SLC7A11 were measured. The results showed that the RSL3 or erastin‐treated CM could increase the ratio of Ki67^+^ cells to GFAP^+^ cells (Figure S4d, Supporting Information) and the protein levels of GFAP, GPX4, and SLC7A11, compared to the control CM (Figure S4e,f, Supporting Information). These results suggest that ferroptotic astrocytes can induce astrocytes to become reactive astrocytes, thus we infer that the ferroptotic astrocytes in the peri‐infarct area may promote adjacent astrocytes to transform into reactive astrocytes.

Transcription within the nucleus is a crucial activity that contributes importantly to the modulation of ferroptosis sensitivity. Transcription factor Nrf‐2 regulates the expression of GPX4 and SLC7A11genes that govern redox homeostasis and ferroptosis sensitivity.^[^ ^14^, ^15^ ^]^ We found that in the mouse cerebral cortex, the expression of Nrf‐2 in neurons is extremely low under both non‐ischemia and ischemia conditions; in contrast, it has a certain expression in astrocytes of sham mice, and its expression was significantly increased in reactive astrocytes in the peri‐infarct area of mice 14 d post‐I/R (Figure 2c). Consistent with the in vivo results, the protein levels of Nrf‐2 in HT22 cells were significantly lower than those in HA cells (Figure 2d), upon OGD/Re exposure, the expression of Nrf‐2 was decreased in HT22 cells and primary cultured neurons, while it was increased in HA cells and primary cultured astrocytes (Figure S5a,b, Supporting Information), compared with that in the non‐OGD group, respectively. We considered that the different expression of Nrf‐2 in neurons and astrocytes may be responsible for the different expression of GPX4 and SLC7A11 in ischemic neurons and astrocytes. To verify this, ML385, a specific Nrf‐2 inhibitor, was used, which interacts with Nrf‐2 and affects its DNA binding activity, finally reducing the transcription of Nrf‐2 downstream target genes. It has also been reported that ML385 not only affects the activity of Nrf‐2, but also reduces the protein level of Nrf‐2.^[^ ^35^ ^]^ Immunofluorescence results showed that ML385 could significantly reduce the protein level of Nrf‐2 and its nuclear translocation in neurons and astrocytes under both non‐OGD and OGD/Re conditions (Figure S5c,d, Supporting Information). ML385 significantly decreased the mRNA expression of *GPX4* and *SLC7A11* (Figure 2e,f) and increased the number of PI^+^ cells in neurons and astrocytes after OGD/Re (Figure S5e, Supporting Information). These results suggest that low expression of Nrf‐2 in neurons and its decrease after ischemic stroke are associated with the reduction of the expressions of GPX4 and SLC7A11, resulting in neuronal ferroptosis; in contrast, higher expression of Nrf‐2 in astrocytes and its increase after ischemic stroke promote the expressions of GPX4 and SLC7A11, thus enhancing astrocytes resistance to ferroptosis, leading to astrogliosis. These results suggest that the different patterns of Nrf‐2‐mediated GPX4 and SLC7A11 expressions in neurons and astrocytes may cause differential cell fates for them upon ischemia/reperfusion. The decreased Nrf‐2 and Nrf‐2‐mediated GPX4 and SLC7A11 levels result in neuronal ferroptosis, while the increased Nrf‐2 and Nrf‐2‐mediated GPX4 and SLC7A11 levels lead to astrocytic resistance to ferroptosis, on the contrary, they exhibit abnormal proliferation and astrogliosis.

### 2.3 Iron Overload Mediates the Different Expressions of Nrf‐2/GPX4 and SLC7A11 in Neurons and Astrocytes, Leading to Their Different Cell Fates

Iron overload is a key factor in ferroptosis and can sensitize numerous cell types to ferroptosis. Following ischemic stroke, the impaired blood brain barrier allows large amounts of iron to enter the brain parenchyma, leading to neuronal iron overload and ferroptosis.^[^ ^18^ ^]^ Iron chelators such as DFO can prevent ischemic stroke‐induced ferroptosis.^[^ ^16^ ^]^

**Figure 3:**
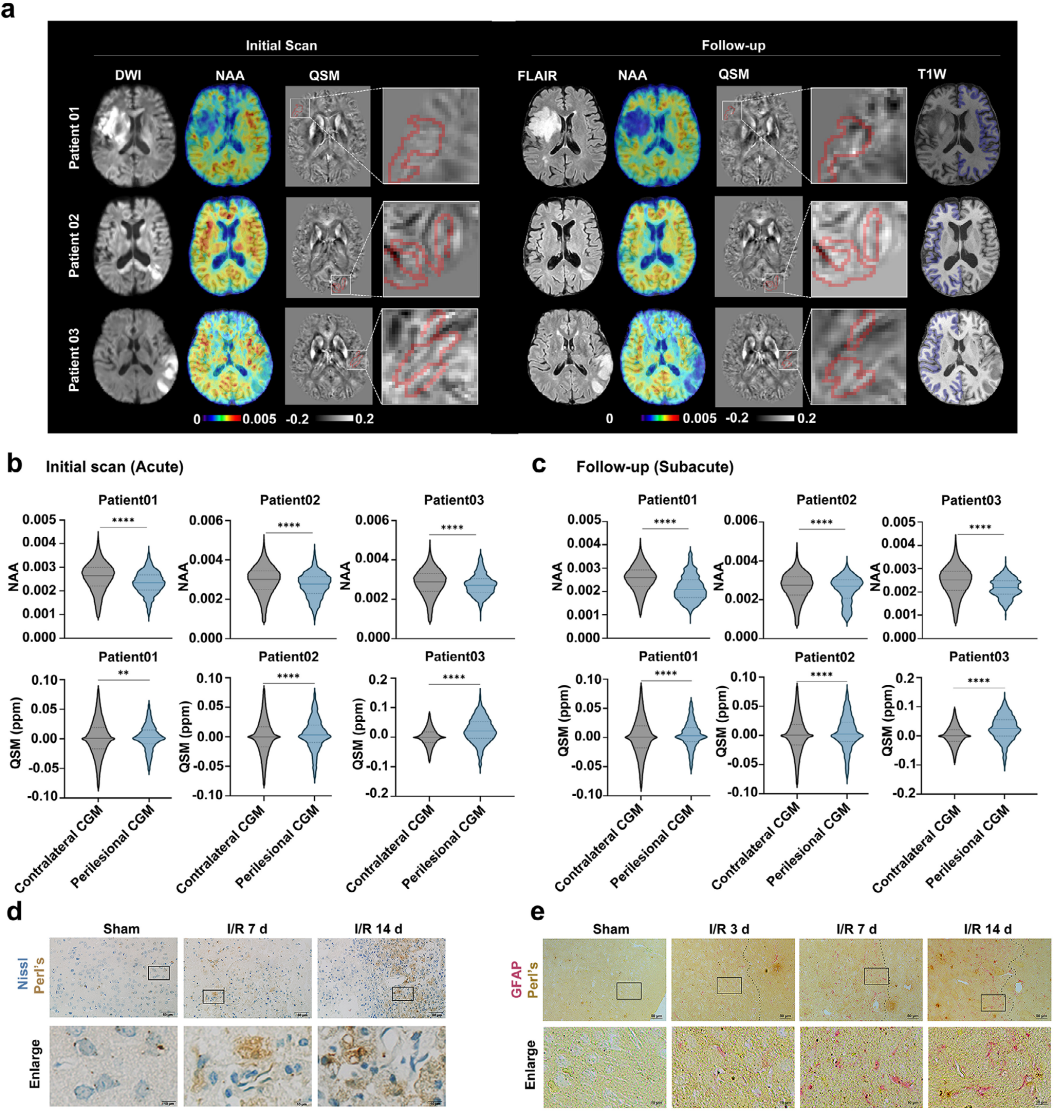
Iron deposits in the cerebral cortex in both ischemic stroke patients and mice. a) Multimodal brain images obtained from three ischemic stroke patients at both acute and subacute stages following ischemic stroke. NAA, a marker of neuronal integrity, and QSM images for iron concentrations were acquired across both time points. DWI images in the acute stage highlighted the initial lesion, while FLAIR images in the subacute stage delineated the final infarct regions. Close‐up views of the perilesional QSM are presented, with white boxes emphasizing the elevated tissue susceptibility levels. Regions of interest are delineated as follows: the perilesional cortex is outlined in red, and the contralateral cortical gray matter is outlined in blue. b,c) Violin plots comparing NAA and tissue susceptibility levels between the contralateral cortical gray matter and the perilesional cortex during the acute (b) and subacute stages (c). The plots display medians (solid lines) along with the first and third quartiles (dashed lines). d) Nissl & Perl's staining was used to detect the neuronal iron deposition in the peri‐infarct area of the cerebral cortex in mice with I/R insult. Iron deposition was visualized as yellow, brown, or even black granular deposits. Scale bars, 50 or 10 µm. e) GFAP & Perl's staining was used to detect iron deposition in astrocytes of the peri‐infarct area of the cerebral cortex in mice with I/R insult. Alkaline phosphatase staining (red/pink) was used to visualize GFAP (astrocytes), and iron deposition was visualized as yellow, brown, or even black granular deposits. Scale bars, 50 or 10 µm. Student's *t* test (b,c). ^**^ *p* < 0.01, ^****^ *p* < 0.0001.

High‐resolution 3D maps of N‐acetylaspartate (NAA) and quantitative susceptibility mapping (QSM) were obtained from three patients with symptom onset ranging from 9.5 to 60.5 h of acute stage and followed by a second Magnetic Resonance Imaging (MRI) scan 5 to 6 days later (subacute stages), respectively. Corresponding DWI, FLAIR, and T1w images are shown in **Figure** **3**a. Across all three patients, significantly lower NAA levels and higher tissue susceptibility were observed in the perilesional cortex compared to the contralateral cortex at both stages (Figure 3b,c). These findings suggest increased iron deposition and impaired neuronal function/neuronal loss within the perilesional cortex of stroke patients. Nissl and Perl's double staining showed the obvious iron deposition in neurons in the peri‐infarct area of mice cerebral cortex at d 7 and d 14 after I/R injury (Figure 3d). Additionally, iron deposition was seen in astrocytes in the peri‐infarct area at d 3, d 7, and d 14 post‐I/R injury (Figure 3e).

**Figure 4:**
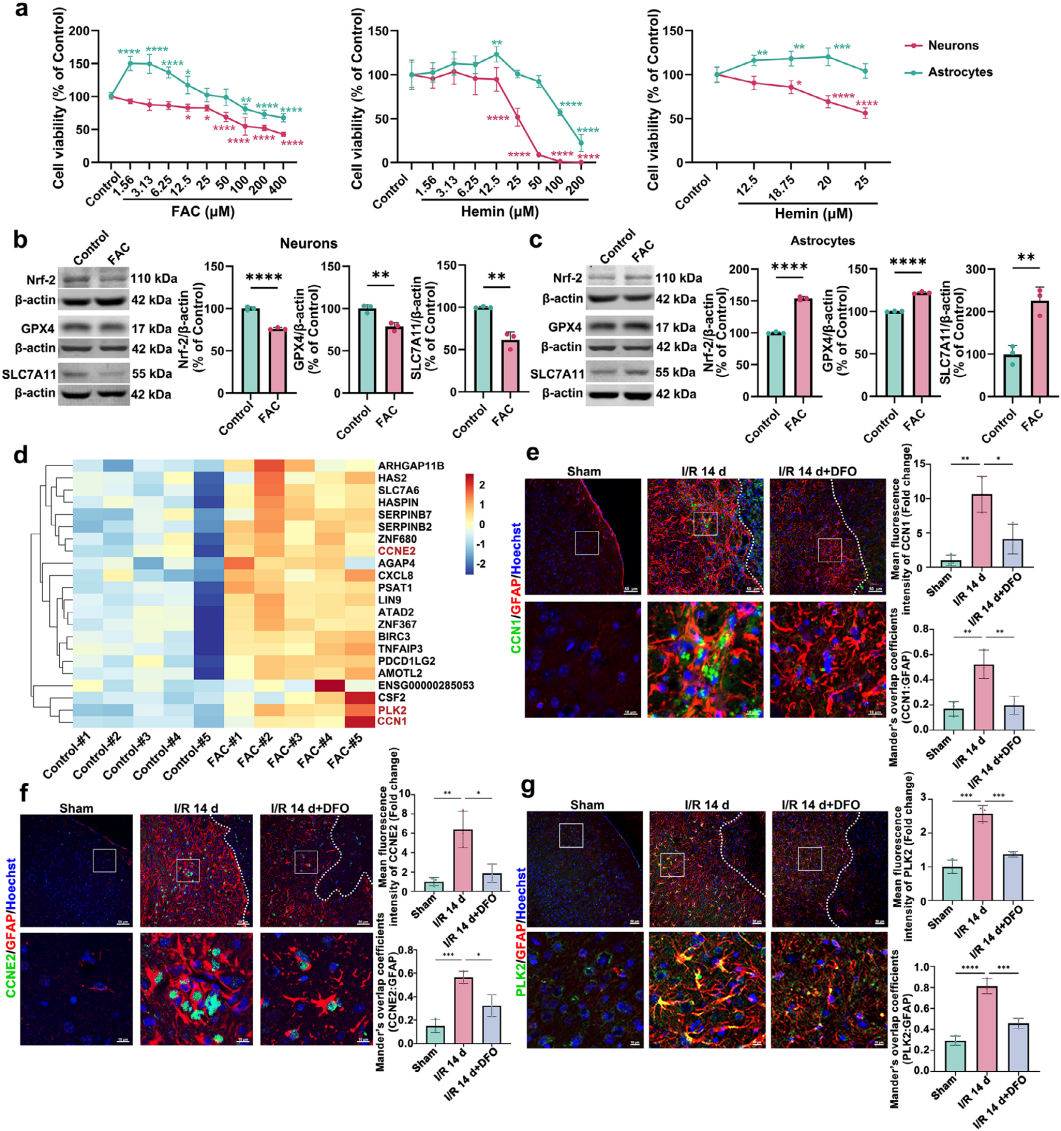
Iron overload mediates the different cell fates between neurons and astrocytes. a) HT22 cells or HA cells were cultured with different concentrations of FAC or hemin for 24 h, and the cell viability was measured with CCK‐8 assay. b,c) Western blotting analysis of Nrf‐2, GPX4 and SLC7A11 in HT22 or HA cells after 3.13 µm FAC treatment. d) HA cells were treated with 3.13 µm FAC for 24 h, and the RNA‐sequencing (RNA‐seq) analysis revealed the different proliferation‐related genes landscape between control and FAC‐treated HA cells. Heatmap of differentially expressed proliferation‐related genes between two groups of HA cells. e–g) Immunostaining images showed the changes of CCN1(e), CCNE2 (f) or PLK2 (g) in astrocytes of mice post‐I/R 14 d or ‐I/R 14 d+DFO. GFAP: red, CCN1, CCNE2 or PLK2: green, Hoechst: blue. The fluorescence intensity of CCN1, CCNE2, or PLK2 was analyzed, and the Mander's overlap coefficient was used to measure the colocalization of CCN1, CCNE2, or PLK2 with GFAP. Scale bars, 50 or 10 µm. Quantification expressed as a percentage of the control or Sham group. Mean ± SD. n = 6 independent biological replicates (a), n = 3 independent biological replicates (b–g). One‐way ANOVA followed by a post hoc Tukey's test (a,e–g). Student's *t* test (b, c). ^*^ *p*<0.05, ^**^ *p* < 0.01, ^***^ *p* < 0.001, ^****^ *p* < 0.0001.

To further demonstrate whether iron overload is one of the reasons for the different sensitivity to ferroptosis between neurons and astrocytes, we next applied two ferroptosis inducers to mediate ferroptosis by increasing intracellular iron, such as ferric ammonium citrate (FAC) and hemin. FAC is a physiological form of non‐transferrin‐bound iron that causes intracellular iron overload and leads to ferroptosis.^[^ ^31^ ^]^ Hemin, the prosthetic group of hemoglobin, leads to iron overload and ferroptosis.^[^ ^36^ ^]^ HT22 cells or HA cells were treated with FAC or hemin for 24 h at gradient concentrations. For neurons, small doses of FAC or hemin had no effect on cell viability, but when FAC concentration was higher than 3.13 µm or hemin concentration was higher than 18.75 µm, the cell viability was gradually decreased with the increase of FAC and hemin doses, showing a concentration‐dependent manner (**Figure** **4**a). For astrocytes, low doses of FAC (1.56–12.50 µm) or hemin (12.50–20 µm) increased the cell viability; when the FAC or hemin concentrations were higher than 100 µm, the cell viability started to decrease (Figure 4a). Further results showed that FAC at 3.13 µm induced ≈ 20% neuronal cell death evaluating with the rate of PI^+^ cells to total cells (Figure S6a, Supporting Information), and the same dose of 3.13 µm FAC only mediated ≈ 2–3% astrocytic cell death (Figure S6b, Supporting Information). In contrast, astrocytes exhibited proliferation with an increased ratio of Ki67^+^ cells to GFAP^+^ cells (Figure S6c, Supporting Information) and GFAP level (Figure S6d, Supporting Information) with FAC treatment. These results suggest that neurons and astrocytes have different sensitivity to iron overload, leading to their different fates facing iron overload in ischemic stroke. FAC at 3.13 µm induced the increased Fe^2+^ and ROS levels (Figure S6e, Supporting Information) in HT22 cells, and down‐regulated the levels of Nrf‐2, GPX4 and SLC7A11 in HT22 cells (Figure 4b). Differently, under the same FAC concentration, the Fe^2+^ and ROS levels were enhanced (Figure S6f, Supporting Information), and the levels of Nrf‐2, GPX4, and SLC7A11 were up‐regulated in HA cells (Figure 4c). These results suggest that iron overload mediates different expression patterns of Nrf‐2/GPX4 and SLC7A11 in neurons and astrocytes, respectively, causing their different cell fates, and that maintaining iron homeostasis is crucial to protect cells from ferroptosis in ischemic stroke.

We further investigated how iron overload induces the proliferation of astrocytes. We treated HA cells with 3.13 µm FAC to observe iron overload‐mediated changes in proliferation‐related genes via RNA sequencing (RNA‐seq) analysis. We found that several proliferation‐related genes in astrocytes were increased with FAC treatment (Figure 4d). Among these genes, three genes, including *CCN1* (*Cellular communication network factor 1*), *CCNE2* (*Cyclin E2*), and *PLK2* (*Polo like kinase 2*), were selected with the greatest inter‐group variation and the smallest intra‐group differences for further verification. The protein levels of CCN1, CCNE2, PLK2 were increased in FAC‐treated HA cells, compared with the control group (Figure S6g, Supporting Information). Further results demonstrated that the protein levels of CCN1, CCNE2, PLK2 in astrocytes were increased at d 14 post‐I/R, and DFO treatment could significantly reduce the levels of these three proteins (Figure 4e–g). Therefore, our data indicate that iron induces astrogliosis by promoting the transcriptional activities of several proliferation‐related genes.

### 2.4 Elevated TfR1 Expression and TfR1 Palmitoylation Contribute to Iron Overload after Ischemic Stroke

**Figure 5:**
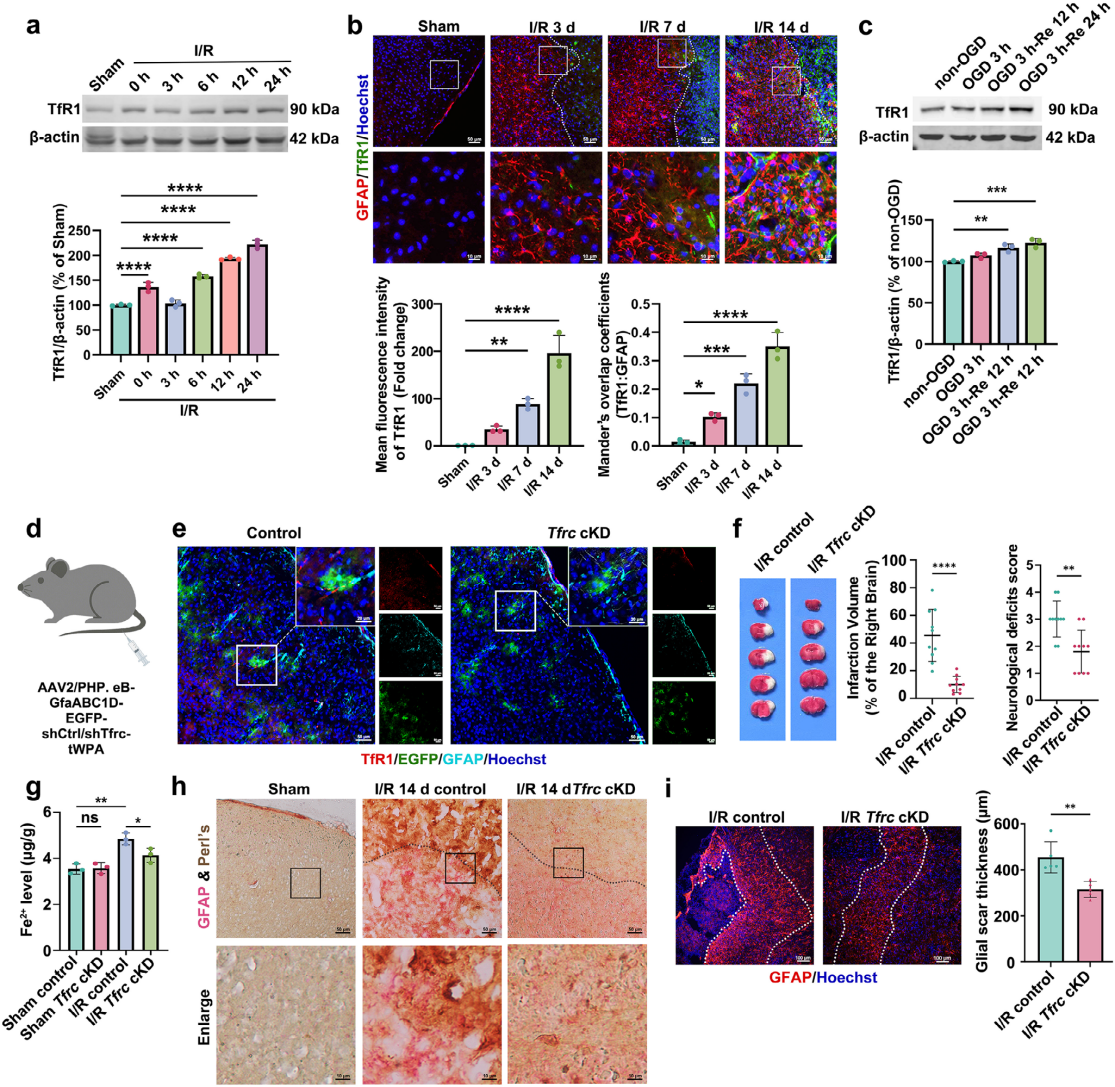
TfR1‐mediated iron overload contributes to ischemic stroke‐induced brain injury. a) Time course changes of TfR1 in peri‐infarct area of mice after I/R. Quantification expressed as a percentage of the Sham group. b) Immunostaining images showed the time course changes of TfR1 in astrocytes at d 3, 7, 14 post‐I/R. GFAP: red, TfR1: green, and Hoechst: blue. The fluorescence intensity of TfR1 was analyzed, and the Mander's overlap coefficient was used to measure the colocalization of TfR1 with GFAP. Scale bars, 50 or 10 µm. c) Time course changes of TfR1 in OGD/Re‐treated HA cells. Quantification expressed as a percentage of the non‐OGD group. d, e) Immunostaining showed astrocyte‐specific conditional *Tfrc* knockdown (*Tfrc* cKD) with astrocyte‐targeted AAVs (AAV2/PHP.eB‐GfaABC1D‐EGFP‐shTfrc‐tWPA) treatment. The EGFP labeling revealed the astrocytes that had been transfected with the AAVs. TfR1: red, EGFP: green, GFAP: cyan and Hoechst: blue. Scale bars, 50 or 10 µm. f) The cerebral infarction volume and neurobehavioral deficits scores in control and *Tfrc* cKD mice 24 h after I/R. g) The levels of Fe^2+^ in peri‐infarct area tissue at d 1 post‐I/R. h) GFAP & Perl's staining was used to detect iron deposition in astrocytes in control and *Tfrc* cKD mice after I/R 14 d. Alkaline phosphatase staining was used to visualize GFAP (astrocytes), and iron deposition was visualized as yellow, brown, or even black granular deposits. Scale bars, 50 or 10 µm. i) Immunostaining showed the glial scar thickness in control and *Tfrc* cKD mice 14 d after I/R. GFAP: red, Hoechst: blue. The glial scar thickness was analyzed. Scale bar, 20 µm. Mean ± SD, n = 3 independent biological replicates (a–c,g), n = 5 independent biological replicates (i), n = 10 independent biological replicates (f). One‐way ANOVA followed by a post hoc Tukey's test (a–c,g). Student's *t* test (f,i). ^*^ *p* < 0.05, ^**^ *p* < 0.01, ^***^ *p* < 0.001, ^****^ *p* < 0.0001.

Astrocytes are the major cells for ionized iron storage in the central nervous system, and they have twice as much iron content as neurons.^[^ ^22^, ^37^ ^]^ Astrocytic iron overload is caused by the imbalance of cellular iron homeostasis. The intracellular labile iron pool is modulated by the uptake, export, storage, and utilization of iron, and TfR1 is an important membrane receptor for cellular iron uptake.^[^ ^25^ ^]^ We next examined changes of TfR1 level in astrocytes and its relationship with iron overload. Our results showed that in the acute phase of ischemic stroke, the protein levels of TfR1 in the cerebral cortex were gradually increased from 6 to 24 h post‐I/R (**Figure** **5**a), accompanied by increased content of Fe^2+^ (Figure S7a, Supporting Information). During the subacute phase of ischemic stroke, the immunohistochemical results showed that the immunostaining of TfR1 in reactive astrocytes was increased from d 3 to d 14 post‐I/R and reached the peak at d 14 after I/R (Figure 5b; Figure S7b, Supporting Information), accompanied by a large amount of iron deposition (Figure 3e). Consistent with the in vivo results, the protein levels of TfR1 were increased in OGD/Re‐treated astrocytes (Figure 5c).

To further investigate the role of TfR1 in ischemic stroke, we constructed the *Tfrc* heterozygote knockout mice, which encodes TfR1 (Figure S7c, Supporting Information). Because the homozygotes of *Tfrc* knockout mice are embryonic lethal, thus the heterozygotes *Tfrc* knockout mice (*Tfrc^+/−^* mice) were used for subsequent experiments. The expression of TfR1 was decreased in cerebral cortex tissue of *Tfrc^+/−^* mice compared with that in *Wild type* (*WT*) mice (Figure S7d, Supporting Information), suggesting that the *Tfrc^+/−^* mice were successfully established. We found that compared with *WT* mice, *Tfrc^+/−^* mice presented reduced infarction volume, improved neurological deficits scores (Figure S7e, Supporting Information), and declined level of Fe^2+^ in peri‐infarct area 24 h after I/R (Figure S7f, Supporting Information); they also displayed decreased levels of brain tissue Fe^2+^ (Figure S7g, Supporting Information) and iron deposition in astrocytes (Figure S7h, Supporting Information) as well as glial scar thickness (Figure S7i, Supporting Information) in peri‐infarct area at d 14 after I/R. We also established astrocyte‐specific conditional *Tfrc* knockdown (*Tfrc* cKD) mice via astrocyte‐targeted adeno‐associated viruses (AAVs, AAV2/PHP. eB‐GfaABC1D‐EGFP‐shCtrl/shTfrc‐tWPA) transfection injected intravenously (Figure 5d,e). We found that *Tfrc* cKD reduced infarction volume (Figure 5f), improved neurological deficits scores (Figure 5f), and declined levels of Fe^2+^ in peri‐infarct area 24 h after I/R (Figure 5g); it also inhibited iron deposition in astrocytes (Figure 5h) and glial scar thickness (Figure 5i) in peri‐infarct area at d 14 post‐I/R. These results suggest that the increased TfR1‐mediated iron overload in astrocytes contributes to ischemic stroke‐induced brain injury and glial scar formation.

**Figure 6:**
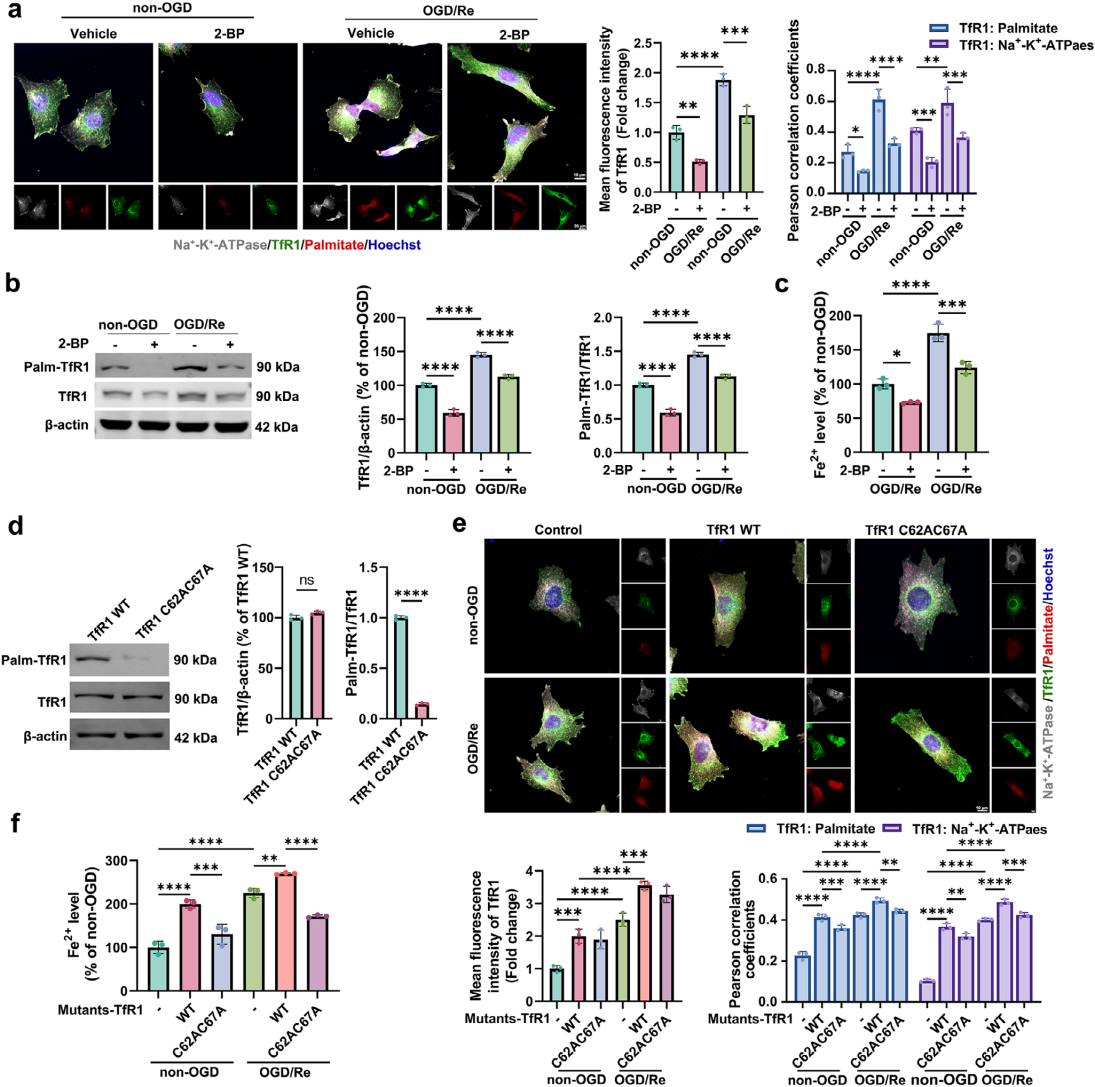
TfR1 palmitoylation contributes to iron overload in astrocytes. a–c) HA cells were cultured with 10 µm 2‐BP during reoxygenation for 24 h. a) Click‐chemistry‐reaction (CCR)‐based method showed the protein level and palmitoylation of TfR1 on the membrane. Cell membrane (Na^+^‐K^+^‐ATPase): gray, palmitoylated proteins (palmitate): red, TfR1: green, and Hoechst: blue. The fluorescence intensity of TfR1 was analyzed, and the Pearson correlation coefficient was used to measure the colocalization of TfR1 with Na^+^‐K^+^‐ATPase or TfR1 with palmitate. Scale bar, 10 µm. b) CCR results showed the protein and palmitoylation of TfR1 level. Quantification expressed as a percentage of the non‐OGD group and the fold change of palmitoylated‐TfR1 (palm‐TfR1) /total TfR1. c) The levels of Fe^2+^. d–f) The TfR1 WT and TfR1 C62AC67A plasmids were transfected into HA cells, and the TfR1 WT and TfR1 C62AC67A HA cells were established. d) CCR results showed the protein and palmitoylation of TfR1 level. Quantification expressed as a percentage of the TfR1 WT group and the fold change of palm‐TfR1/total TfR1. e) CCR results showed the protein and palmitoylation of TfR1 level on the membrane. Na^+^‐K^+^‐ATPase: gray, palmitate: red, TfR1: green, and Hoechst: blue. The fluorescence intensity of TfR1 was analyzed, and the Pearson correlation coefficient was used to measure the colocalization of TfR1 with Na^+^‐K^+^‐ATPase or TfR1 with palmitate. Scale bar, 10 µm. f) The levels of Fe^2+^. Mean ± SD. n = 3 independent biological replicates. One‐way ANOVA followed by a post hoc Tukey's test (a–c,e,f). Student's *t* test (d). ^*^ *p* < 0.05, ^**^ *p* < 0.01, ^***^ *p* < 0.001, ^****^ *p* < 0.0001.

The iron uptake function of TfR1 is regulated by its palmitoylation modification. Palmitoylation plays an important role in the localization of TfR1 to the cell membrane, regulating TfR1 function and mediating iron uptake in cells. Some studies have reported that dysregulation of TfR1 palmitoylation affects the Tf‐TfR1 cycle, leading to cellular iron overload in neurodegenerative diseases.^[^ ^30^, ^31^ ^]^ However, how palmitoylation of TfR1 causes iron overload in reactive astrocytes induced by ischemic stroke is still unclear. Using a click‐chemistry‐reaction (CCR)‐based method, we found that both total TfR1 palmitoylation and TfR1 palmitoylation on the cell membrane were increased in the OGD/Re‐treated HA cells (**Figure** **6**a,b), and 2‐bromopalmitate (2‐BP) treatment, a general protein palmitoylation inhibitor, could significantly reduce the level of total TfR1 (Figure 6b), total TfR1 palmitoylation (Figure 6b) and the localization of palmitoylated TfR1 on the cell membrane (Figure 6a), as well as decrease the Fe^2+^ level (Figure 6c). In addition, 2‐BP administration significantly reduced the iron deposition in reactive astrocytes (Figure S7j, Supporting Information) and the glial scar thickness (Figure S7k, Supporting Information) in the peri‐infarct area 14 d after I/R.

Some studies have reported that the palmitoylation sites in human TfR1 are in Cys^62^ and Cys^67^,^[^ ^38^, ^39^ ^]^ thus we explored the effects of Cys^62^ and Cys^67^ mutation on OGD/Re‐induced TfR1 palmitoylation and the level of Fe^2+^, ROS, and lipid peroxide in astrocytes. The TfR1 WT and TfR1 C62AC67A plasmids were transfected into HA cells, and Western blotting results confirmed that the Flag‐tagged TfR1 WT and TfR1 C62AC67A had been successfully expressed (Figure S8a, Supporting Information). CCR results showed that in TfR1 WT HA cells, the level of TfR1 palmitoylation was elevated and translocated to the cell membrane both under non‐OGD and OGD/Re treatment, while in TfR1 C62AC67A HA cells, the level of TfR1 palmitoylation (Figure 6d) and palmitoylated TfR1 on the cell membrane were decreased (Figure 6e), meanwhile, the levels of Fe^2+^ (Figure 6f), ROS (Figure S8b, Supporting Information) and lipid peroxide (Figure S8c, Supporting Information) were also decreased. CCR results showed that FAC at 3.13 µm could significantly induce an increase in the total protein levels and palmitoylation of TfR1 on the HA cell membrane (Figure S8d, Supporting Information). These results indicate that ischemic stroke‐induced elevation in TfR1 and TfR1 palmitoylation contributes to iron overload in reactive astrocytes.

Under physiological conditions, the Tf‐bound iron through TfR1 (Tf‐TfR1 complex) is internalized into the cell by clathrin‐mediated endocytosis upon its binding at the cell surface. In this process, clathrin forms special structures called clathrin‐coated pits, which surround the Tf‐TfR1 complex and promote its endocytosis.^[^ ^40^ ^]^ However, the effect of TfR1 palmitoylation on clathrin‐mediated TfR1 endocytosis remains unclear. Next, we explored the effects of TfR1 palmitoylation on clathrin‐mediated endocytosis of TfR1. Immunofluorescence results showed that OGD/Re caused increased co‐localization of TfR1 and clathrin in HA cells (Figure S9a, Supporting Information), and 2‐BP treatment reduced the co‐localization of them (Figure S9b, Supporting Information), suggesting OGD/Re mediates an increase in endocytosis of TfR1, and this is associated with OGD/Re‐induced TfR1 palmitoylation. Similarly, compared with TfR1 WT HA cells, TfR1 C62AC67A HA cells displayed reduced co‐localization between TfR1 and clathrin (Figure S9c, Supporting Information), indicating TfR1 palmitoylation can initiate TfR1 endocytosis. Moreover, in the FAC‐treated HA cells, the co‐localization of TfR1 and clathrin was increased compared with that in the control group (Figure S9d, Supporting Information), suggesting that an increase in extracellular iron promotes TfR1 endocytosis. The above results indicate that after ischemic stroke, elevated TfR1, TfR1 palmitoylation, and clathrin‐mediated TfR1 endocytosis, as well as increased extracellular iron, orchestrate to cause excess uptake of extracellular iron and intracellular iron overload in astrocytes.

### 2.5 The Increase of Palmitic Acid Promotes the Palmitoylation of TfR1 in Astrocytes after Ischemic Stroke

**Figure 7:**
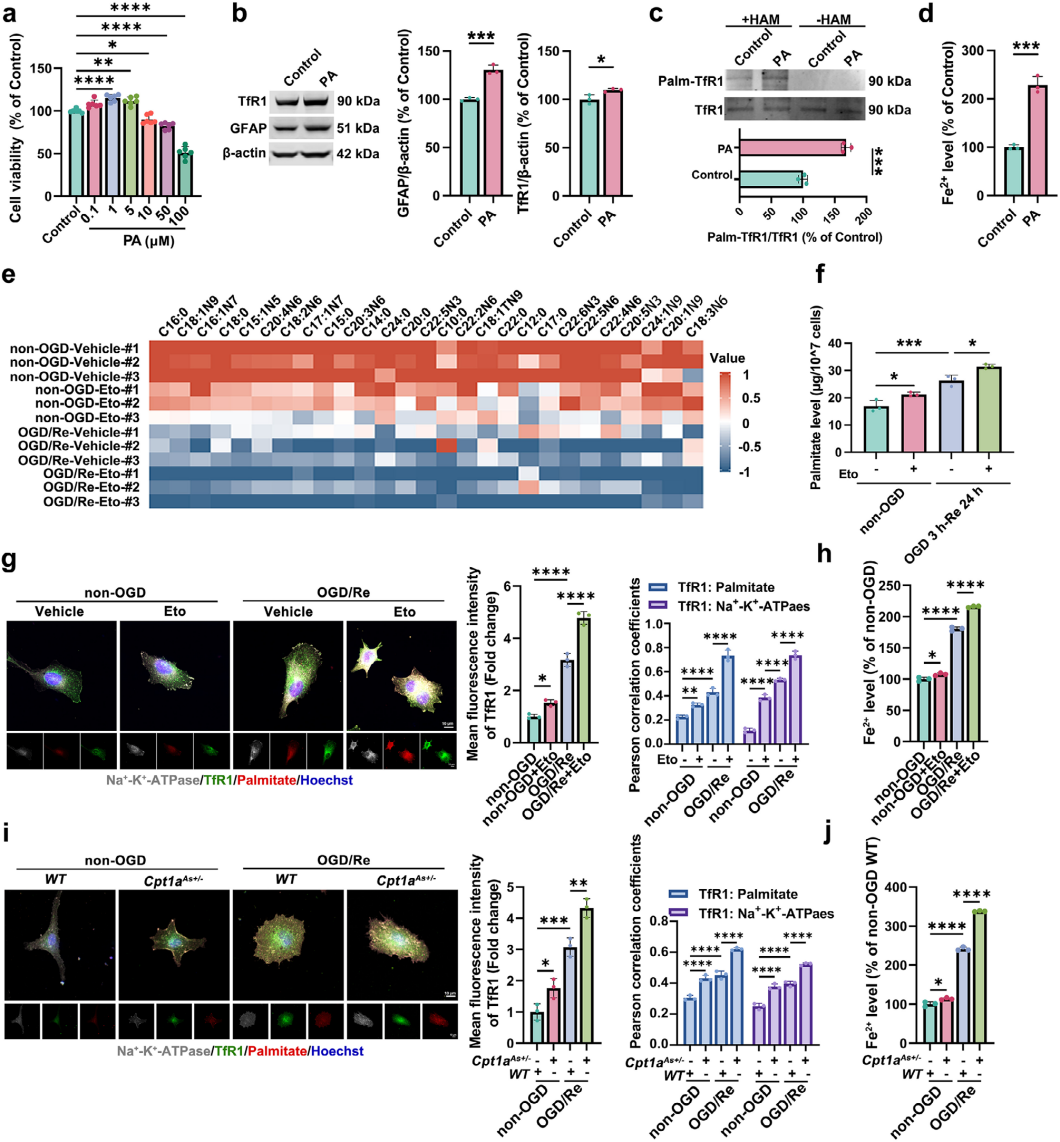
The increase of palmitic acid promotes the palmitoylation of TfR1 and iron overload in astrocytes. a) HA cells were cultured with different concentrations of palmitic acid (PA) for 24 h, and the cell viability was measured with CCK‐8 assay. (b–d) HA cells were cultured with 1 µm PA for 24 h. b) Western blotting analysis of TfR1 and GFAP. Quantification expressed as a percentage of the control group. c) TfR1 palmitoylation level with IP‐ABE assay. Quantification of palm‐TfR1/total TfR1. d) The levels of Fe^2+^. e–h) HA cells were cultured with 10 µm Eto during reoxygenation for 24 h. e) The heatmap showed the results of absolute quantification of various medium‐ and long‐chain fatty acids. f) The level of PA (per 10^7^ cells). g) CCR results showed the protein and palmitoylated TfR1 level on the membrane. Na^+^‐K^+^‐ATPase: gray, palmitate: red, TfR1: green, and Hoechst: blue. The fluorescence intensity of TfR1 was analyzed, and the Pearson correlation coefficient was used to measure the colocalization of TfR1 with Na^+^‐K^+^‐ATPase or TfR1 with palmitate. Scale bar, 10 µm. h) The levels of Fe^2+^ in HA cells. i,j) Primary cultured cerebral cortex astrocytes of *WT* and *Cpt1a^As+/−^* mice. i) CCR results showed the protein and palmitoylated TfR1 level on the membrane. Na^+^‐K^+^‐ATPase: gray, palmitate: red, TfR1: green, and Hoechst: blue. The fluorescence intensity of TfR1 was analyzed, and the Pearson correlation coefficient was used to measure the colocalization of TfR1 with Na^+^‐K^+^‐ATPase or TfR1 with palmitate. Scale bar, 10 µm. j) The levels of Fe^2+^ in primary cultured astrocytes. Mean ± SD. n = 6 independent biological replicates (a), n = 3 independent biological replicates (b‐j). One‐way ANOVA followed by a post hoc Tukey's test (a,f–j). Student's *t* test (b–d). ^*^ *p*< 0.05, ^**^ *p* < 0.01, ^***^ *p* < 0.001, ^****^ *p* < 0.0001.

A large amount of free fatty acids accumulates in the cerebrospinal fluid after ischemic stroke,^[^ ^41^, ^42^ ^]^ including palmitic acid (PA). PA is one of the most common fatty acids in the human body, and the increase of palmitic acid can provide a modifier for palmitoylation modification of proteins, such as TfR1.^[^ ^43^, ^44^ ^]^ Further, we investigated the effects of exogenous palmitic acid on the palmitoylation of TfR1 and the iron overload in astrocytes, as well as the proliferation of astrocytes. We found that PA at 1 and 5 µm could significantly increase cell viability, indicating that palmitic acid can promote the proliferation of HA cells (**Figure** **7**a). Treatment with 1 µm PA for 24 h could also promote the protein level of TfR1 (Figure 7b) and palmitoylated TfR1 (Figure 7c) and increase the intracellular Fe^2+^ level in HA cells (Figure 7d). These results suggest that PA promotes the palmitoylation of TfR1 and iron overload in astrocytes.

Fatty acid undergoes a series of enzymatic reactions and is oxidized within the mitochondria to produce energy. Carnitine palmitoyltransferase 1 A (CPT1A) is in the outer mitochondrial membrane by which long‐chain fatty acyl‐CoA converts to L‐palmitoylcarnitine, as a key step in the uptake of long‐chain fatty acids into mitochondria for β‐oxidative metabolism.^[^ ^45^ ^]^ We induced CPT1A dysfunction as a model of fatty acid metabolism disorder in which the palmitoylated modifier PA should be increased in astrocytes. We first observed the effects of etomoxir (Eto), an inhibitor of CPT1, on the level of palmitic acid. Medium‐ and long‐chain fatty acid measurements performed by Gas chromatography‐mass spectrometry (GC‐MS) showed that the levels of 27 medium‐ and long‐ chain fatty acids changed after OGD/Re or Eto treatment (Figure 7e; Data S1, Supporting Information); PA was increased in OGD/Re‐treated HA cells, and Eto treatment further increased the level of PA in those cells (Figure 7e,f). We next elucidated the effect of Eto‐induced CPT1A dysfunction on TfR1 palmitoylation. CCR analysis showed that the total protein and palmitoylated TfR1 level on the cell membrane were increased in the OGD/Re‐treated HA cells, and Eto treatment further enhanced OGD/Re‐mediated increase in the total protein level of TfR1, palmitoylated TfR1 and the localization of palmitoylated TfR1 on the cell membrane (Figure 7g; Figure S10a, Supporting Information) and Fe^2+^ level (Figure 7h). Next, we observed the effects of CPT1A dysfunction on iron overload in astrocytes using conditional astrocytic *Cpt1a* heterozvgous knockout mice (*Cpt1a^As+/−^* mice) (Figure S10b, Supporting Information). Western Blotting and immunohistochemistry results showed the expressions of CPT1A in cerebral cortex astrocytes of *Cpt1a^As+/−^* mice were decreased compared with *WT* mice (Figure S10c,d, Supporting Information). Similarly, after OGD/Re treatment, primary cultured conditional astrocytic *Cpt1a* heterozvgous knockout astrocytes (*Cpt1a^As+/−^* astrocytes) exhibited an increase in total TfR1 protein and palmitoylated TfR1 levels (Figure 7i; Figure S10e, Supporting Information), as well as Fe^2+^ level (Figure 7j), compared to OGD/Re‐treated *WT* astrocytes. As for in vivo, although there was no significant difference in Fe^2+^ level in peri‐infarct area of cerebral cortex between sham *Cpt1a^As+/−^* mice and sham *WT* mice (Figure S10f, Supporting Information), *Cpt1a^As+/−^* mice displayed increases in TfR1 staining (Figure s10 g, Supporting Information) and iron deposition in reactive astrocytes (Figure S10h, Supporting Information), and upregulated Fe^2+^ level (Figure S10i, Supporting Information) 14 d after I/R, compared to *WT* mice with I/R injury. In contrast, CPT1A overexpression in HA cells (Figure S10j, Supporting Information) reversed OGD/Re‐induced increase in total protein and palmitoylated TfR1 on the cell membrane (Figure S10k, Supporting Information), as well as Fe^2+^ level (Figure S10l, Supporting Information). These results indicate that CPT1A dysfunction enhances TfR1 palmitoylation, leading to iron overload in reactive astrocytes after ischemic stroke.

Next, endocytosis of TfR1 was detected by the co‐localization between TfR1 and clathrin. Immunofluorescence results showed that in OGD/Re‐treated HA cells, Eto treatment could promote the co‐localization between TfR1 and clathrin (Figure S11a, Supporting Information), while overexpression of CPT1A reduced the co‐localization between TfR1 and clathrin (Figure S11b, Supporting Information). These results indicated that CPT1A dysfunction promotes the palmitoylation of TfR1 and the clathrin‐mediated endocytosis of TfR1, accelerating the iron overload in relative astrocytes.

Next, we explored the effects of CPT1A dysfunction on glial scar formation and the change of ferroptotic regulators after ischemic stroke. Compared to *WT* mice with I/R insult, *Cpt1a^As+/−^* mice displayed the increases in GFAP protein levels and the ratio of Ki67^+^ GFAP^+^ cells to GFAP^+^ cells (Figure S12a, Supporting Information), as well as the glial scar thickness in the peri‐infarct area (Figure S12b, Supporting Information). Compared to those in *WT* mice, there were no significant changes in levels of GPX4, SLC7A11, TfR1 (Figure S12c, Supporting Information), as well as the content of 4‐HNE and MDA (Figure S12d, Supporting Information) in the cerebral cortex of *Cpt1a^As+/−^* mice. However, there was a significant increase in pro‐ferroptotic factors, such as ROS, MDA, and 4‐HNE, and a significant decrease in the GSH/GSSG in the peri‐infarct area in *Cpt1a^As+/−^* mice 14 d after I/R, which compared to *WT* mice (Figure S12e, Supporting Information). Interestingly, although the expressions of anti‐ferroptosis proteins GPX4 (Figure S12f, Supporting Information) and SLC7A11 (Figure S12g, Supporting Information) were decreased in the peri‐infarct area of *WT* mice, their expressions were increased in reactive astrocytes, and the expressions of GXP4 and SLC7A11 were further increased in reactive astrocytes of *Cpt1a^As+/−^* mice. Consistent with the in vivo results, Eto could promote the accumulation of ROS (Figure S13a, Supporting Information) and lipid peroxidation in reactive astrocytes (Figure S13b, Supporting Information), and further promote the expressions of GPX4 and SLC7A11 in OGD/Re‐treated HA cells (Figure S13c, Supporting Information). These results suggested that CPT1A dysfunction increases both pro‐ferroptotic factors levels and anti‐ferroptosis proteins GPX4 and SLC7A11 in reactive astrocytes, promoting the glial scar formation after ischemic stroke.

### 2.6 Antioxidant or Reducing Iron Overload Protects Brain against Ischemia/Reperfusion Injury

**Figure 8:**
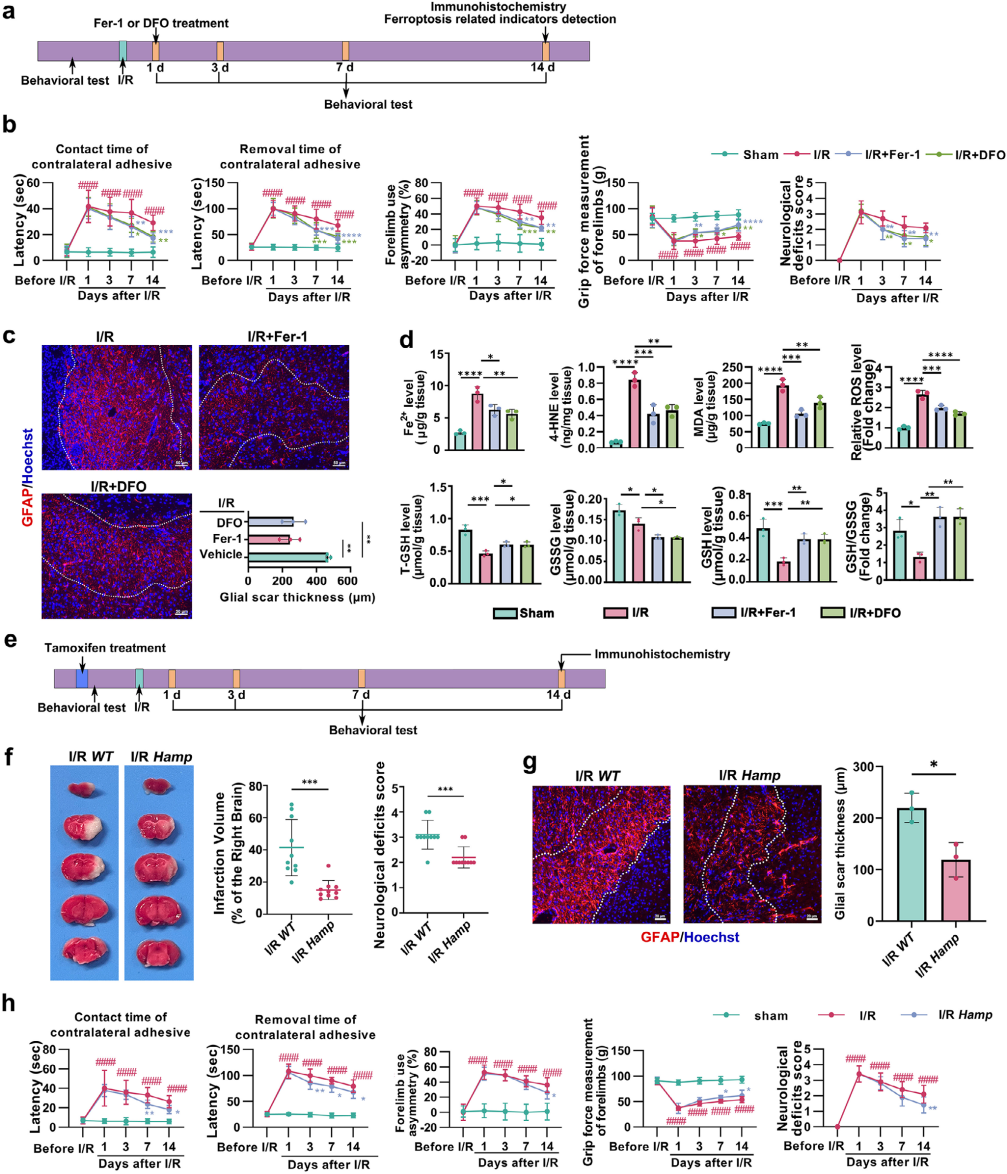
Antioxidant or reducing iron overload protects the brain against I/R injury. a–d) Fer‐1 (1 mg kg^−1^) or DFO (50 mg kg^−1^) were intravenously injected (i.v.) at d 1 post‐I/R, once every two days, until d 14 d post‐I/R. a) Experimental protocol. b) The neurological deficits were measured with four neurological deficit‐evaluating tests. ^####^ *p* < 0.0001 versus Sham group, ^*^ *p* < 0.05, ^**^ *p* < 0.01, ^***^ *p* < 0.001, ^****^ *p* < 0.0001 versus I/R group. c) Immunostaining showed the glial scar thickness in mice peri‐infarct area at d 14 post‐I/R. GFAP: red and Hoechst: blue. The glial scar thickness was analyzed. Scale bar, 50 µm. d) The levels of Fe^2+^, MDA, 4‐HNE, ROS, T‐GSH, GSSG, GSH, and GSH/GSSG in mice peri‐infarct area at d 14 post‐I/R. e–h) Tamoxifen (0.1 mL per mouse, 10 mg mL^−1^ dissolved in sunflower oil) were peritoneally injected (i.p.) for consecutive 5 d to induce the overexpression of *Hamp* in liver. e) Experimental protocol. f) The cerebral infarction volume and neurobehavioral deficits scores in *WT* and *Hamp* mice 1 d after I/R. g) Immunostaining showed the glial scar thickness in mice peri‐infarct area at d 14 post‐I/R. GFAP: red; Hoechst: blue. The glial scar thickness was analyzed. Scale bar, 20 µm. h) The neurological deficits were measured with four neurological deficit‐evaluating tests. ^####^ *p* < 0.0001 versus Sham group, ^*^ *p* < 0.05, ^**^ *p* < 0.01 versus I/R group. Mean ± SD, n = 10 independent biological replicates (b,f,h), n = 3 independent biological replicates (c,d,g). Multiple *t* tests with Mann‐Whitney test or two‐way ANOVA followed by a post hoc Tukey's test (b,h), and one‐way ANOVA followed by a post hoc Tukey's test (c,d), Student's *t* test (f,g). ^*^ *p*<0.05, ^**^ *p* < 0.01, ^***^ *p* < 0.001, ^****^ *p* < 0.0001.

Ferrostatin‐1 (Fer‐1), a synthetic lipophilic radical‐trapping antioxidant, is a potent and selective inhibitor of ferroptosis that can reduce cellular Fe^2+^, ROS, and lipid peroxidation, and regulate multiple processes of ferroptosis.^[^ ^46^ ^]^ DFO is an iron‐chelating agent used to treat iron overload.^[^ ^47^ ^]^ Fer‐1 at 1 mg kg^−1^ or DFO at 50 mg kg^−1^ could significantly improve neurological deficits scores, grip strength and forelimb use asymmetry, and reduce the time of contact and remove label in the adhesive removal tests (**Figure** **8**a,b), as well as reduce the thickness of glial scar (Figure 8c) and iron deposition in neurons and astrocytes in the peri‐infarct area (Figure S14a,b, Supporting Information), when mice were intravenously injected with them, respectively, every two days starting from d 1 to d 14 post‐I/R. Meanwhile, Fer‐1 and DFO could markedly reduce the levels of Fe^2+^, MDA, and 4‐HNE, and increase the level of GSH and the ratio of GSH/GSSG in the peri‐infarct area of cerebral cortex (Figure 8d). Consistent with the in vivo results, Fer‐1 and DFO increased the cell viability of HT22 cells and HA cells treated with OGD for 6 h (Figure S15, Supporting Information), decreased the number of PI^+^ cells in HT22 cells (Figure S16a, Supporting Information) or HA cells (Figure S16b, Supporting Information) with OGD/Re treatment, and significantly attenuated the levels of Fe^2+^, lipid peroxidation and ROS (Figure S16c–h, Supporting Information). In addition, Fer‐1 and DFO decreased the level of GFAP (Figure S16i, Supporting Information) and the ratio of Ki67^+^ cells to GFAP^+^ cells in OGD/Re‐treated HA cells (Figure S16j, Supporting Information), suggesting they can inhibit reactive astrogliosis. Interestingly, Fer‐1 and DFO could restore the decreased protein level of GPX4 and SLC7A11 in OGD/Re‐treated HT22 cells (Figure S17a, Supporting Information), but they had no significant effect on the levels of GPX4 and SLC7A11 in HA cells after OGD/Re (Figure S17b, Supporting Information). These results suggest that the antioxidant Fer‐1 and the iron chelator DFO can reduce the neuronal and astrocytic damage, inhibit glial scar formation, and promote neurological function recovery.

Hepcidin is a polypeptide hormone and secreted by the liver, which is encoded by the *Hepcidin Antimicrobial Peptide* (*Hamp*) *gene*. Hepcidin can inhibit the iron absorption of intestinal epithelium and the recycling of iron from aging red blood cells through macrophages, thus resulting in reducing the level of iron in blood.^[^ ^48^ ^]^ To further investigate the effects of alleviating iron overload on ischemic stroke, we generated a double transgenic mouse line to conditionally overexpress the liver *Hamp*, as a model of low iron content in peripheral blood circulation. In liver conditional *Hamp* overexpression mice (*Hamp* mice), expression of hepcidin is driven by a liver‐specific albumin promoter controlled by CRE‐ERT that only turns on in the presence of tamoxifen (Figure S18a, Supporting Information). It has been reported that these mice with low iron had no significant effect on body weight and body function.^[^ ^49^ ^]^ In the current study, the level of hepcidin in liver, serum, and intestine was increased, confirming that *Hamp* mice were successfully established (Figure s18b, Supporting Information). We observed that the Fe^2+^ level in the cerebral cortex was reduced in both sham and I/R *Hamp* mice (Figure S18c, Supporting Information). After ischemic stroke, *Hamp* mice exhibited reduced cerebral infarction volume (Figure 8e,f), decreased glial scar thickness (Figure 8g), improved neurological deficit scores, grip strength and forelimb use asymmetry, and reduced the time to contact and to remove the contralateral adhesive (Figure 8h), and downregulated astrocytic iron deposition (Figure S18d, Supporting Information) in the peri‐infarct area, compared to *WT* mice. The above results suggest that reducing iron overload in brain tissue can inhibit the glial scar formation and promote the neurological function recovery after ischemic stroke.

## 3 Discussion

The data presented in this study introduce a new concept to cell biology. Upon ischemia/reperfusion‐induced iron overload condition, neurons and astrocytes exhibit similar oxidative stress changes but opposite alterations in endogenous antioxidant defense systems, resulting in distinct ferroptotic susceptibilities: neurons are more prone to ferroptosis, whereas astrocytes are comparatively resistant. We further uncover that an increase in palmitoylation of TfR1 triggers clathrin‐mediated TfR1 endocytosis and uptake of extracellular iron, resulting in intracellular iron overload in astrocytes. Ischemia/reperfusion‐induced increase in fatty acid, particularly an elevated palmitic acid, is closely related to TfR1 palmitoylation enhancement. We demonstrate that application of anti‐oxidative agents or lowering the iron level in the brain can protect the brain against ischemic stroke‐induced damage.

Neuronal cell death and glial activation, and glial scar formation are two major pathohistological features of ischemic stroke. Consistently, in our studies, we first confirmed that in the peri‐infarct area, neurons and astrocytes have different cell fates: many neurons die, whereas only a few astrocytes are dead, and most astrocytes present proliferation, and they finally form glial scars.

It has been shown that ferroptosis contributes to ischemic stroke‐mediated brain damage. In agreement, in the peri‐infarct area 7 d or 14 d after ischemic/reperfusion, we identified increased oxidative stress biomarkers such as Fe^2+^, ROS, MDA, and 4‐HNE and decreased antioxidant defense factors including GSH/GSSG, GPX4, and SLC7A11, indicating ferroptosis occurs in ischemic brain tissues. Previous studies had already revealed that ferroptosis occurs in neurons under ischemic stroke conditions. However, whether astrocytes experience ferroptosis after ischemic stroke insults remains largely unknown. Unprecedentedly, although electron microscopy revealed morphological hallmarks of ferroptosis, such as mitochondrial shrinkage and loss or reduction of cristae in both neurons and astrocytes within the peri‐infarct cerebral cortex, and Perl's staining demonstrated substantial iron accumulation in both cell types, quantitative analysis of cell death revealed divergent ferroptotic responses. Following ischemia/reperfusion injury, neurons exhibited marked susceptibility to ferroptosis, whereas only a small fraction of astrocytes underwent ferroptotic death. In contrast, most astrocytes were resistant to ferroptosis, displaying reactive proliferation and contributing to glial scar formation.

Ferroptosis is characterized by the imbalance between oxidative stress and anti‐oxidative defense and the accumulation of lipid peroxides.^[^ ^7^, ^8^ ^]^ Precisely detecting pro‐ or anti‐ferroptosis factors might enable us to identify the reasons for this distinct ferroptosis profile between neurons and astrocytes. To our surprise, our further results indicated that upon ischemia/reperfusion insults, neurons and astrocytes exhibited similar increased changes in levels of Fe^2+^ and oxidative stress, including ROS and lipid peroxidation, but opposite alterations in endogenous antioxidant defense systems, showing a decrease in neurons or an increase in astrocytes in GPX4 and SLC7A11 level, respectively. Importantly, when we added two ferroptosis inducers in OGD/Re‐treated HA cells, RSL3 or erastin to inactivate GPX4 or to inhibit SLC7A11, respectively, astrocytes transformed from proliferation to cell death, indicating that OGD/Re‐mediated higher expression of GPX4 and SLC7A11 enables astrocytes to resistance to disease stress‐induced ferroptosis, on the contrary, they exhibit astrogliosis and glial scar formation. Consistent with our results, Savaskan et al. reported that GPX4 expression was low in astrocytes of normal brain tissue but was strongly upregulated in reactive astrocytes after electro‐coagulator‐induced brain injury.^[^ ^50^ ^]^ In addition, SLC7A11 expression is upregulated in astrocytes in the mouse spinal cord injury model.^[^ ^51^ ^]^ Furthermore, cancer cells exhibit the ability to evade ferroptosis by activation of antioxidant signaling pathways such as SLC7A11/GPX4 axis.^[^ ^52^ ^]^ A recent study conducted by Garcia‐Saez research group identified that iron‐dependent lipid peroxidation and ferroptotic death can spread to neighboring cells through their closely adjacent plasma membranes, thereby promoting the transmission of ferroptotic cell death with consequences for pathological tissue necrosis spread, using a novel optogenetic tool for light‐controlled induction of ferroptosis. They further found that transferring ferroptotic supernatants to healthy cells did not result in a death signal in the recipient cells.^[^ ^53^ ^]^ However, in our present study, we treated normal astrocytes with the conditioned medium derived from ferroptotic inducers RSL3‐ or erastin‐treated astrocytes, the results showed that these astrocytes displayed astrogliosis, accompanied by increased levels of GPX4 and SLC7A11. We speculate that the toxic substances released from ferroptotic astrocytes may spread to the relatively remote astrocytes, leading to their proliferation, and this effect is different from physical contacts demonstrated by Garcia‐Saez group.^[^ ^53^ ^]^ Our findings supplement Garcia‐Saez's discovery in ferroptosis.

Nrf‐2 is a well‐known transcription factor that plays a key role in anti‐oxidation, regulating the gene transcription of GPX4 and SLC7A11. Normally, the expression of Nrf‐2 is inhibited in neurons of animals, whereas its expression is abundant in astrocytes, suggesting that astrocytes may have a higher antioxidant potential than neurons.^[^ ^54^ ^]^ Our results showed that the content of Nrf‐2 was much lower in neurons than in astrocytes; after ischemic stroke, the expression of Nrf‐2 was decreased in neurons while it was significantly increased in astrocytes. ML385, a specific inhibitor of Nrf‐2, could lead to an increase in PI^+^ cells, accompanied by reduced mRNA levels of *GPX4* and *SLC711* in both neurons and astrocytes. These findings suggest that differential expression of Nrf2 between neurons and astrocytes underlies the distinct alterations in GPX4 and SLC7A11 observed after ischemic stroke. The higher Nrf2‐mediated upregulation of GPX4 and SLC7A11 in astrocytes may account for their relative resistance to ferroptosis compared with neurons.^[^ ^55^ ^]^

It has been documented that iron overload plays a crucial role in ischemic stroke‐induced ferroptosis.^[^ ^16^, ^17^ ^]^ After ischemic stroke, due to the loss of tight junctional integrity, the blood brain barrier is impaired, and Fe^3+^ in the blood is released into the brain parenchyma with the assistance of TF and TfR1 complex.^[^ ^18^ ^]^ Subsequently, Fe^3+^ is reduced to Fe^2+^, leading to the cytoplasm Fe^2+^ overload and ferroptosis via the Fenton reaction‐mediated ROS accumulation.^[^ ^19^ ^]^ Importantly, increased iron deposition and impaired neuronal function/neuronal loss within the perilesional cortex of three patients with ischemic stroke were identified in this study, at both acute and subacute stages. One of the creative findings in the current study is that ischemic stroke‐mediated iron overload causes the opposite alterations in endogenous antioxidant defense systems, Nrf‐2‐mediated GPX4 and SLC7A11 expression in neurons and astrocytes. We induced neuronal cell and astrocytes iron overload by supplementing FAC (Ammonium ferric citrate) or Hemin. We found a suitable range of FAC (3.13–12.5 µm) or hemin (18.75–20 µm) could decrease or increase the cell viability for HT22 cells or HA cells, respectively. In addition, FAC at 3.13 µm induced many HT22 cells death, whereas it mediated astrocytes proliferation, accompanied by a decrease or an increase expression of Nrf‐2/GPX4 and SLCA711 in neurons or in astrocytes, respectively, indicating that neurons are more vulnerable to ferroptosis compared with astrocytes. Although we cannot rule out that other causes may also contribute to different cell fates between neurons and astrocytes, iron overload‐mediated opposite expression patterns in Nrf‐2/GPX4 and SLC7A11 between them, at least, partially, is responsible for the distinct ferroptosis profile of these two cells under ischemia/reperfusion conditions. Importantly, we further identified that FAC‐induced iron overload caused astrogliosis by promoting the transcriptional expression of several proliferation‐related genes, including CCN1, CCNE2, PLK2, and DFO treatment could significantly reverse these changes, indicating that iron induces astrogliosis through triggering the transcriptional activities of these proliferation‐related genes in astrocytes.

Other forms of cell death may also be involved in the differential response between neurons and astrocytes under central nervous system disease, including ischemic stroke, although there is no direct evidence. For example, neuronal necroptosis occurs in ischemic stroke.^[^ ^56^ ^]^ On the other hand, our group demonstrated that increased levels of RIP1K in reactive astrocytes are associated with astrogliosis and glial scar formation.^[^ ^57^ ^]^ It has been reported that following cerebral ischemia, apoptosis is largely confined to neurons in the ischemic core. However, the upregulation of caspase‐3 and several other caspase family members is seen in astrocytes and microglia after MCA occlusion, and DNA strand breakage is also observed in the astrocytes of the peri‐infarct area. Concurrently, Bcl‐2 (B‐cell lymphoma‐2), an anti‐apoptotic protein, is found increased in ischemic astrocytes.^[^ ^58^ ^]^ These findings indicate that neurons and astrocytes may have different resistances to necroptosis and apoptosis, but the detailed insightful investigations remain to be explored.

The intracellular labile iron pool is controlled by the uptake, export, storage, and utilization of iron. Cellular iron uptake is primarily modulated by the Tf‐TfR system. This system has been shown to play a role in ferroptosis.^[^ ^25^ ^]^ TfR1 is a key transport receptor for cellular iron uptake, thus we examined changes of TfR1 in astrocytes after ischemic stroke and its relationship with iron overload. We found that in both the acute and subacute period of ischemic stroke, the brain TfR1 and Fe^2+^ sustained higher levels. In addition, the immunostaining of TfR1 in astrocytes was markedly increased from 3 d to 14 d post‐I/R, accompanied by a large amount of iron deposition at d 14 post‐I/R. By contrast, both *Tfrc^+/−^* and astrocyte‐specific conditional *Tfrc* knockdown mice presented reduced infarction volume, improved neurological deficits, as well as declined level of brain Fe^2+^ and iron deposition in astrocytes, and decreased glial scar thickness in the peri‐infarct area. These data indicate that the increased TfR1‐mediated iron overload in astrocytes contributes to ischemic stroke‐induced brain injury.

Palmitoylation is a post‐translational lipid modification of proteins, and it can alter the localization, stability, and function of target proteins. Several studies have demonstrated that dysregulation of TfR1 palmitoylation affects Tf‐TfR1 recycling, leading to cellular iron overload in neurodegenerative diseases.^[^ ^30^, ^31^ ^]^ In addition, the palmitoylation of TfR1 accelerates its intra‐Golgi transport and Tf‐TfR1 recycling from endoplasmic reticulum to plasma membrane.^[^ ^38^ ^]^ Using a CCR‐based method, we revealed that both total TfR1 palmitoylation and TfR1 palmitoylation on the cell membrane were increased in OGD/Re‐treated HA cells, and a well‐known inhibitor of protein S‐palmitoylation, 2‐BP treatment could significantly reduce the level of total TfR1, total TfR1 palmitoylation, and the localization of palmitoylated TfR1 on the cell membrane, as well as the Fe^2+^ level. In addition, 2‐BP could reduce the astrocytic iron overload and inhibit astrogliosis in the peri‐infarct area 14 d after I/R. It has been confirmed that the palmitoylation sites of human TfR1 are in Cys^62^ and Cys^67^.^[^ ^38^, ^39^ ^]^ Similarly, the mutation of Cys^62^ and Cys^67^ for TfR1 suppressed the palmitoylation of TfR1 and decreased the levels of Fe^2+^, ROS, and lipid peroxidation in OGD/Re‐treated HA cells. These results suggest that inhibition of TfR1 expression or reduction of TfR1 palmitoylation can reduce the iron overload of astrocytes, causing reduced reactive proliferation of astrocytes. Under physiological conditions, the Tf‐bound iron through TfR1 (Tf‐TfR1 complex) internalizes into the cell by clathrin‐mediated endocytosis, upon its binding at the cell surface.^[^ ^40^ ^]^ So far, it is unclear whether palmitoylation of TfR1 affects clathrin‐mediated TfR1 endocytosis and uptake of iron. We elucidated that both 2‐BP treatment and double mutations in Cys^62^ and Cys^67^ of TfR1 reduced OGD/Re‐mediated co‐localization between TfR1 and clathrin, indicating that palmitoylation of TfR1 promotes clathrin‐mediated TfR1 endocytosis, enhancing iron uptake. Moreover, we identified that supplement iron with FAC increased the co‐localization of TfR1 and clathrin in HA cells, indicating excessive iron accelerates clathrin‐mediated TfR1 endocytosis.

Palmitic acid, a saturated fatty acid, is the major fatty acid providing substrates for protein lipidation, such as palmitoylation. Palmitic acid converts to palmitoyl‐CoA and then S‐acyl incorporates into the cysteine residues of protein via labile thioester bonds. This reversible process is known as S‐palmitoylation. We hypothesized that ischemic stroke‐induced fatty acid disorder could increase palmitate and secondarily affect palmitoylation of TfR1.^[^ ^43^, ^44^ ^]^ Carnitine palmitoyl transferase 1 (CPT1) is a crucial rate‐limiting enzyme of fatty acid β‐oxidation. CPT1A, the liver isoform, is located in the outer membrane of mitochondria, by which acyl‐coenzyme converts into acyl‐carnitines, and then acyl‐carnitines cross membranes to get into the mitochondria for β‐oxidation. We next used conditional astrocytic *Cpt1a* heterozvgous knockout (*Cpt1a^As+/−^*) mice or CPT1 inhibitor etomoxir to induce astrocytic fatty acid disorder.

The lipidomics analysis by GC‐MS method identified an increase in palmitic acid level in OGD/Re‐treated HA cells, and etomoxir treatment further enhanced OGD/Re‐mediated increase in level of palmitic acid of HA cells. *Cpt1a^As+/−^* mice or *Cpt1a^As+/−^* astrocytes or etomoxir‐treated HA cells displayed increased TfR1 palmitoylation level and its membrane localization as well as clathrin‐mediated TfR1 endocytosis, accompanied by an increase in the levels of Fe^2+^, ROS, and lipid peroxidation, as well as the levels of GPX4 and SLC7A11 in astrocytes. In contrast, *Cpt1a*‐overexpressing HA cells presented decreased palmitoylation of TfR1 on the cell membrane and clathrin‐mediated TfR1 endocytosis as well as reduced Fe^2+^ level. These results collectively suggest that CPT1A dysfunction in astrocytes promotes TfR1 palmitoylation and clathrin‐mediated endocytosis of TfR1 by providing palmitic acid, accelerating the iron uptake, which, in turn, causes iron overload‐mediated high expression of GPX4 and SLC7A11, ROS production, and lipid peroxidation in astrocytes, finally inducing astrogliosis. Furthermore, our results showed that supplementing cultured HA cells with exogenous palmitic acid resulted in increased TfR1 palmitoylation and intracellular Fe^2+^ level, and enhanced proliferation of HA cells. We cannot rule out that the increase of palmitic acid might influence other cellular pathways, such as inflammation or lipid signalling, leading to the proliferation of astrocytes and astrogliosis in ischemic stroke. For example, palmitic acid increases proinflammatory cytokine production by activating the Toll like receptors (TLR) 2‐ and 4‐mediated signaling pathway and promoting the generation of ROS. In the cell, palmitic acid metabolizes into saturated diacylglycerols, ceramides, and lysophosphatidylcholine, which lead to inflammatory reactions.^[^ ^59^, ^60^ ^]^ Palmitic acid, as a medium‐long chain saturated fatty acid, is an important component involved in lipid metabolism. Excessive palmitic acid can disrupt the balance of fatty acid metabolism, causing various metabolic disorders such as reduced stability of cell membranes, lipid droplet accumulation, and cellular senescence^.[^ ^44^, ^61^ ^]^ All these effects mediated by palmitic acid might also involve in the proliferation of astrocytes and astrogliosis in ischemic stroke.

Protein S‐palmitoylation is dynamically regulated by a class of polytopic transmembrane proteins called protein acyltransferases with zinc‐finger and aspartate‐histidine‐histidine‐cysteine domains (DHHCs) and depalmitoylases (APTs, such as PPT1/2). Recently, a study identified downregulation of DHHC5‐mediated TfR1 palmitoylation in oligodendrocytes is related to neonatal sevoflurane exposures‐associated neurotoxicity.^[^ ^62^ ^]^ How DHHC5‐mediated palmitoylation of TfR1 in astrocytes participates in ischemic stroke‐induced iron overload and brain damage remains to be investigated in the very near future.

We finally demonstrated that application of an antioxidant Fer‐1, or iron‐chelating agent DFO, or iron deficiency in circulation of mice, all could reduce the levels of brain iron and pro‐ferroptotic factors, and increase antioxidant factors, protecting neurons and astrocytes and depressing astrogliosis. Interestingly, Fer‐1 and DFO could increase the expression of GPX4 and SLC7A11 in neurons after OGD/Re but had no significant effect on the expression of GPX4 and SLC7A11 in astrocytes. Iron overload leads to ROS accumulation in cells via the Fenton reaction.^[^ ^19^ ^]^ Once the overproduction of ROS exceeds the toxicity threshold or the antioxidant system is impaired, the cancer cells undergo death; maintaining ROS levels below the toxicity threshold promotes cancer cells survival and proliferation.^[^ ^63^ ^]^ In our studies, we found that after OGD/Re treatment, the high level of ROS and the decreased expressions of Nrf‐2/GPX4, SLC7A11 in neurons lead to ferroptosis, however, the ROS level which was below the toxicity threshold and the increased expressions of Nrf‐2/GPX4, SLC7A11 lead to reactive proliferation of astrocytes. Antioxidants and iron chelators can reduce the level of ROS in ischemic neurons and increase the levels of GPX4 and SLC7A11, protecting neuronal ferroptosis. Although it had no significant effect on the levels of GPX4 and SLC7A11 in OGD/Re‐treated astrocytes with Fer‐1 or DFO treatment, the level of ROS in astrocytes was reduced, thereby reducing the astrogliosis. DFO is an iron chelator with FDA approval for the clinical treatment of acute iron overload, and a phase II clinical trial (Phase II, NCT00777140) in patients with ischemic stroke has been completed. During the tPA infusion, placebo or a single DFO bolus followed by a 72 h continuous infusion of three escalating doses was administrated, the promising results showed that DFO at 40–60 mg kg^−1^d^−1^ can rescue neurological deficits in patients with moderate‐severe ischemic stroke (NIHSS > 7), and no thrombolysis complications were found.^[^ ^16^ ^]^ However, the short half‐life (5–17 min) in serum/plasma limits its clinical application,^[^ ^16^ ^]^ thus improving its structure or transforming it into a sustained‐release formulation may increase the half‐life and reduce the side effects, which will facilitate its clinical transformation for ischemic stroke therapy. Several novel iron chelators are under pre‐clinical investigation, for example, BHAPI is a kind of iron chelating agent, which only exerts strong free‐iron binding capacity under the condition of oxidative stress and restores the mitochondrial energy utilization to improve cell vitality in OGD‐treated HT22 cells.^[^ ^64^, ^65^ ^]^ Derivative 11, a derivative of deferasirox and celecoxib synthesized with their active groups retained, promotes neuroprotection in PC12 cells and reduces the infarct volume and neurological deficits in rats.^[^ ^66^ ^]^

This study identifies iron overload‐mediated similar oxidative stress changes but opposite expressive patterns in endogenous antioxidant defense systems of Nrf‐2/GPX4 and SLC7A11 between neurons and astrocytes as a central mechanism, causing divergent ferroptotic outcomes with neurons being more vulnerable to ferroptosis, while astrocytes display greater resistance in ischemia/reperfusion insults. We reveal that elevated palmitoylation of TfR1 drives clathrin‐mediated TfR1 endocytosis and uptake of extracellular iron, leading to intracellular iron overload in astrocytes. We uncover ischemia/reperfusion‐induced fatty acid accumulation, especially palmitic acid is a key regulator of TfR1 S‐palmitoylation. These findings will advance a new concept for cell type‐specific ferroptosis profile and offer promising strategies for the development of therapeutic approaches to lowering ischemic stroke‐induced iron overload, including application of iron chelator or antioxidant, suppression of TfR1 S‐palmitoylation, as well as improvement of fatty acid accumulation.

## 4 Experimental Section

### Patients and the Detection of N‐acetylaspartate and Iron Concentration

Three ischemic stroke patients were included, with ethics approval from Renji Hospital, Shanghai, China (LY2024‐186‐B). Written informed consent was obtained from all participants. All scans were performed using a 3T Siemens Prisma MR scanner. Initial MRI scans were performed upon admission, with symptom onset to scan times ranging from 9.5 to 60.5 h, followed by a second MRI scan 5 to 6 days later. To simultaneously evaluate neuronal loss/dysfunction and iron deposition, high‐resolution 3D MRSI technology was utilized to map N‐acetylaspartate (NAA) as a marker of neuronal integrity and quantitative susceptibility mapping (QSM) for iron concentration.^[^ ^67^ ^]^ The scanning sequence included SPICE (TR/TE = 160/1.6 ms, MRSI resolution = 2 × 3 × 3 mm^3^, QSM resolution = 1 × 1 × 1 mm^3^, FOV = 240 × 240 × 72 mm^3^, 7:46 min), DWI (TR/TE = 2500/72 ms, resolution = 2.0 × 2.0 × 2.0 mm^3^, FOV = 256 × 256 × 144 mm^3^, b = [0,1000] s/mm^2^), FLAIR (TR/TE/TI = 9000/89/2500 ms, resolution = 0.9 × 1.3 × 2.0 mm^3^, FOV = 240 × 240 × 150 mm^3^), and 3D MPRAGE (TR/TE/TI = 2400/2.13/1100 ms, resolution = 1.0 × 1.0 × 1.0 mm^3^, FOV = 256 × 192 × 256 mm^3^).

High‐resolution QSM was simultaneously acquired with metabolic imaging using the SPICE acquisition.^[^ ^67^ ^]^ The spatiospectral distributions of the metabolites were reconstructed using the original SPICE processing pipeline.^[^ ^68^, ^69^, ^70^, ^71^, ^72^, ^73^, ^74^ ^]^ QSM maps were calculated using an existing processing pipeline, which included field estimation using Hankel singular value decomposition, background field removal by solving the Laplacian boundary value problem, and solving the dipole‐inversion model incorporating anatomical spatial priors.^[^ ^67^, ^75^, ^76^, ^77^ ^]^ The estimated NAA concentrations were normalized using the water reference.

The ischemic lesion in the acute stage scan was manually delineated on DWI images, and on FLAIR images for subacute scan, by a neuroradiologist (T.W., with 10 years of experience). All FLAIR, DWI, NAA, and QSM images, along with the corresponding tissue masks, were co‐registered to the T1‐weighted image using Advanced Normalization Tools (ANTs, http://stnva.github.io/ANTs/). The cortical gray matter mask was segmented on the T1‐weighted image using SynthSeg in FreeSurfer (v7.4.1, https://surfer.nmr.mgh.harvard.edu). The perilesional cortex, defined as the cortical regions adjacent to the lesion, was manually outlined based on the FreeSurfer segmentation. Group‐level statistical analyses for NAA and tissue susceptibility were conducted using the Mann–Whitney U‐test to compare voxels in the perilesional cortex and the contralateral cortex. All statistical analysis was performed using SPSS 24 (IBM).

### Animals

*WT* C57BL/6 mice (22–25 g) and Sprague‐Dawley rats (280–320 g) were purchased from Experimental Animal Center of Soochow University and approved by the University Committee on Animal Care of Soochow University (Use license: SYXK‐2021‐0065; Production license: SCXK‐2022‐0008). *Cpt1a^flox/flox^* (*Cpt1a^fl/fl^*) mice and *GFAP‐cre* mice were purchased from Shanghai Model Organisms Center Inc. (Shanghai, China). Ttransferrin receptor 1 gene *Tfrc* heterozygote knockout (*Tfrc^+/−^*) mice were purchased from Cyagen Biosciences Inc. (Santa Clara, CA, US). Conditionally overexpress the liver *Hepcidin Antimicrobial Peptide gene* (*Hamp*) mice were provided by Xue‐Chu Zhen's lab (College of Pharmaceutical Science, Soochow University). All the animals were housed under a 12‐h light/dark cycle and fed with a standard diet. All the procedures were in strict accordance with the Chinese legislation on the use and care of laboratory animals and guidelines established by the Laboratory Animal Center of Soochow University and were approved by the Institutional Animal Care and Use Committee of Soochow University, Soochow, China (#202306A0924, # 202112A0121, # 202205A0204).

Conditional astrocytic *Cpt1a* (*Carnitine palmitoyltransferase 1 A gene)* heterozvgous knockout mice (*Cpt1a^As+/−^, Cpt1a^fl/wt^ GFAP‐Cre^+^*) mice were constructed using *Cpt1a^fl/fl^* mice crossed with mice expressing Cre recombinase under the control of the astrocytic‐specific glial‐fibrillary acidic protein (GFAP) promoter on a C57BL/6 background. Overview of genetically engineered strategies for these genetically modified mice are1shown in Figure S7 Supporting Information), and the primers for identification of *Cpt1a^As+/−^*, *Tfrc^+/−^* mice and *Hamp* mice are listed in Table S1 (Supporting Information).

### Rodent Models of Cerebral Ischemia and Reperfusion (I/R) Injury

Transient middle cerebral artery occlusion was used to induce cerebral ischemia/reperfusion (I/R) injury.^[^ ^78^, ^79^ ^]^ With anesthetic treatment, transient focal brain ischemia was induced by inserting a 4‐0 for rats or 6‐0 monofilament for mice (Doccol Corporation, Sharon, MA, USA) into the right internal carotid artery via the common carotid artery and was advanced until the tip of the silicone coated monofilament occluded the origin of the right middle cerebral artery (MCA). For reperfusion, the suture was gently withdrawn 90 min for rats or 60 min for mice after ischemia (I/R). Regional cerebral blood flow (rCBF) was detected with Laser speckle blood flow imaging system (RFSLI ZW/RFLSI, RWD, China), and any animals that exhibited less than a 70% decrease in rCBF following occlusion relative to baseline, as well as those with brain hemorrhage or died during or following surgery, were excluded from the analysis. Sham‐operated mice underwent the same procedures without MCA occlusion. Rectal temperature was maintained at 37 ± 0.5 °C using a heating pad throughout the procedure.

Rats or mice were randomly assigned to groups using the online tool Quickcalcs (http://www.graphpad.com/quickcalcs/). Six independent experiments were performed. In the rat experiments, rats were divided into two groups: Sham and I/R, mitochondrial morphology in neurons or astrocytes was examined by transmission electron microscopy at d 7 post‐I/R. In the *Tfrc^+/−^* mice experiments, mice were divided into three groups: Sham *WT*, I/R *WT*, I/R *Tfrc^+/−^* group, the infarct volume, neurological deficit score, and Fe^2+^ level were measured at d 1 post‐I/R, and immunohistochemistry or iron deposition was measured at d 14 post‐I/R. In the 2‐BP treatment experiments, mice were divided into three groups: Sham, I/R, and I/R+2‐BP, and in the *Cpt1a^As+/−^* mice experiments, mice were divided into four groups: Sham *WT*, Sham *Cpt1a^As+/−^*, I/R *WT*, and I/R *Cpt1a^As+/−^*, and the immunohistochemistry or iron deposition was measured at d 14 post‐I/R. In the Fer‐1 and DFO treatment experiments, mice were divided into four groups: Sham, I/R, I/R+Fer‐1, and I/R+DFO, and in the *Hamp* mice experiments, mice were divided into three groups: Sham *WT*, I/R *WT*, and I/R *Hamp*. Immunohistochemistry, iron deposition, and the level of ferroptosis‐related factors was measured at d 14 post‐I/R, and neurological deficits were measured at d 1, d 3, d 7, and d 14 post‐I/R. All experiments were conducted by researchers blinded to the experimental groups.

For drug treatment, The Fer‐1 (HY‐100579), DFO (HY‐B1625), and 2‐BP (HY‐111770) were purchased from MedChemExpress. Tamoxifen (T6906) was purchased from TargetMol. The Fer‐1, DFO, and 2‐BP were dissolved in DMSO and diluted with saline, and the final concentration of DMSO was 0.1%. Fer‐1 (1 mg kg^−1^), DFO (50 mg kg^−1^), or 2‐BP (30 mg kg^−1^) were injected via the tail vein at 1 d post‐I/R, once every two days, and the treatment lasted until 14 d after I/R. For *Hamp* mice, 0.1 mL Tamoxifen (10 mg mL^−1^, dissolved in sunflower oil) were intraperitoneally injected to mice for 5 d before experiment.

### Establishment of Astrocyte‐Specific Conditional Tfrc Knockdown (Tfrc cKD) Mice

*Tfrc* cKD mice were established via astrocyte‐targeted adeno‐associated viruses (AAVs, AAV2/PHP. eB‐GfaABC1D‐EGFP‐shCtrl/shTfrc‐tWPA) transfection. AAVs were purchased from OBiO Technology (Shanghai) Corp., Ltd., and were given to mice (5 × 10^11^ TU for one mouse, PBS dilution) by intravenous injection. The transfection was confirmed 5–6 days after the injection by immunohistochemistry (Figure 5e).

### Assessment of Neurological Deficits

Neurological deficits scores, grip strength tests, cylinder test, and adhesive removal (sticky‐tape) test were used to assess neurological deficits as previously described.^[^ ^80^, ^81^ ^]^

### Assessment of Neurological Deficits—Neurological Deficit Scores

0, no obvious neurological deficits; 1, failure to fully extend contralateral forepaw; 2, walking persistently in large circles toward the ipsilateral side; 3, walking persistently in small circles and/or repeatedly rolling over toward the ipsilateral side; 4, no spontaneous motor activity and low level of consciousness; 5, died after awakening.

### Assessment of Neurological Deficits—Cylinder Test

Cylinder test was used to check the asymmetry in forelimb movement. Mice were placed into a clear cylinder (HOOFAN, Wenling, China), and the forelimb‐use asymmetry was recorded during their vertical movements along the cylinder wall. The first 20 movements were recorded during a 10‐min test, and the final cylinder score was calculated as follows: (Right forelimb movement – Left forelimb movement) / (Right forelimb movement + Left forelimb movement + Both movements) ×100%.

### Assessment of Neurological Deficits—Adhesive Removal Test

The adhesive removal test was assessed for forepaw sensitivity and motor impairments. The tape strips (0.3 cm × 0.4 cm) were applied to the paw of each animal with equal pressure so that it covered the hairless part of the forepaw to evaluate the mice's sensory and motor function after I/R. The time for the mouse to touch and remove the tape was measured up to 120 s. Data were presented as the mean value of three trials. Data measured 1d before I/R were recorded as the preoperative data (pre). Repeat testing on the adhesive removal test was performed 1 d, 3 d, 7 d and 14 d after I/R.

### Assessment of Neurological Deficits—Grip Strength Test

Forelimb grip strength was evaluated with a mouse grip strength meter (YLS‐13A, Jinan YiYan Technology Development Co., Ltd, China). Each mouse was lifted by the tail until they grabbed the pull‐bar with both forepaws, and the peak force was recorded (strength in grams) and the data were presented as the mean value of 10 trials for each mouse.

### 2,3,5‐Triphenyltetrazolium Chloride (TTC) Staining

To observe cerebral infarction, the forebrains were divided into five coronal sections (2 mm) using a mouse brain matrix (Harvard apparatus); then, the sections were subsequently incubated in 2% TTC (Sigma–Aldrich, USA) at 37 °C for 15 min followed by fixation in 4% paraformaldehyde in PBS (pH 7.4). The volume of infarction was calculated by multiplying the distance between sections, and the data was expressed as the percentage of the right brain volume.

### Transmission Electron Microscopy

Transmission electron microscopy was used to analyze mitochondrial morphology after I/R as previously described.^[^ ^82^ ^]^ After I/R 7 d, cubic millimeter brain fragments were harvested from the peri‐infarct area of the rat cortex and postfixed in 1% osmium tetroxide in 0.1 mol L^−1^ phosphate buffer (pH 7.4) for 1 h, dehydrated in a graded ethanol series, and flat embedded in epoxy resin. Ultrathin sections (40‐ to 60‐nm‐thick) were cut with a Reichert ultramicrotome and placed on grids (200 mesh), stained with uranyl acetate and lead citrate, and then observed under a Philips CM‐120 electron microscope.

### Cell Culture, Oxygen‐Glucose Deprivation/Re‐Oxygenation (OGD/Re) and Cell Death/Viability Assay—Cell Line Culture

Human astrocytes cell line HA1800 (C1244) and mouse hippocampal neuronal cell line HT22 (C1026) were purchased from WHELAB (Shanghai, China), and cultured in Dulbecco's modified Eagle's medium (DMEM) (C11995500BT, Gibco, USA) supplemented with 10% fetal bovine serum (10099, Gibco, USA) and 1% penicillin–streptomycin (C0222, Beyotime, China) solution. The cells were maintained at 37 °C in 5% CO_2_. The medium was changed every 2–3 days until the cells reached 80–90% confluence, and the cells were passaged for further experiments.

### Cell Culture, Oxygen‐Glucose Deprivation/Re‐Oxygenation (OGD/Re) and Cell Death/Viability Assay—Primary Cerebral Cortical Neuron and Astrocyte Culture

The cerebral cortices of C57BL/6 mouse neonates (1 day old) were digested with 0.25% trypsin for ≈10 min at 37 °C and terminated using a complete culture medium. Then the medium was removed, and the tissue was resuspended with 10 mL complete culture medium, and added the DNase solution and incubated at room temperature for 5 min. Aspirated the medium and washed the tissue twice gently with 10 mL complete culture medium, then filtered with a sterile 40 µm nylon cell strainer. For neuron culture, the cells were cultured in neurobasal medium (Gibco, 21103‐049) supplemented with 2% B‐27 (Gibco, 17504‐044), 0.5 mm L‐Glutamine (Sigma–Aldrich, G8540), and 100 U mL^−1^ penicillin/streptomycin (Beyotime Biotechnology, C0222). For astrocyte culture, the cells were cultured in DMEM/F12 (Gibco, 11330) containing 10% fetal bovine serum (Gibco; 10099) and 100 U mL^−1^ penicillin/streptomycin (Beyotime Biotechnology, C0222). Then the neurons and astrocytes were incubated under a humidified atmosphere containing 5% CO_2_ at 37 °C. The purity of astrocytes was confirmed as being more than 95% by immunocytochemistry with MAP2, a neuronal marker protein, or GFAP, an astrocytic marker protein, respectively.

### Cell Culture, Oxygen‐Glucose Deprivation/Re‐Oxygenation (OGD/Re) and Cell Death/Viability Assay—Oxygen‐Glucose Deprivation (OGD) and OGD/Re‐Oxygenation (OGD/Re)

Upon OGD treatment, the medium was removed and replaced with glucose‐free DMEM (Gibco, 11966), and the cells were placed in a sealed chamber (Billups‐Rothenberg, CA, USA) containing mixed gas (95% N_2_ and 5% CO_2_) for the indicated time. Upon re‐oxygenation treatment, cells were taken out of the chamber after OGD for the indicated time and transferred to the regular cell culture incubator, and the medium was replaced with complete medium. Control cells were incubated in glucose‐containing DMEM and received similar wash steps as the OGD/Re‐treated cells in a humidified atmosphere containing 5% CO_2_ at 37 °C.

Five independent experiments were performed. In Fer‐1 and DFO treatment experiments, HA cells were divided into six groups: non‐OGD, non‐OGD + Fer‐1, non‐OGD + DFO, OGD/Re and OGD/Re + Fer‐1, OGD/Re + 2‐DFO. In 2‐BP treatment experiments, HA cells were divided into four groups: non‐OGD, non‐OGD + 2‐BP, OGD/Re, and OGD/Re + 2‐BP. In astrocyte‐specific *Cpt1a* heterozygous knockout experiments, primary cultured *WT* and *Cpt1a^As+/−^* mice astrocytes were divided into four groups: non‐OGD WT, non‐OGD *Cpt1a^As+/−^*, OGD/Re WT and OGD/Re *Cpt1a^As+/−^*. In inhibit of Nrf‐2 experiments, Cells were divided into four groups: non‐OGD, non‐OGD + ML385, OGD/Re, OGD/Re + ML385. In iron overload experiments, cells were treated respectively with different concentrations of FAC or hemin for 24 h, including 1.56, 3.13, 6.25, 12.5, 18.75, 20, 25, 50, 100, 200, and 400 µm. Cell viability assays, Immunofluorescence, Western Blotting, Ferroptosis assay, and palmitoylated TfR1 assays were performed at h 24 after reoxygenation or drug treatment.

For drug treatment, The Fer‐1, DFO and 2‐BP, ML385 (HY‐100523), Ammonium ferric citrate (HY‐B1645), Hemin (HY‐19424), and Etomoxir (HY‐50202) were purchased from MedChemExpress. Fer‐1, DFO, 2‐BP, Etomoxir (Eto), ML385, ammonium ferric citrate (FAC), and Hemin were dissolved in DMSO to prepare a stock solution (1000×) based on the experimental design. These agents were diluted to the required final concentrations in the medium and administered to cells upon re‐oxygenation.

### Cell Culture, Oxygen‐Glucose Deprivation/Re‐Oxygenation (OGD/Re) and Cell Death/Viability Assay—Lactate Dehydrogenase (LDH) Leakage Measurement

To determine cell injury, the lactate dehydrogenase (LDH) leakage from the cell was detected using a lactate dehydrogenase (LDH) assay kit (A020, Nanjing Jiancheng, China), performed according to the manufacturer's manual, and measured at 450 nm using a multimode microplate reader, Infinite M1000 PRO (Tecan Trading AG, Switzerland).

### Cell Culture, Oxygen‐Glucose Deprivation/Re‐Oxygenation (OGD/Re) and Cell Death/Viability Assay—Cell Viability Assay

Cell viability was assessed by using a cell counting kit‐8 (CCK‐8, MedChemExpress, USA). The CCK‐8 solution was added to cells in each well (1:10), and the cells were incubated for 1 h in the incubator, then the absorbance was measured at 450 nm using a microplate reader.

### PI (Propidium Iodide) Staining

In vivo study, the PI (P4170, Sigma–Aldrich, USA) was dissolved in DMSO and diluted into saline at a final concentration of 10 mg mL^−1^ and was injected intraperitoneally (15 mg kg^−1^) to mice at 1 h before sacrifice. The brains were performed frozen section into 16‐mm thick slices in the coronal plane. Subsequently, immunohistochemical staining of GFAP and NeuN was used to observe the death of astrocytes and neurons in the peri‐infarct area. Images were obtained using a confocal laser scanning microscopy (LSM 710, Carl Zeiss, Germany, 40 × dry objective, pixel sizes: 2048 × 2048; AIR HD25, Nikon, Japan, 20 × dry objective, pixel sizes: 1024 × 1024).

In vitro study, the solid PI was dissolved in DMSO to a final concentration of 5 mg mL^−1^ and stored in the dark. PI solution (1:1000, final concentration 5 µg mL^−1^) and Hoechst staining solution (1: 1000, #33258, Sigma–Aldrich, MO, United States) were added to the cells, and the cells were stained for 20 min. Then images were obtained using fluorescence microscope (IX73, Olympus, Japan, 20/40 × dry objective, pixel sizes: 1920× 1200 or 1024 × 1024).

For the statistical analysis of PI staining, the images were imported into FIJI ImageJ for cell counting and subsequent statistical analysis. The cell numbers of both PI^+^ GFAP^+^, PI^+^ NeuN^+^, total PI^+^, total GFAP^+,^ and total NeuN^+^ cells were recorded, and the ratio of PI^+^ NeuN^+^ cells /total NeuN^+^ cells, PI^+^ GFAP^+^ cells /total GFAP^+^ cells, and PI^+^ NeuN^+^ or PI^+^ GFAP^+^ cells /total PI^+^ cells were analyzed.

### RNA‐Sequencing (RNA‐Seq) and Analysis

HA cells were treated with 3.13 µm FAC, and the RNA‐seq was performed by Lianchuan Biotechnology Co., LTD (Hangzhou, China). Total RNA was isolated using TRIzol reagent (Invitrogen, Carlsbad, CA, USA), and the RNA integrity was assessed by Bioanalyzer 2100 (Agilent, CA, USA). Poly (A) RNA was captured from total RNA using Dynabeads Oligo (dT) (Thermo Fisher, 25–61005) and fragmented into small pieces using the Magnesium RNA Fragmentation Module (NEB, E6150), then reverse‐transcribed to cDNA by SuperScript II Reverse Transcriptase (Invitrogen, 1896649, USA), which were next used to synthesize U‐labeled second‐stranded DNAs with E. coli DNA polymerase I (NEB, m0209, USA), RNase H (NEB, m0297, USA) and dUTP Solution (Thermo Fisher, R0133, USA). The U‐labeled second‐stranded DNAs were treated with UDG enzyme (NEB, cat.m0280, USA) and amplified by PCR. The average insert size for the final cDNA library was 300 ± 50 bp. Finally, the paired‐end sequencing (PE150) was performed on an Illumina Novaseq 6000 sequencer (LC‐BioTechnology CO., Ltd., Hangzhou, China).

Using the Illumina paired‐end RNAseq approach, the transcriptome was sequenced, generating a total of million 2 × 150 bp paired‐end reads. Reads obtained from the sequencing machines include raw reads containing adapters or low quality bases. Thus, to get high quality clean reads, reads were further filtered by Cutadapt (https://cutadapt.readthedocs.io/en/stable/, version: cutadapt‐1.9). Then sequence quality was verified using FastQC (http://www.bioinformatics.babraham.ac.uk/projects/fastqc/, 0.11.9). Further, HISAT2 (https://daehwankimlab.github.io/hisat2/, version: hisat2‐2.2.1) was used to map reads to the reference genome of Homo sapiens GRCh38. The mapped reads were assembled using StringTie (https://ccb.jhu.edu/software/stringtie, version: stringtie‐2.1.6) with default parameters. Then, all transcriptomes from all samples were merged to reconstruct a comprehensive transcriptome using gffcompare (https://github.com/gpertea/gffcompare/, version: gffcompare‐0.9.8). After the final transcriptome was generated, StringTie was used to estimate the expression levels of all transcripts and perform expression level for mRNAs by calculating FPKM (FPKM = [total_exon_fragments/mapped_reads (millions) × exon_length (kB)]). The differentially expressed mRNAs were selected with fold change > 2 or fold change < 0.5 and with parametric F‐test comparing nested linear models (*p* value < 0.05) by R package DESeq2. the proliferative genes of the control and FAC groups were sorted based on log2FC their values, and then created a heatmap to show gene expression pattern in the two groups.

### Western Blotting Analysis

Proteins were separated on 8–12% SDS–PAGE gels and transferred to polyvinylidene fluoride (PVDF) membrane. The membranes were blocked in 5% BSA and then incubated with specific primary antibodies (Table S2, Supporting Information) overnight at 4 °C. Then, the membranes were washed and incubated with secondary antibodies (Table s3, Supporting Information) for 1 h at room temperature and washed with TBST. Blots were captured by Odyssey scanner (LI‐COR Biosciences, Lincoln, NE, USA). Densitometric analysis of the bands was quantitatively analyzed using ImageJ software.

### Immunohistochemistry and Immunofluorescence

Brain slices or cells were fixed with 4% paraformaldehyde for 10 min, permeabilized with Triton X‐100 (0.3% for brain slices or 0.1% for cells) for 20 min, blocked by 1% BSA for 1 h at room temperature, and incubated with specific primary antibodies (Table S2, Supporting Information) overnight at 4 °C. The brain slices or cells were then incubated with the corresponding secondary antibodies (Table S3, Supporting Information) for 1 h at room temperature. Hoechst (1:5000, 33258, Sigma–Aldrich, USA) was then applied to stain cell nuclei. The images were captured by a confocal microscope (LSM 710, Carl Zeiss, Germany, 40 × dry objective, pixel sizes: 2048 × 2048; AIR HD25, Nikon, Japan, 20 × dry objective, pixel sizes: 1024 × 1024) or fluorescence microscope (IX73, Olympus, Japan, 20/40 × dry objective, pixel sizes: 1920 × 1200 or 1024 × 1024).

The brain sections were stained with immunofluorescence to investigate the GFAP levels to detect the glial scar thickness. Glial scar thickness = Area of the glial scar/Total boundary length of glial scar × 2.

For the statistical analysis of immunofluorescence intensity, the images were input into FIJI ImageJ software, and the mean fluorescence intensity (A.U.)/µm2 in a region of interest (ROI) was measured, and statistical data were expressed as mean fluorescence intensity (fold change), normalized relative to the sham/control group. FIJI ImageJ software colocalization finder was also used to analyze the colocalization of double‐stained images. The Mander's overlap coefficient indicates the actual overlap between color channels, which represents the true level of colocalization. In the range of values from 0 to 1, the results were as follows: 1 indicates complete overlap, and 0 indicates no overlap. The Mander's overlap coefficients were suitable when the fluorescence of one antigen was stronger than that of another antigen. The Pearson correlation coefficient was used to measure the actual overlap between color channels, in the range of values from −1 to 1. The results were as follows: 1 indicates a perfect positive correlation, 0 indicates no significant correlation, and −1 indicates a perfect negative correlation.

### Nissl Staining

Paraffin sections of brains were immersed in xylene, absolute ethanol, 95% ethanol, and 70% ethanol for 5 min each time, and distilled water for 2 min. The sections were stained with Nissl's staining solution (C0117, Beyotime, China) for 10 min and washed twice with distilled water. After 70% ethanol, 95% ethanol dehydrated for 2 min, then use transparent xylene for 5 min, neutral resin sealing piece.

### Alkaline Phosphatase Kit Labels Astrocytes

The brain frozen sections were rinsed with PBS for 10 min and a mixture of 0.3% hydrogen peroxide for 10 min. Brain sections were blocked with 10% goat serum for 30 min and incubated overnight at 4 °C with the primary antibody of GFAP. After washing with PBS, the Rabbit Anti‐Chicken IgM/AP (K0050R‐AP, Solarbio, China) working solution (1 µg/1 mL) was added and incubated at 37 °C for 1 h, and GFAP was stained with an alkaline phosphatase kit (E104, Applygen, China).^[^ ^83^ ^]^

### Perl's‐DAB Ferric Iron Staining

The brain sections were incubated with Perl's staining solution at 37 °C for 30 min, and were processed with incubation solution for 30 min and enhancement solution for 30 min according to the Perl's iron staining kit (G1428, Solarbio, China), washed with PBS, and sealed. The sections were examined under a brightfield microscope (IX73, Olympus, Japan, 20/40 × dry objective, pixel sizes: 1920 × 1200). Black or brown granules were regarded as positive staining of deposition of iron in the tissue section.^[^ ^83^ ^]^

### Ferro Orange, DCFH‐DA, and Liperfluo Staining

The levels of ROS, lipid peroxidation, and Fe^2+^ in cells were measured by Ferro Orange (F374, Dojindo, Japan), DCFH‐DA (S0033S, Beyotime, China), and Liperfluo (L248, Dojindo, Japan), respectively. Ferro Orange, DCFH‐DA, and Liperfluo solution (Manual recommended concentration) and Hoechst staining solution (1: 1000) were added to the cells, and the cells were stained for 30 min, and observed under a fluorescence microscope (IX73, Olympus, Japan, 20/40 × dry objective, pixel sizes: 1920 × 1200). Mean fluorescent intensities were measured in images stained with Ferro Orange, DCFH‐DA, and Liperfluo, and data were then normalized to the averaged values of the control group, and the results presented as fold change.

### Quantitative ROS Detection

The tissue samples or cell samples were weighed accurately and lysed in PBS, centrifuged at 100×g for 3 min at 4 °C, and the supernatant was added with 1 µm DHE (Dihydroethidium, S0063, Beyotime, China) for incubation at 37 °C, for 30 min. The fluorescence intensity was measured at an excitation wavelength of 300 nm and an emission wavelength of 610 nm, and data were then normalized to the averaged values of the control group, and the results presented as fold change.

### Detection of Ferroptosis‐Related Factors

The tissue samples or cell samples were weighed accurately and lysed and centrifuged, and the contents of Fe^2+^, MDA (Malondialdehyde), 4‐HNE (4‐Hydroxynonenal) and GSH (glutathione) / GSSG (oxidized glutathione) were detected according to the manufacturer's manuals of ferrous iron colorimetric assay kit (Elabscience, E‐BC‐K773‐M/E‐BC‐K881‐M, China), MDA colorimetric assay kit (Elabscience, E‐BC‐K025‐S, China), 4‐HNE ELISA kit (Elabscience, E‐EL‐0128, China) and GSH/GSSG colorimetric assay kit (Elabscience, E‐BC‐K097‐M, China).

### Quantitative RT‐PCR Assay

Total RNA was isolated from human astrocytes using the RNAiso Plus (9108Q, TaKaRa, Japan) and quantified by Nano Drop 2000 (Thermo, USA). Reverse transcription was done using the All‐in‐One cDNA Synthesis Super Mix (B24403, Bimake, USA). Quantitative PCR was performed using the ABI 7500 Real‐Time PCR System with the ChamQ Universal SYBR qPCR MasterMix (Q711, Vazyme, China). The primer sequences for qPCR are listed in Table s4 (Supporting Information).

### Palmitoylation of TfR1 Assays—Click Chemistry Reaction (CCR) – Based Method

Initially, cells were metabolically labeled with a chemical palmitic acid probe, alkylene palmitic acid (Alkyl‐C16; HY‐W040304, MedChemExpress, USA) for 24 h.

For immunofluorescence analysis, the cells were fixed in 4% paraformaldehyde in PBS for 10 min, and then subjected to click chemistry using Click‐iT Cell Reaction Buffer Kit (C10276, Invitrogen, USA) to conjugate Alexa Fluor 594‐azide (A10270, Invitrogen, USA) to Alkyl‐C16 for visualizing the palmitoylated proteins. Subsequently, cells were subjected to immunostaining to label TfR1, Na^+^‐K^+^‐ATPase, and Hoechst. Eventually, the images were captured by a confocal microscope (LSM 710, Carl Zeiss Co. Ltd., Germany, 63 × oil immersion objective, pixel sizes: 2048 × 2048).

For Western blots, cells were lysed and then divided into two groups. One group was subjected to click chemistry using Click‐iT Protein Reaction Buffer Kit (Thermo Fisher Scientific, C10276) to conjugate Alkyl‐C16 to Azide magnetic bead (PuriMag G Series, PuriMag Biotech, China) to pull down all palmitoylated protein, and then was probed with an anti‐TfR1 antibody to identify the palmitoylated TfR1. Another one was directly detected for total TfR1 protein level by Western blots without CCR. Finally, the data were expressed as palmitoylated TfR1/total TfR1 to assess the level of palmitoylated TfR1.

### Palmitoylation of TfR1 Assays—Co‐Immunoprecipitation and Acyl‐Biotin Exchange Assay (IP‐ABE assay)

The ABE assay was comprised of three biochemical steps, as described previously.^[^ ^84^ ^]^ Unmodified cysteine thiols were blocked with N‐ethylmaliemide (NEM); specific cleavage and unmasking of the palmitoylated cysteine's thiol group by hydroxylamine (NH_2_OH, HAM); 3) selective labeling of the palmitoylated cysteine using a thiol‐reactive biotinylation reagent (BMCC‐biotin in the experiments). Briefly, cells were lysed in lysis buffer (LB: 50 mm Tris‐HCl pH7.5, 150 mm NaCl, 10% Glycerol, 1% IGEPAL CA‐630) containing 50 mm NEM (Sigma, E3876), and then TfR1 was immunoprecipitated from the lysates on magnetic beads, which bind to anti‐TfR1 antibody. Subsequently, split each sample of beads into two samples, one omitting the HAM cleavage step (‐HAM), and one including the HAM step (+HAM), which was treated with1 M HAM. Next, add 2 µm Biotin‐BMCC (Thermo Scientific, #21900) to each sample for selective labeling of the newly exposed cyateinyl thiols after HAM cleavage. Finally, the beads were washed and immunoblotted with IRDye 800CW Streptavidin (Licor bio, 926–32230) to detect palmitoylated TfR1 (Biotin‐TfR1).

### Medium‐ and Long‐Chain Fatty Acid Measurements in Astrocytes

Medium‐ and long‐chain fatty acid measurements were performed by Shanghai Applied Protein Technology Co., Ltd. (Shanghai, China). The serum samples were added into chloroform methanol (2:1 v/v). After ultrasonication for 30 min, 2 mL of 1% sulfuric acid in methanol was added to the supernatant. Fatty acids in the mixture were esterified to methyl esterification and subjected to GS‐MS using an Agilent Model 7890A/5975C GC‐MS system. Supelco 37‐component FAME (fatty‐acid methyl ester) mix (Sigma–Aldrich) was used to construct a calibration curve for the concentration range of 0.5–1000 mg L^−1^. The internal standard was used to correct for injection variability between samples and minor changes in the instrument response.

The samples were separated with an Agilent DB‐WAX capillary GC column (30 m × 0.25 mm ID × 0.25 µm). The initial temperature was 50 °C and remained as such for 3 min. The temperature was then increased to 220 °C at 10 °C min^−1^, and remained at 220 °C for 20 min. The carrier gas was helium (1.0 mL min^−1^). A quality‐control sample was used for testing and evaluating the stability and repeatability of the system. The temperatures of the injection port and transmission line were 280 and 250 °C, respectively. The electron bombardment ionization source, SIM (Selected ion Monitor) scanning mode, and electron energy were 70 eV.

### Statistical Analysis

No data exclusion was performed for statistical analysis. Data and statistical analyses were performed using GraphPad Prism (version 10). The data were presented as Mean ± SD. Sample size (n) for each statistical analysis was indicated in each figure legends. Statistical analysis for multiple comparisons was performed by one‐way analysis of variance (ANOVA) or two‐way ANOVA with a post hoc Tukey's test, and the difference between two groups was evaluated by unpaired Student's *t* test. Nonparametric data, such as neurological deficit scores, were evaluated by one‐way ANOVA with a post hoc Kruskal‐Wallis test, or multiple *t* tests whose statistical significance was determined using the Mann‐Whitney test. *p* < 0.05 was considered statistically significant. ^*^ *p* < 0.05, ^**^ *p* < 0.01, ^***^ *p* < 0.001, ^****^ *p* < 0.0001, ns: no significance.

## Conflict of Interest

The authors declare no conflict of interest.

## Supporting information



Supporting Information

Supporting Information

## Data Availability

Raw sequencing data in this study are openly available in Mendeley Dataraw database, view it here: https://data.mendeley.com/datasets/drry6s46rp/1 (10.17632/drry6s46rp.1), https://data.mendeley.com/datasets/349jzcgc2g/1 (doi: 10.17632/349jzcgc2g.1) and https://data.mendeley.com/datasets/zz95j93jt8/1 (doi: 10.17632/zz95j93jt8.1). The raw data in this study is available on Mendeley Dataraw database, view it here: https://data.mendeley.com/datasets/p4sjb8mmw9/1 (doi: 10.17632/p4sjb8mmw9.1). The data that support the findings of this study are available from the corresponding author upon reasonable request.

## References

[1] V. L. Feigin , M. Brainin , B. Norrving , S. Martins , R. L. Sacco , W. Hacke , M. Fisher , J. Pandian , P. Lindsay , Int. J. Stroke. 2022, 17, 18. 34986727 10.1177/17474930211065917

[2] X. Liao , Y. Wang , Y. Pan , C. Wang , X. Zhao , D. Z. Wang , C. Wang , L. Liu , Y. Wang , Stroke 2014, 45, 2354. 25013020 10.1161/STROKEAHA.114.005989

[3] S. J. Warach , A. N. Dula , T. J. Milling , Stroke 2020, 51, 3440. 33045929 10.1161/STROKEAHA.120.029749 PMC7606819

[4] Q. Z. Tuo , S. T. Zhang , P. Lei , Med. Res. Rev. 2022, 42, 259. 33957000 10.1002/med.21817

[5] M. Xu , H. L. Zhang , Acta Pharmacol. Sin. 2011, 32, 1089. 21804578 10.1038/aps.2011.50 PMC4003297

[6] Y. Qin , Y. He , Y. M. Zhu , M. Li , Y. Ni , J. Liu , H. L. Zhang , Acta Pharmacol. Sin. 2018, 40, 724. 30315251 10.1038/s41401-018-0166-8 PMC6786391

[7] S. J. Dixon , K. M. Lemberg , M. R. Lamprecht , R. Skouta , E. M. Zaitsev , C. E. Gleason , D. N. Patel , A. J. Bauer , A. M. Cantley , W. S. Yang , B. Morrison , B. R. Stockwell , Cell 2012, 149, 1060. 22632970 10.1016/j.cell.2012.03.042 PMC3367386

[8] X. Jiang , B. R. Stockwell , M. Conrad , Nat. Rev. Mol. Cell Biol. 2021, 22, 266. 33495651 10.1038/s41580-020-00324-8 PMC8142022

[9] D. Tang , X. Chen , R. Kang , G. Kroemer , Cell Res. 2020, 31, 107. 33268902 10.1038/s41422-020-00441-1 PMC8026611

[10] Z. Shi , N. Naowarojna , Z. Pan , Y. Zou , Nat. Commun. 2021, 12, 4792. 34373463 10.1038/s41467-021-25159-5 PMC8352933

[11] S. J. Dixon , J. A. Olzmann , Nat. Rev. Mol. Cell Biol. 2024, 25, 424. 38366038 10.1038/s41580-024-00703-5 PMC12187608

[12] Y. Zhou , J. Liao , Z. Mei , X. Liu , J. Ge , Oxid. Med. Cell. Longev. 2021, 9991001. 34257829 10.1155/2021/9991001 PMC8257382

[13] M. Dodson , R. Castro‐Portuguez , D. D. Zhang , Redox Biol. 2019, 23, 101107. 30692038 10.1016/j.redox.2019.101107 PMC6859567

[14] D. Shin , E. H. Kim , J. Lee , J. L. Roh , Biol. Med. 2018, 129, 454. 10.1016/j.freeradbiomed.2018.10.426 30339884

[15] H. Dong , Y. Xia , S. Jin , C. Xue , Y. Wang , R. Hu , H. Jiang , Cell Death Dis. 2021, 12, 1027. 34716298 10.1038/s41419-021-04307-1 PMC8556385

[16] J. Guo , Q. Z. Tuo , P. Lei , J. Neurochem. 2023, 165, 487. 36908209 10.1111/jnc.15807

[17] K. Yang , L. Zeng , X. Yuan , S. Wang , A. Ge , H. Xu , J. Zeng , J. Ge , Biomed. Pharmacother. 2022, 154, 113611. 36081288 10.1016/j.biopha.2022.113611

[18] X. Tian , X. Li , M. Pan , L. Z. Yang , Y. Li , W. Fang , Cell. Mol. Neurobiol. 2024, 44, 25. 38393376 10.1007/s10571-024-01457-6 PMC10891262

[19] S. Zhang , W. Xin , G. J. Anderson , R. Li , L. Gao , S. Chen , J. Zhao , S. Liu , Cell Death Dis. 2022, 13, 40. 35013137 10.1038/s41419-021-04490-1 PMC8748693

[20] Z. Feng , L. Min , H. Chen , W. Deng , M. Tan , H. Liu , J. Hou , Redox Biol. 2021, 43, 101984. 33933882 10.1016/j.redox.2021.101984 PMC8105676

[21] S. Levi , M. Ripamonti , A. S. Moro , A. Cozzi , Mol. Psychiatry. 2024, 29, 1139. 38212377 10.1038/s41380-023-02399-z PMC11176077

[22] A. Reinert , M. Morawski , J. Seeger , T. Arendt , T. Reinert , BMC Neurosci 2019, 20, 25. 31142282 10.1186/s12868-019-0507-7 PMC6542065

[23] A. Mukherjee , S. Dev , E. Ghosh , S. Asthana , C. K. Mukhopadhyay , The Biology of Glial Cells, Recent Advances 2022, 10, 387.

[24] L. Yang , M. Fan , F. Du , Q. Gong , Z. G. Bi , Z. J. Zhu , L. L. Zhu , Y. Ke , Biochim. Biophys. Acta. Mol. Basis Dis. 2012, 1822, 500. 10.1016/j.bbadis.2011.12.004 22198321

[25] X. Xiao , G. A. Moschetta , Y. Xu , A. L. Fisher , V. M. Alfaro‐Magallanes , S. Dev , C.‐Y. Wang , J. L. Babitt , Blood 2023, 141, 422. 36322932 10.1182/blood.2022017811 PMC9936306

[26] L. C. Montemiglio , C. Testi , P. Ceci , E. Falvo , M. Pitea , C. Savino , A. Arcovito , G. Peruzzi , P. Baiocco , F. Mancia , A. Boffi , A. des Georges , B. Vallone , Nature Commun. 2019, 10, 1121. 30850661 10.1038/s41467-019-09098-w PMC6408514

[27] M. U. Muckenthaler , S. Rivella , M. W. Hentze , B. Galy , Cell. 2017, 168, 344. 28129536 10.1016/j.cell.2016.12.034 PMC5706455

[28] W. Ci , W. Li , Y. Ke , Z.‐M. Qian , X. Shen , Cell Calcium 2003, 33, 257. 12618146 10.1016/s0143-4160(02)00240-3

[29] M. Qu , X. Zhou , X. Wang , H. Li , Int. J. Biol. Sci. 2021, 17, 4223. 34803494 10.7150/ijbs.64046 PMC8579454

[30] A. Drecourt , J. Babdor , M. Dussiot , F. Petit , N. Goudin , M. Garfa‐Traore , F. Habarou , C. Bole‐Feysot , P. Nitschke , C. Ottolenghi , M. D. Metodiev , V. Serre , I. Desguerre , N. Boddaert , O. Hermine , A. Munnich , A. Rotig , Am. J. Hum. Genet. 2018, 102, 266. 29395073 10.1016/j.ajhg.2018.01.003 PMC5985451

[31] F. Petit , A. Drecourt , M. Dussiot , C. Zangarelli , O. Hermine , A. Munnich , A. Rotig , Blood. 2021, 137, 2090. 33529321 10.1182/blood.2020006987

[32] M. Ou , Y. Jiang , Y. Ji , Q. Zhou , Z. Du , H. Zhu , Z. Zhou , Mol. Metab. 2022, 61, 101502. 35447365 10.1016/j.molmet.2022.101502 PMC9170779

[33] Q. Z. Tuo , P. Lei , K. A. Jackman , X. l. Li , H. Xiong , X. L. Li , Z. Y. Liuyang , L. Roisman , S. t. Zhang , S. Ayton , Q. Wang , P. J. Crouch , K. Ganio , X. c. Wang , L. Pei , P. A. Adlard , Y. m. Lu , R. Cappai , J. z. Wang , R. Liu , A. I. Bush , Mol. Metab. 2017, 22, 1520. 10.1038/mp.2017.171 28886009

[34] W. S. Yang , R. SriRamaratnam , M. E. Welsch , K. Shimada , R. Skouta , V. S. Viswanathan , J. H. Cheah , P. A. Clemons , A. F. Shamji , C. B. Clish , L. M. Brown , A. W. Girotti , V. W. Cornish , S. L. Schreiber , B. R. Stockwell , Cell 2014, 156, 317. 24439385 10.1016/j.cell.2013.12.010 PMC4076414

[35] A. Singh , S. Venkannagari , K. H. Oh , Y. Q. Zhang , J. M. Rohde , L. Liu , S. Nimmagadda , K. Sudini , K. R. Brimacombe , S. Gajghate , J. Ma , A. Wang , X. Xu , S. A. Shahane , M. Xia , J. Woo , G. A. Mensah , Z. Wang , M. Ferrer , E. Gabrielson , Z. Li , F. Rastinejad , M. Shen , M. B. Boxer , S. Biswal , ACS Chem. Biol. 2016, 11, 3214. 27552339 10.1021/acschembio.6b00651 PMC5367156

[36] M. Zille , J. A. Oses‐Prieto , S. R. Savage , S. S. Karuppagounder , Y. Chen , A. Kumar , J. H. Morris , K. A. Scheidt , A. L. Burlingame , R. R. Ratan , J. Neurosci. 2022, 42, 2065. 34987108 10.1523/JNEUROSCI.0923-20.2021 PMC8916756

[37] W. Guan , M. Xia , M. Ji , B. Chen , S. Li , M. Zhang , S. Liang , B. Chen , W. Gong , C. Dong , G. Wen , X. Zhan , D. Zhang , X. Li , Y. Zhou , D. Guan , A. Verkhratsky , B. Li , Commun. Biol. 2021, 4, 525. 33953326 10.1038/s42003-021-02060-x PMC8100120

[38] A. M. Ernst , S. A. Syed , O. Zaki , F. Bottanelli , H. Zheng , M. Hacke , Z. Xi , F. Rivera‐Molina , M. Graham , A. A. Rebane , P. Bjorkholm , D. Baddeley , D. Toomre , F. Pincet , J. E. Rothman , Dev. Cell. 2018, 47, 479. 30458139 10.1016/j.devcel.2018.10.024 PMC6251505

[39] E. Alvarez , N. Gironès , R. J. Davis , J. Biol. Chem. 1990, 265, 16644. 2398066

[40] H. Cao , B. Schroeder , J. Chen , M. B. Schott , M. A. McNiven , J. Biol. Chem. 2016, 291, 16424. 27226592 10.1074/jbc.M116.724997 PMC4974358

[41] X. X. Duan , G. P. Zhang , X. B. Wang , H. Yu , J. L. Wu , K. Z. Liu , L. Wang , X. Long , Mol. Neurobiol. 2016, 54, 1677. 26873852 10.1007/s12035-016-9756-y

[42] N. Badjatia , D. Seres , A. Carpenter , J. M. Schmidt , K. Lee , S. A. Mayer , J. Claassen , E. S. Connolly , M. S. Elkind , Stroke. 2012, 43, 691. 22282893 10.1161/STROKEAHA.111.636035 PMC4198386

[43] X. Ge , Z. He , C. Cao , T. Xue , J. Jing , R. Ma , W. Zhao , L. Liu , K. Jueraitetibaike , J. Ma , Y. Feng , Z. Qian , Z. Zou , L. Chen , C. Fu , N. Song , B. Yao , Redox Biol. 2022, 54, 102380. 35803125 10.1016/j.redox.2022.102380 PMC9287734

[44] G. Carta , E. Murru , S. Banni , C. Manca , Front. Physiol. 2017, 8, 902. 29167646 10.3389/fphys.2017.00902 PMC5682332

[45] J. N. Jernberg , C. E. Bowman , M. J. Wolfgang , S. Scafidi , J. Neurochem. 2017, 142, 407. 28512781 10.1111/jnc.14072 PMC5927624

[46] G. Miotto , M. Rossetto , M. L. Di Paolo , L. Orian , R. Venerando , A. Roveri , A. M. Vuckovic , V. Bosello Travain , M. Zaccarin , L. Zennaro , M. Maiorino , S. Toppo , F. Ursini , G. Cozza , Redox Biol. 2020, 28, 101328. 31574461 10.1016/j.redox.2019.101328 PMC6812032

[47] R. C. Hider , D. L. Longo , A. V. Hoffbrand , N. Engl. J. Med. 2018, 379, 2140. 30485781 10.1056/NEJMra1800219

[48] C. B. Billesbølle , C. M. Azumaya , R. C. Kretsch , A. S. Powers , S. Gonen , S. Schneider , T. Arvedson , R. O. Dror , Y. Cheng , A. Manglik , Nature. 2020, 586, 807. 32814342 10.1038/s41586-020-2668-z PMC7906036

[49] P. Zhang , S. Wang , L. Wang , B. C. Shan , H. Zhang , F. Yang , Z. Q. Zhou , X. Wang , Y. Yuan , Y. J. Xu , J. Mol. Endocrinol. 2018, 60, 299. 10.1530/JME-17-0301 29563156

[50] N. E. Savaskan , A. Borchert , A. U. Brauer , H. Kuhn , Biol. Med. 2007, 43, 191. 10.1016/j.freeradbiomed.2007.03.033 17603929

[51] L. Sprimont , P. Janssen , K. De Swert , M. Van Bulck , I. Rooman , J. Gilloteaux , A. Massie , C. Nicaise , Sci. Rep. 2021, 11, 12227. 34108554 10.1038/s41598-021-91698-y PMC8190126

[52] D. Gong , M. Chen , Y. Wang , J. Shi , Y. Hou , Cell Death Discov. 2022, 8, 427. 36289191 10.1038/s41420-022-01218-8 PMC9605952

[53] B. F. Roeck , S. Lotfipour Nasudivar , M. R. H. Vorndran , L. Schueller , F. I. Yapici , M. Rubsam , S. von Karstedt , C. M. Niessen , A. J. Garcia‐Saez , Nat. Commun. 2025, 16, 2951. 40140422 10.1038/s41467-025-58175-w PMC11947162

[54] S. V. Narayanan , K. R. Dave , M. A. Perez‐Pinzon , Transl. Stroke Res. 2018, 9, 99. 29103101 10.1007/s12975-017-0574-y PMC6771255

[55] J. R. Liddell , Antioxidants 2017, 6, 65.28820437

[56] X. X. Deng , S. S. Li , F. Y. Sun , Aging Dis. 2019, 10, 807. 31440386 10.14336/AD.2018.0728 PMC6675533

[57] Y. M. Zhu , L. Lin , C. Wei , Y. Guo , Y. Qin , Z. S. Li , T. A. Kent , C. E. McCoy , Z. X. Wang , Y. Ni , X. Y. Zhou , H. L. Zhang , Transl. Stroke Res. 2021, 12, 991. 33629276 10.1007/s12975-021-00888-3 PMC8557200

[58] B. R. Broughton , D. C. Reutens , C. G. Sobey , Stroke 2009, 40, 331. 10.1161/STROKEAHA.108.531632 19182083

[59] T. Qiu , X. Yang , J. Wang , C. Pan , X. Chu , J. Xiong , J. Xie , Y. Chang , C. Wang , J. Zhang , Nutr. Diabetes 2022, 12, 23. 35443706 10.1038/s41387-022-00202-6 PMC9021212

[60] J. Korbecki , K. Bajdak‐Rusinek , Inflamm. Res. 2019, 68, 915. 31363792 10.1007/s00011-019-01273-5 PMC6813288

[61] X. Chen , K. Chen , J. Hu , Y. Dong , M. Zheng , J. Jiang , Q. Hu , W. Zhang , Commun. Biol. 2024, 7, 539. 38714886 10.1038/s42003-024-06248-9 PMC11076507

[62] H. Liu , B. Su , Z. Zhang , S. Jia , J. Wang , F. Zhou , Y. Liu , Q. Cao , J. Tang , Z. Ou , M. M. Zhang , Y. Chen , H. Dong , H. Zhong , J. Adv. Res. 2025, 8, S2090. 10.1016/j.jare.2025.02.009 PMC12684956 39929269

[63] D. Xue , X. Zhou , J. Qiu , Biomed. Pharmacother. 2020, 131, 110676. 32858502 10.1016/j.biopha.2020.110676

[64] X. L. Chen , G. P. Zhang , S. L. Guo , J. Q. Ding , J. J. Lin , Q. Yang , Z. Y. Li , J. Mol. Neurosci. 2017, 63, 267. 28952074 10.1007/s12031-017-0976-z

[65] F. Kielar , M. E. Helsel , Q. Wang , K. J. Franz , Metallomics 2012, 4, 899. 22700084 10.1039/c2mt20069d PMC3427476

[66] L. Liao , C. Jiang , J. Chen , J. Shi , X. Li , Y. Wang , J. Wen , S. Zhou , J. Liang , Y. Lao , J. Zhang , Eur. J. Med. Chem. 2020, 190, 112114. 32061962 10.1016/j.ejmech.2020.112114

[67] R. Guo , Y. Zhao , Y. Li , T. Wang , Y. Li , B. Sutton , Z. P. Liang , Magn. Reson. Med. 2021, 85, 970. 32810319 10.1002/mrm.28459 PMC7722130

[68] F. Lam , Z. P. Liang , Magn. Reson. Med. 2014, 71, 1349. 24496655 10.1002/mrm.25168 PMC4051394

[69] F. Lam , C. Ma , B. Clifford , C. L. Johnson , Z. P. Liang , Magn. Reson. Med. 2016, 76, 1059. 26509928 10.1002/mrm.26019 PMC4848237

[70] R. Guo , Y. Zhao , Y. Li , Y. Li , Z.‐P. Liang , Magn. Reson. Med 2019, 82, 1993. 31294487 10.1002/mrm.27865 PMC6717045

[71] C. Ma , F. Lam , C. L. Johnson , Z. P. Liang , Magn. Reson. Med. 2016, 75, 488. 25762370 10.1002/mrm.25635 PMC4567537

[72] Z. P. Liang , in 2007 4th IEEE International Symposium on Biomedical Imaging, From Nano to Macro – Proceedings , IEEE, Arlington VA USA 2007.

[73] F. Lam , Y. Li , R. Guo , B. Clifford , Z. P. Liang , Magn Reson Med 2020, 83, 377. 31483526 10.1002/mrm.27980 PMC6824949

[74] Y. Li , F. Lam , B. Clifford , Z.‐P. Liang , IEEE Trans. Biomed. Eng. 2017, 64, 2486. 28829303 10.1109/TBME.2017.2741922 PMC5646283

[75] L. Vanhamme , A. Van Den Boogaart , S. Van Huffel , J. Magn. Reson. 1997, 129, 35. 9405214 10.1006/jmre.1997.1244

[76] D. Zhou , T. Liu , P. Spincemaille , Y. Wang , NMR Biomed. 2014, 27, 312. 24395595 10.1002/nbm.3064

[77] J. Liu , T. Liu , L. De Rochefort , J. Ledoux , I. Khalidov , W. Chen , A. J. Tsiouris , C. Wisnieff , P. Spincemaille , M. R. Prince , Y. Wang , Neuroimage 2012, 59, 2560. 21925276 10.1016/j.neuroimage.2011.08.082 PMC3254812

[78] X. X. Wang , F. Wang , G. H. Mao , J. C. Wu , M. Li , R. Han , J. She , R. Zhang , R. Sheng , Z. Chen , Z. H. Qin , Acta Pharmacol. Sin. 2021, 43, 529. 34168317 10.1038/s41401-021-00705-5 PMC8888674

[79] Y. M. Zhu , X. Gao , Y. Ni , W. Li , T. A. Kent , S. G. Qiao , C. Wang , X. X. Xu , H. L. Zhang , Neuroscience 2017, 356, 125. 28501505 10.1016/j.neuroscience.2017.05.004

[80] L. Z. Hong , W. W. Gu , Y. Ni , M. Xu , L. Yang , Y.‐L. Liu , S. L. Yang , Q. Zhou , X. M. Gao , H. L. Zhang , Evid. Based Complementary Altern. Med. 2013, 2013, 795365. 10.1155/2013/795365 PMC3830819 24319485

[81] V. Bouet , M. Boulouard , J. Toutain , D. Divoux , M. Bernaudin , P. Schumann‐Bard , T. Freret , Nat. Protoc. 2009, 4, 1560. 19798088 10.1038/nprot.2009.125

[82] H. P. Du , Y. Guo , Y. M. Zhu , D. F. Gao , B. Lin , Y. Liu , Y. Xu , A. Said , T. Khan , L. J. Liu , J. J. Zhu , Y. Ni , H. L. Zhang , Acta Pharmacol. Sin. 2023, 44, 1549. 37055533 10.1038/s41401-023-01069-8 PMC10374908

[83] J. J. Guo , F. Yue , D. Y. Song , L. Bousset , X. Liang , J. Tang , L. Yuan , W. Li , R. Melki , Y. Tang , P. Chan , C. Guo , J. Y. Li , Cell Death Dis. 2021, 12, 81. 33441545 10.1038/s41419-020-03369-x PMC7807015

[84] S. X. Bamji , G. S. Brigidi , JoVE 2013, 18, 50031. 10.3791/50031 PMC3605617 23438969

