# Supplementary material for: Iron Overload Mediates the Differential Cell Fate of Astrocytes from Neurons and Its Regulatory Mechanisms in Ischemic Stroke
Source: Adv Sci (Weinh). 2025 Nov 6;13(4):e07384. doi: 10.1002/advs.202507384 (PMC12822406; doi:10.1002/advs.202507384)
Supplement: Supplementary file 1 — Supporting Information [file ADVS-13-e07384-s002.pdf]

Supporting Information

Iron Overload Mediates the Differential Cell Fate of Astrocytes from Neurons and its Regulatory Mechanisms in Ischemic Stroke

Yi Guo, Yue Wang, Yong Ni, Bin Bo, Jinzhi He, Yongming Zhu, Aiping Qin, Xianyong Zhou, Huaping Du, Yuan Liu, Tianyao Wang, Yudu Li, Yibo Zhao, Zengai Chen, Zhipei Liang, Yao Li, Yuan Xu* and Huiling Zhang**

Supporting Information includes:
Figures S1-S18
Tables S1 to 4

Other supporting information includes:
Data S1

Supplementary Figures:

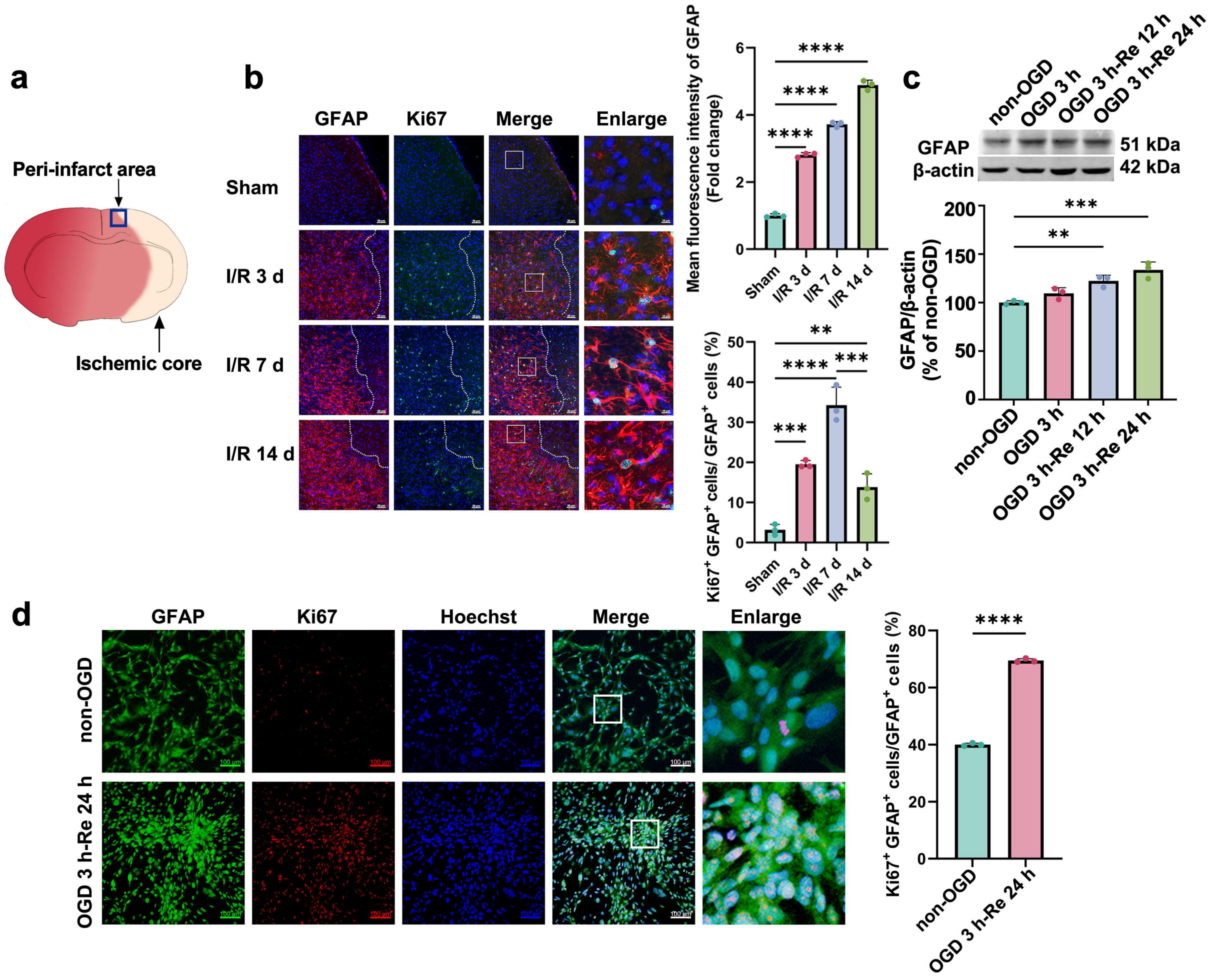

Figure S1. Reactive proliferation of astrocytes after ischemic stroke. (**a**) The sampling location of mouse brain tissue and the site for histochemical observation. Red region: non-infarcted brain, white region: ischemic core. Blue rectangle delineates the peri-infarct area. (**b**) Immunostaining of GFAP (red) and Ki67 (green) in mice peri-infarct area at d 3, 7 and 14 post-I/R. Hoechst: blue. The GFAP fluorescence intensity and ratio of $Ki67^+$ $GFAP^+$ cells/$GFAP^+$ cells were analyzed. Scale bars, 50 µm or 10 µm. Mean ± SD, n = 3 independent biological replicates. (**c**) Western blotting analysis of GFAP in OGD/Re-treated HA cells. Quantification expressed as a percentage of non-OGD group. Mean ± SD, n = 3 independent biological replicates. (**d**) The effects of OGD 3 h-Re 24 h treatment on astrogliosis in HA cells. GFAP: green, Ki67: red and Hoechst: blue. The ratio of $Ki67^+$ $GFAP^+$ cells/$GFAP^+$ cells was analyzed. Scale bar, 100 µm. Mean ± SD, n = 3 independent biological replicates. One-way ANOVA followed by a post hoc Tukey's test (**b-c**). Student's *t* test (**d**). ** $P < 0.01$, *** $P < 0.001$, **** $P < 0.0001$.

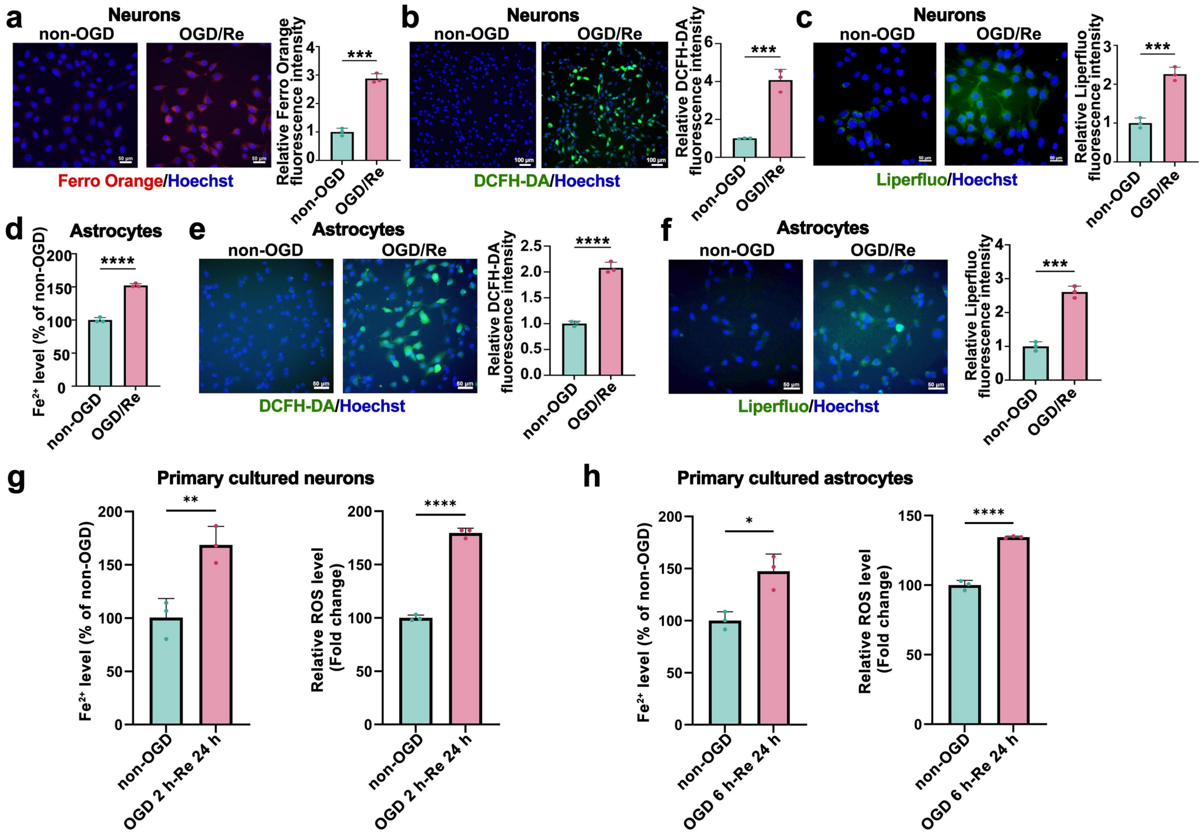

Figure S2. The increased level of pro-ferroptosis factors in neurons and astrocytes, respectively. (a-f) The levels of Fe^{2+}, ROS and lipid peroxidation in OGD 6 h-Re 24 h-treated HT22 cells (**a-c**) or OGD 3 h-Re 24 h-treated HA cells (**d-f**). The fluorescence intensity of Ferro Orange, DCFH-DA or Liperfluo was analyzed, respectively. Scale bars: 100 µm or 50 µm. Mean ± SD, n = 3 independent biological replicates. (**g**) The levels of Fe^{2+} and ROS in OGD 2 h-Re 24 h-treated primary cultured neurons. Mean ± SD, n = 3 independent biological replicates. (**h**) The levels of Fe^{2+} and ROS in OGD 6 h-Re 24 h-treated primary cultured astrocytes. Mean ± SD, n = 3 independent biological replicates. Student's *t* test. * $P < 0.05$, ** $P < 0.01$, *** $P < 0.001$, **** $P < 0.0001$.

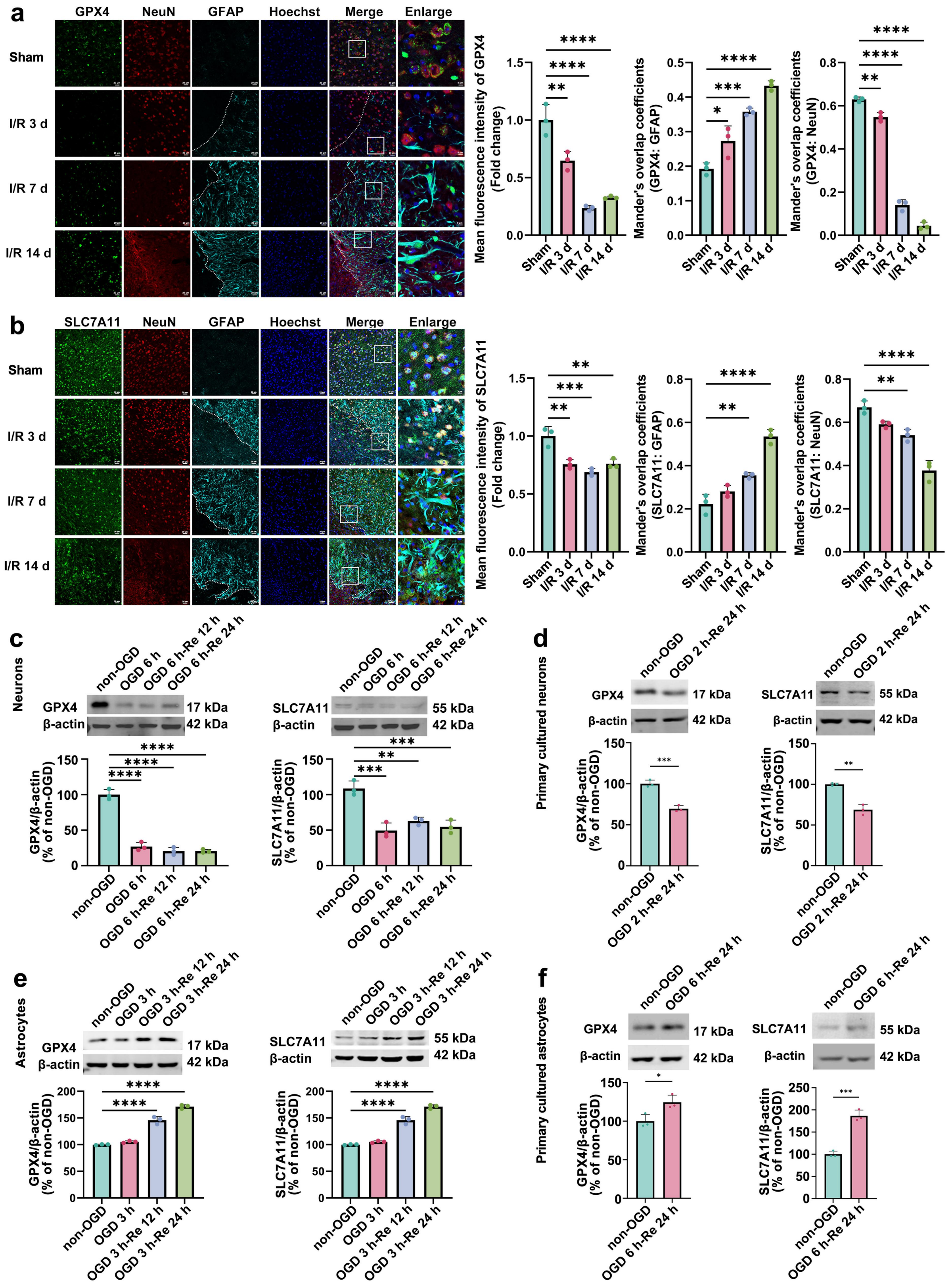

Figure S3. The expression of GPX4 or SLC7A11 in neurons and astrocytes after ischemic stroke, respectively. (**a, b**) Immunostaining showed the protein level of GPX4 (**a**) or SLC7A11 (**b**) in neurons and astrocytes at d 3, 7, 14 post-I/R in the peri-infarct area. GFAP: cyan, NeuN: red, GPX4 or SLC7A11: green and Hoechst: blue. White dotted lines, IBZ. The fluorescence intensity of GPX4 or SLC7A11 was analyzed, and the Mander's overlap coefficient was used to quantify protein colocalization. Scale bars, 20 µm or 5 µm. Mean ±

SD, n = 3 independent biological replicates. (**c, e**) Western blotting analysis of GPX4 and SLC7A11 in HT22 cells (**c**) or HA cells (**e**). Quantification expressed as a percentage of the non-OGD group. (**d, f**) Western blotting analysis of GPX4 and SLC7A11 in OGD 2 h-Re 24 h treated-primary cultured neurons (**d**) or OGD 6 h-Re 24 h treated-primary cultured astrocytes (**f**). Quantification expressed as a percentage of the control group. Mean ± SD, n = 3 independent biological replicates. One-way ANOVA followed by a post hoc Tukey's test. * P ＜0.05， ** $P < 0.01$, *** $P < 0.001$, **** $P < 0.0001$.

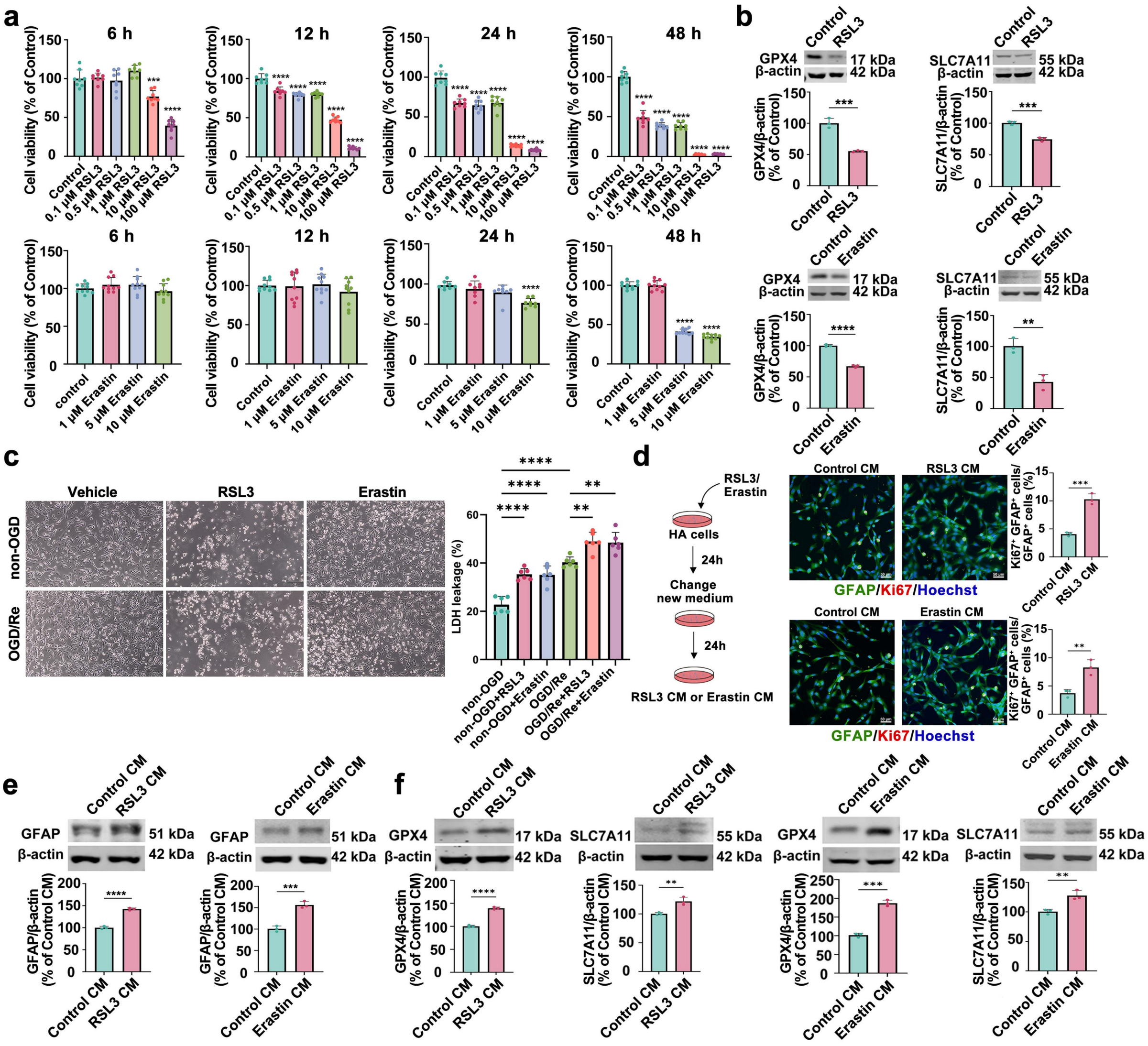

Figure S4. Effects of ferroptosis inducers RSL3 and erastin on astrocytic cell death or reactive proliferation. (**a**) HA cells were cultured with different concentrations of RSL3 or erastin for 6, 12, 24 and 48 h, and the cell viability was measured with CCK-8 assay. Mean ± SD, n = 10 independent biological replicates. **** $P < 0.0001$ vs. Control group. (**b**) Western blotting analysis of GPX4 and SLC7A11 in HA cells with 0.1 µM RSL3 or erastin 10 µM treated for 24 h. Quantification expressed as a percentage of the control group. Mean ± SD, n = 3 independent biological replicates. (**c**) Representative white light image and LDH leakage rate of HA cells treated with 0.1 µM RSL3 or 10 µM erastin during reoxygenation. Mean ± SD, n = 6 independent biological replicates. (**d-f**) HA cells were induced with RSL3 or erastin for 24 h, and the medium containing RSL3 or erastin was discarded, and the HA cells were cultured with new medium for another 24 h to obtain the conditioned medium (RSL3 CM or erastin CM). Normal cultured HA cells were cultured with conditioned medium (RSL3 CM or erastin CM) for 24 h. (**d**) The effects of RSL3 CM or erastin CM on astrogliosis in HA cells. GFAP: green, Ki67: red and Hoechst: blue. The ratio of $Ki67^+$ cells/$GFAP^+$ cells was analyzed. Scale bar, 50 µm. Mean ± SD, n = 3 independent biological replicates. (**e, f**) The expressions of GFAP (**e**), GPX4 and SLC7A11 (**f**) in HA cells with RSL3 CM or erastin CM treatment for 24 h. Mean ± SD, n = 3 independent biological replicates. One-way ANOVA followed by a post hoc Tukey's test (**a, c**). Student's *t* test (**b, d, e-f**). ** $P < 0.01$, *** $P < 0.001$, **** $P < 0.0001$.

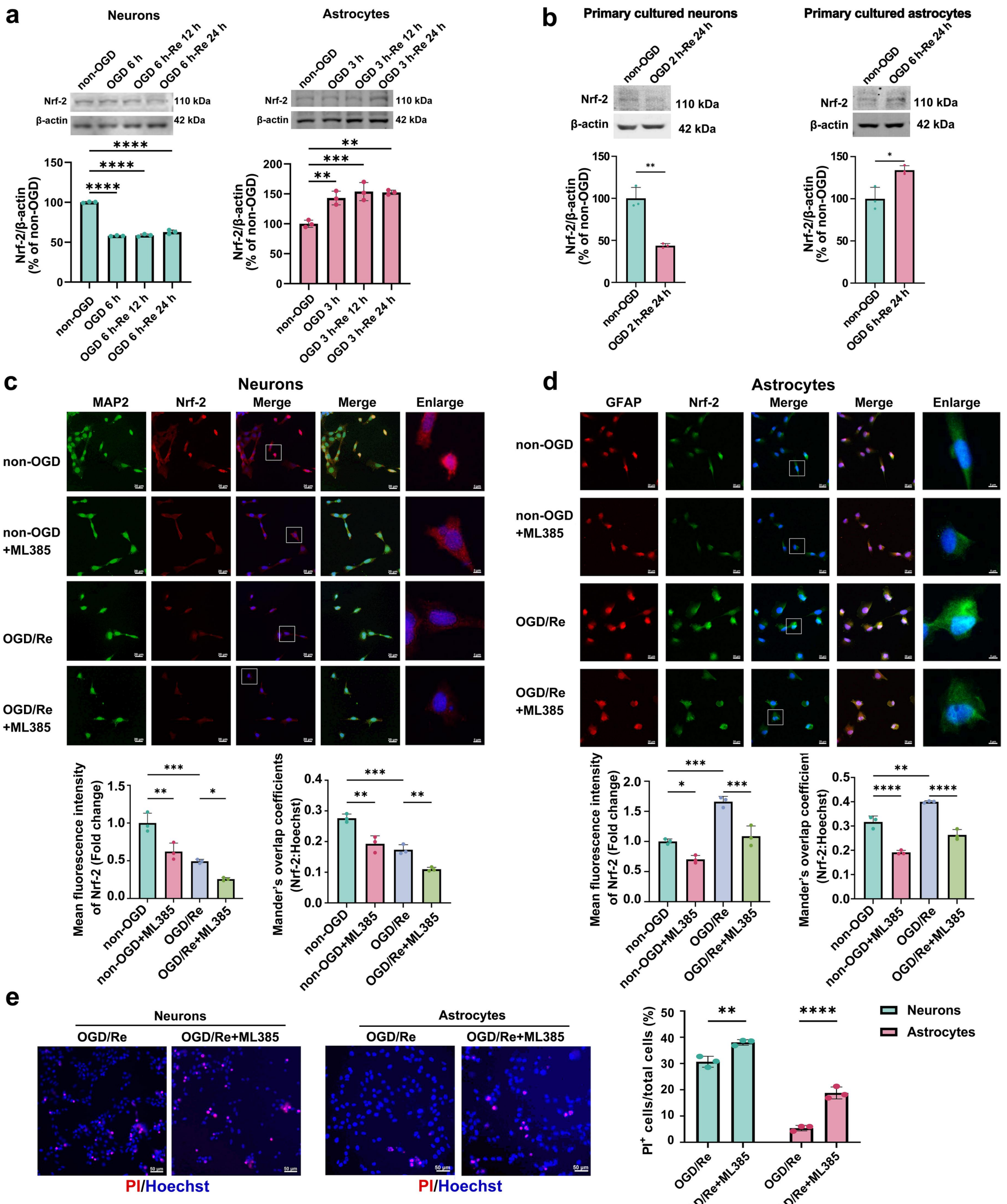

Figure S5. ML385, a specific inhibitor of Nrf-2, inhibits the expression and nuclear translocation of Nrf-2 in HT22 and HA cells, respectively. (**a-b**) Western blotting analysis of Nrf-2 in HT22 cells and HA cells (**a**) or primary cultured neurons and astrocytes (**b**) after OGD/Re treatment. Quantification expressed as a percentage of the non-OGD group. Mean ± SD, n = 3 independent biological replicates. (**c**) Immunostaining showed the expression and nuclear localization of Nrf-2 in HT22 cells. MAP2: green, Nrf-2: red and Hoechst: blue. Scale bars, 20 μm or 5 μm. The fluorescence intensity of Nrf-2 and the Mander's overlap coefficient of Nrf-2 with nucleus were analyzed. (**d**) The expression and nuclear localization of Nrf-2 in HA cells. GFAP: red, Nrf-2: green and Hoechst: blue. Scale bars, 20 μm or 5 μm. The fluorescence intensity of Nrf-2 and the Mander's overlap coefficient of Nrf-2 with nucleus were analyzed. Mean ± SD, n = 3 independent biological replicates. (**e**) PI staining (PI^+: red) was used to detect the effects of ML385 on cell death in HT22 cells or HA cells. Hoechst:

blue. The ratio of PI^+ cells/total cells was analyzed. Scale bar, 50 μm. Mean ± SD. n = 3 independent biological replicates. One-way ANOVA followed by a post hoc Tukey's test (**a, c-d**). Student's *t* test (**b, e**). ** $P < 0.01$, *** $P < 0.001$, **** $P < 0.0001$.

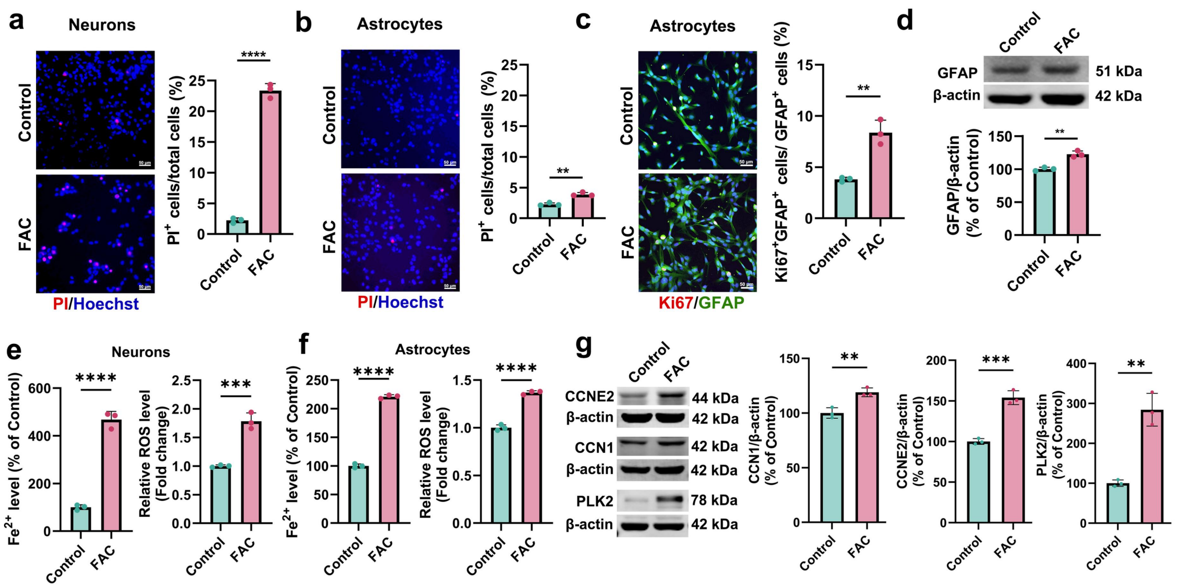

Figure S6. Iron overload mediates the neuronal death and astrocytic proliferation. (**a, b**) PI staining (PI^+: red) was used to detect the effects of 3.13 μM FAC on cell death in HT22 cells (**a**) or HA cells (**b**). Hoechst: blue. The ratio of PI^+ cells/total cells was analyzed. Scale bar, 50 μm. Mean ± SD. n = 3 independent biological replicates. (**c**) The effects of 3.13 μM FAC on astrogliosis in HA cells. GFAP: green, Ki67: red, Hoechst: blue. The ratio of $Ki67^+$ $GFAP^+$ cells/total $GFAP^+$ cells was analyzed. Scale bar, 50 μm. Mean ± SD. n = 3 independent biological replicates. (**d**) Western blotting analysis of GFAP in HA cells with 3.13 μM FAC treatment. Quantification expressed as a percentage of the control group. Mean ± SD. n = 3 independent biological replicates. (**e, f**) The levels of Fe^{2+} and ROS in HT22 or HA cells with 3.13 μM FAC treatment. Mean ± SD. n = 3 independent biological replicates. (**g**) Western blotting analysis of CCN1, CCNE2 and PLK2 in HA cells after 3.13 μM FAC treatment. Quantification expressed as a percentage of the control group. Mean ± SD. n = 3 independent biological replicates. Student's *t* test. ** $P < 0.01$, *** $P < 0.001$, **** $P < 0.0001$.

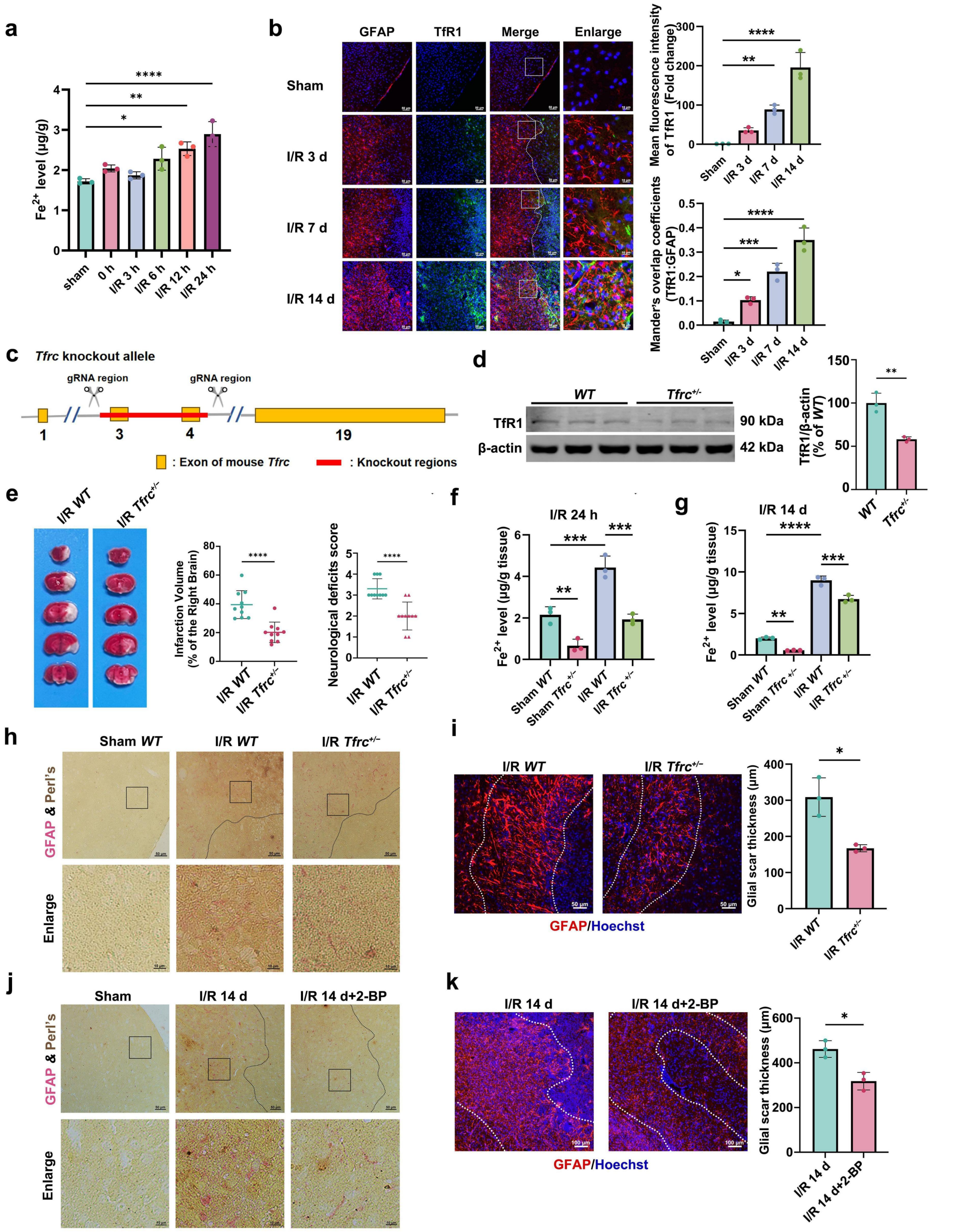

Figure S7. Elevated TfR1 and TfR1 palmitoylation promote the iron overload and glial scar formation. (**a**) Time course changes of Fe^{2+} levels in the peri-infarct area of mice after I/R. Mean ± SD, n = 3 independent biological replicates. (**b**) Immunostaining showed the protein level of TfR1 in astrocytes after I/R. GFAP: red, TfR1: green and Hoechst: blue. The fluorescence intensity of TfR1 was analyzed, and the Mander's overlap coefficient was used to measure the colocalization of TfR1 with GFAP. Scale bars, 50 μm or 10 μm. Mean ± SD, n = 3 independent biological replicates. (**c**) Schematic of transgenic strategy of *Tfrc*$^{+/-}$ mice. (**d**) Western blotting analysis of TfR1 in cerebral cortex brain tissue in *Tfrc*$^{+/-}$ mice.

Quantification expressed as a percentage of the *WT* group. Mean ± SD, n = 3 independent biological replicates. (**e**) The cerebral infarction volume and neurobehavioral deficits scores in WT and *Tfrc*$^{+/-}$ mice at 24 h after I/R. (**f, g**) The levels of Fe^{2+} in peri-infarct area tissue at d 1 (**f**) or d 14 (**g**) post-I/R. Mean ± SD, n = 10 independent biological replicates. (**h**) GFAP & Perl's staining was used to detect iron deposition in astrocytes in WT and *Tfrc*$^{+/-}$ mice after I/R 14 days. Alkaline phosphatase staining was used to visualize GFAP (astrocytes), and iron deposition was visualized as yellow, brown, or even black granular deposits. Scale bars, 50 μm or 10 μm. n = 3 independent biological replicates. (**i**) Immunostaining showed the glial scar thickness in WT and *Tfrc*$^{+/-}$ mice at d 14 after I/R. GFAP: red, Hoechst: blue. The glial scar thickness was analyzed. Scale bar, 20 μm. Mean ± SD, n = 3 independent biological replicates. (**j, k**) 2-BP (30 mg/kg), a general protein palmitoylation inhibitor, was intravenously injected (i.v.) at d 1 post-I/R, once every two days, until at d 14 post-I/R. (**j**) GFAP & Perl's staining was used to detect iron deposition in astrocytes 14 days after I/R. Alkaline phosphatase staining was used to visualize GFAP (astrocytes), while iron deposition was visualized as yellow, brown, or even black granular deposits. Scale bars, 50 μm or 10 μm. n = 3 independent biological replicates. (**k**) Immunostaining showed the glial scar thickness after I/R 14 days. GFAP: red. Scale bar, 20 μm. Quantification of the glial scar thickness. Mean ± SD, n = 3 independent biological replicates. One-way ANOVA followed by a post hoc Tukey's test (**a, b, f, g**). Student's *t* test (**d**, **e**: infarct volume, **k**, **i**), Mann-Whitney test (**e**: neurological deficits score). * $P < 0.05$, ** $P < 0.01$, *** $P < 0.001$, **** $P < 0.0001$.

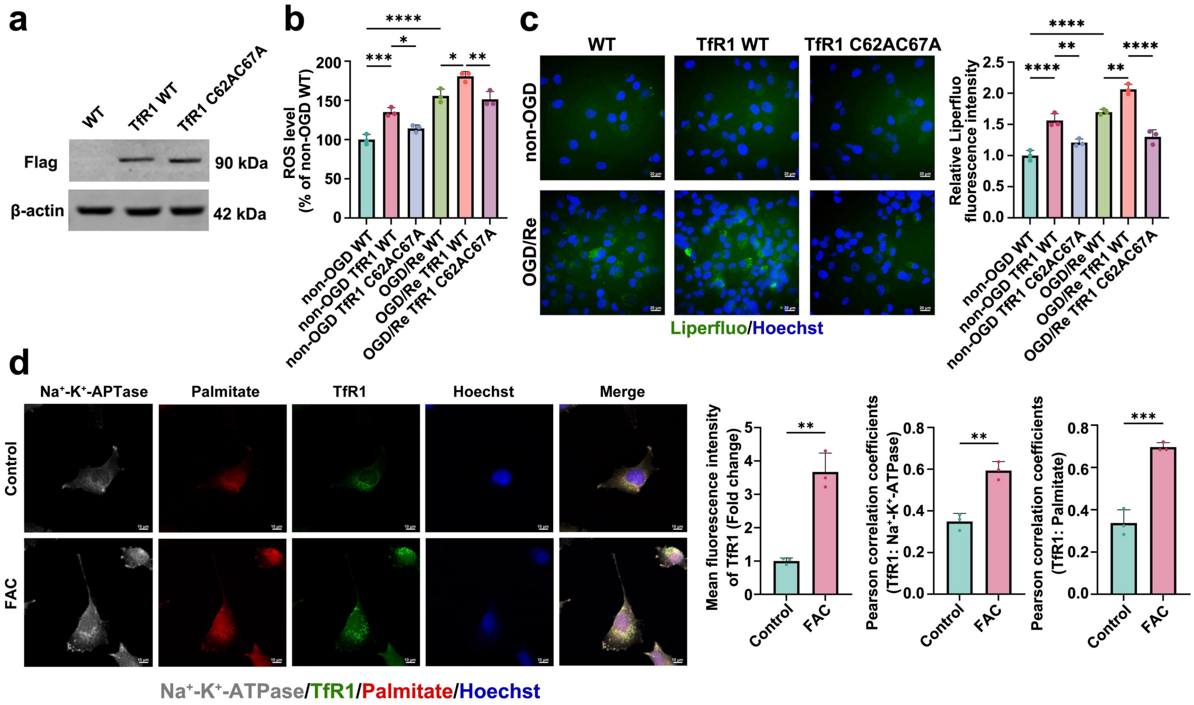

Figure S8. Inhibition of TfR1 palmitoylation decreases the pro-ferroptosis factors in astrocytes, and FAC promotes TfR1 palmitoylation in HA cell membrane. (**a**) The Flag band indicates that TfR1 WT and TfR1 C62AC67A plasmid were successfully transfected in HA cells. (**b-c**) The levels of ROS and lipid peroxidation in TfR1 WT or TfR1 C62AC67A HA cells. The fluorescence intensity of Liperfluo was analyzed. Scale bars: 20 µm. Mean ± SD, n = 3 independent biological replicates. (**d**) Click-chemistry-reaction (CCR) results showed the protein level and palmitoylation of TfR1 on the membrane in HA cells with FAC (3.13 µM, 24 h) treatment. Cell membrane (Na^+-K^+-ATPase): gray, palmitate: red, TfR1: green and Hoechst: blue. The fluorescence intensity of TfR1 was analyzed, and the Pearson correlation coefficient was used to measure the colocalization of TfR1 with Na^+-K^+-ATPase or TfR1 with palmitate. Scale bar, 10 µm. Mean ± SD, n = 3 independent biological replicates. One-way ANOVA followed by a post hoc Tukey's test (**b, c**). Student's *t* test (**d**). * $P < 0.05$, ** $P < 0.01$, *** $P < 0.001$, **** $P < 0.0001$.

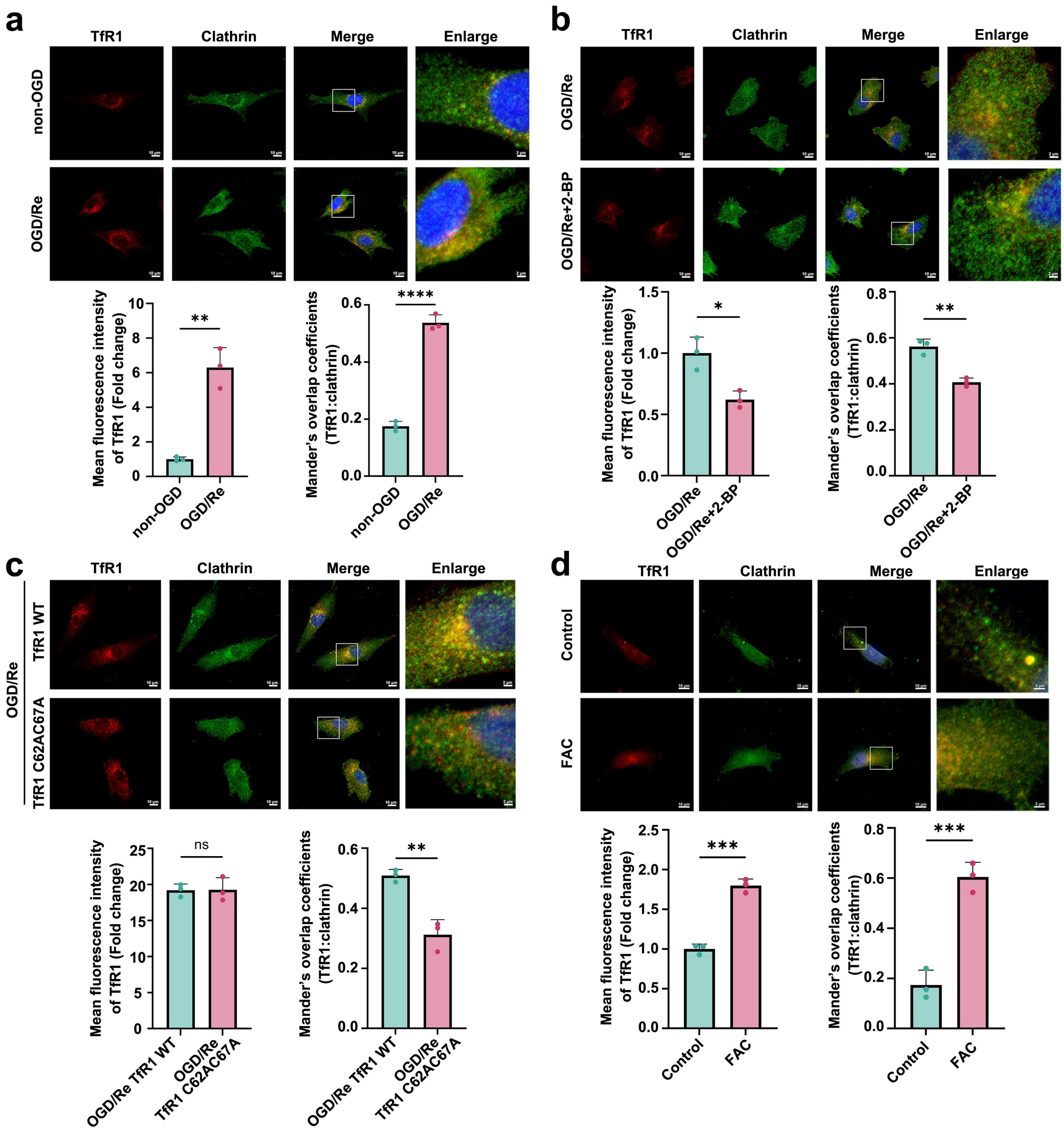

Figure S9. TfR1 palmitoylation increases the clathrin-mediated TfR1 endocytosis. (**a**) Immunostaining showed the protein level of TfR1 and the co-localization of TfR1 and clathrin in OGD/Re-treated HA cells. (**b-d**) Immunostaining showed the protein level of TfR1 and the co-localization of TfR1 and clathrin in 2-BP-treated (**b**), Cys^{62} and Cys^{67} mutant (**c**) or FAC-treated (**d**) HA cells. TfR1: red, clathrin: green and Hoechst: blue. The fluorescence intensity of TfR1 was analyzed, and the Mander's overlap coefficient was used to measure the colocalization of TfR1 with clathrin. Scale bars, 10 μm or 2 μm. Mean ± SD, n = 3 independent biological replicates. Student's *t* test. * $P < 0.05$, ** $P < 0.01$, *** $P < 0.001$, **** $P < 0.0001$.

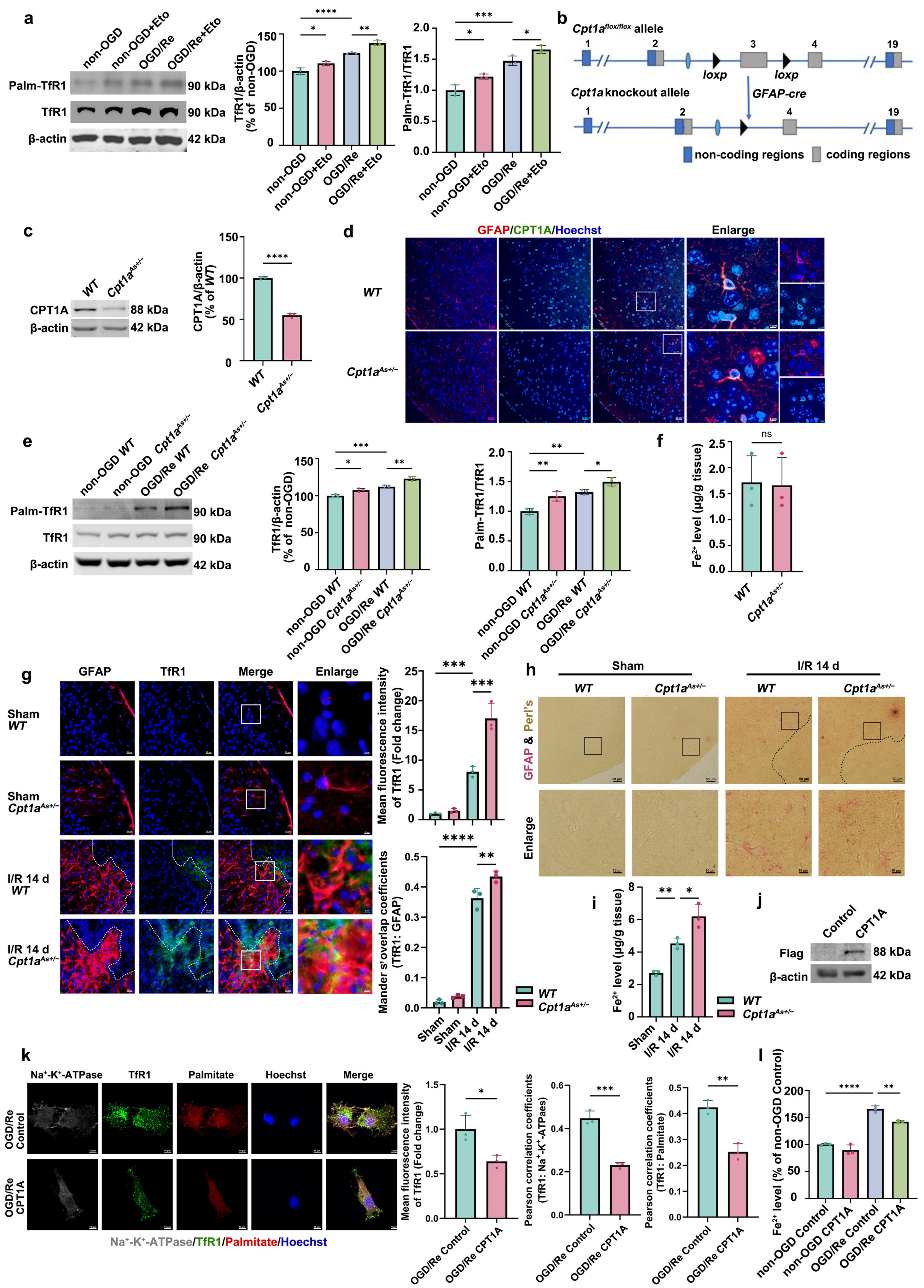

Figure S10. CPT1A dysfunction aggravates the palmitoylation of TfR1and iron overload in astrocytes. (**a**) The effects of etomoxir (Eto), an inhibitor of CPT1, on the palmitoylation of TfR1 were detected by CCR methods. Quantification expressed as a percentage of the non-OGD group and the fold change of palm-TfR1/total TfR1. Mean ± SD, n = 3 independent biological replicates. (**b**) Schematic of transgenic strategy of *Cpt1a*$^{As+/-}$ mice. (**c**) Western

blotting analysis of CPT1A in cerebral cortex in *WT* mice and $Cpt1a^{As+/-}$mice. Quantification expressed as a percentage of the *WT* group. Mean ± SD, n = 3 independent biological replicates. (**d**) Immunostaining showed the protein level of CPT1A in astrocytes of WT mice and $Cpt1a^{As+/-}$ mice. GFAP: red, CPT1A: green and Hoechst: blue. Scale bars, 20 μm or 4 μm. Mean ± SD, n = 3 independent biological replicates. (**e**) CCR results showed the protein level and palmitoylation of TfR1 in *WT* or $Cpt1a^{As+/-}$ astrocytes. Quantification expressed as a percentage of the non-OGD *WT* group and the fold change of palm-TfR1/total TfR1. Mean ± SD, n = 3 independent biological replicates. (**f, i**) The levels of Fe^{2+} in cerebral cortex of *WT* and $Cpt1a^{As+/-}$ mice. Mean ± SD, n = 3 independent biological replicates. (**g**) Immunostaining showed the level of TfR1 in astrocytes in the peri-infarct area of *WT* and $Cpt1a^{As+/-}$ mice. GFAP: red, TfR1: green and Hoechst: blue. The fluorescence intensity of TfR1 was analyzed, and the Mander's overlap coefficient was used to measure the colocalization of TfR1 with GFAP. Scale bars, 50 μm or 10 μm. Mean ± SD, n = 3 independent biological replicates. (**h**) GFAP & Perl's staining was used to detect iron deposition in astrocytes in *WT* and $Cpt1a^{As+/-}$ mice. Alkaline phosphatase staining was used to visualize GFAP (astrocytes), while iron deposition was visualized as yellow, brown, or even black granular deposits. Scale bars, 50 μm or 10 μm. n=3 independent biological replicates. (**j**) The Flag band indicates that CPT1A plasmid was successfully transfected. (**k**) CCR results showed the protein level and palmitoylation of TfR1 on the membrane in CPT1A HA cells. Na^{+}-K^{+}-ATPase: gray, palmitate: red, TfR1: green and Hoechst: blue. The fluorescence intensity of TfR1 was analyzed, and the Pearson correlation coefficient was used to measure the colocalization of TfR1 with Na^{+}-K^{+}-ATPase or TfR1 with palmitate. Scale bar, 10 μm. Mean ± SD, n = 3 independent biological replicates. (**l**) The levels of Fe^{2+} in control or CPT1A HA cells. Mean ± SD, n = 3 independent biological replicates. One-way ANOVA followed by a post hoc Tukey's test (**a, e, g, i, l**). Student's *t* test (**c, f, k**). * $P < 0.05$, ** $P < 0.01$, *** $P < 0.001$, **** $P < 0.0001$.

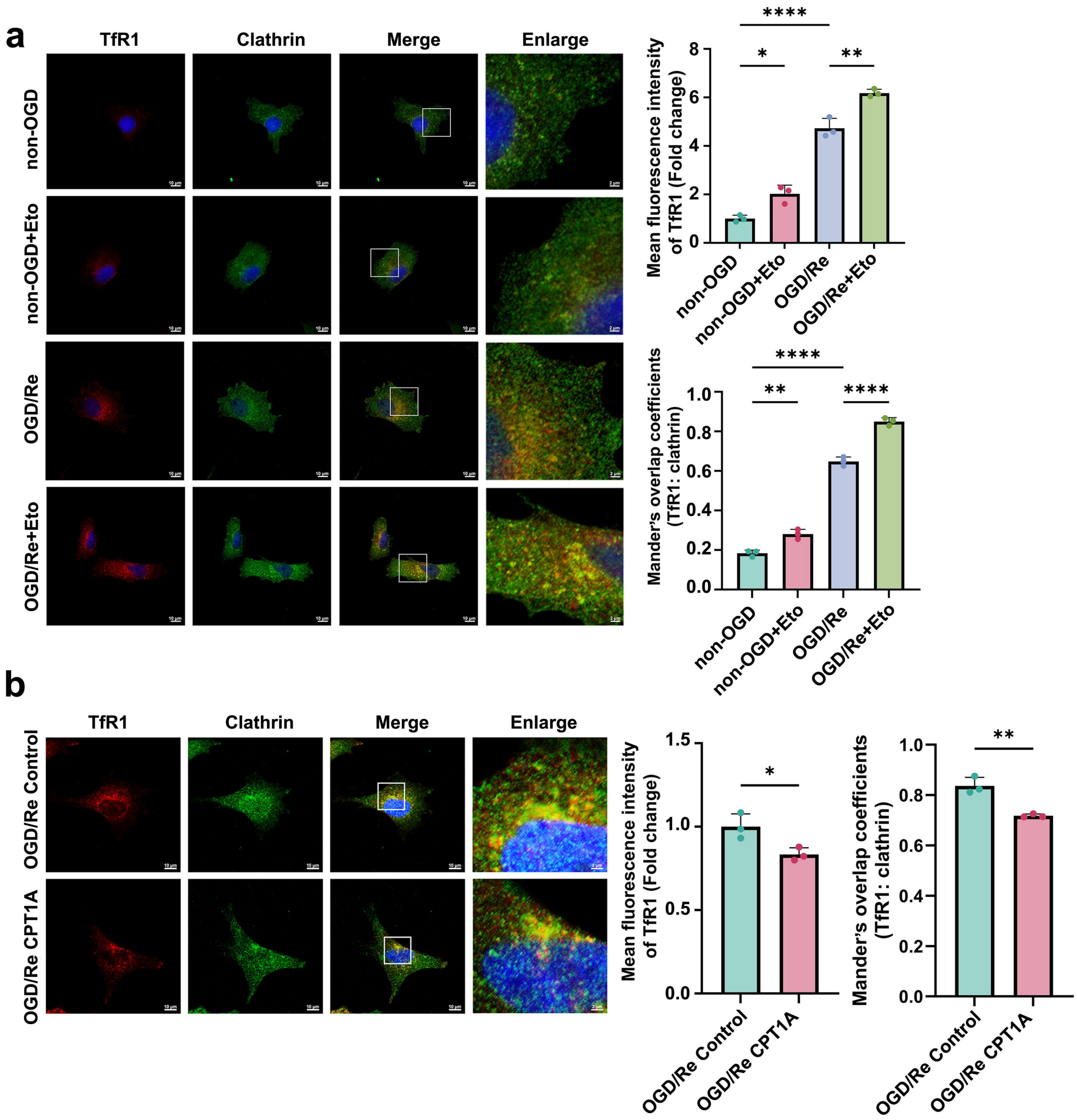

Figure S11. CPT1A dysfunction aggravates the clathrin-mediated TfR1 endocytosis. (**a**) Immunostaining showed the protein level of TfR1 and the co-localization of TfR1 and clathrin in HA cells with Eto treatment. TfR1: red, clathrin: green and Hoechst: blue. Scale bars, 10 μm or 2 μm. The fluorescence intensity of TfR1 was analyzed, and the Mander's overlap coefficient was used to measure the colocalization of TfR1 with clathrin. Mean ± SD, n = 3 independent biological replicates. (**b**) Immunostaining showed the protein level of TfR1 and the co-localization of TfR1 and clathrin in CPT1A HA cells. TfR1: red, clathrin: green and Hoechst: blue. The fluorescence intensity of TfR1 was analyzed, and the Mander's overlap coefficient was used to measure the colocalization of TfR1 with clathrin. Scale bars, 10 μm or 2 μm. Mean ± SD, n = 3 independent biological replicates. One-way ANOVA followed by a post hoc Tukey's test (**a**). Student's *t* test (**b**). * $P < 0.05$, ** $P < 0.01$, *** $P < 0.001$, **** $P < 0.0001$.

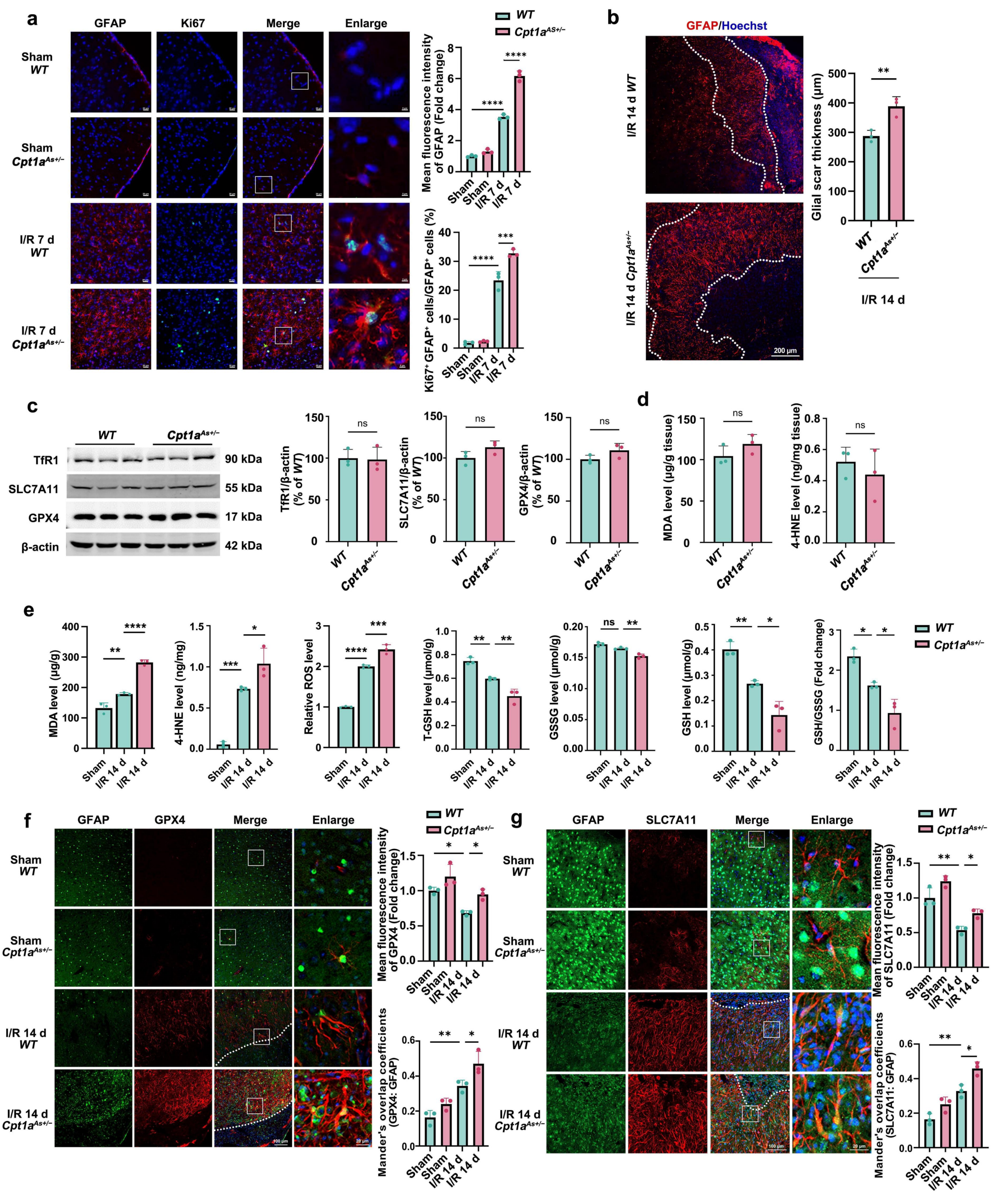

Figure S12. CPT1A dysfunction increases both pro-ferroptotic factors levels and anti-ferroptosis proteins in reactive astrocytes, promoting the glial scar formation 14 d after ischemic stroke in mice. (**a**) Immunostaining of GFAP (red) and Ki67 (green) indicated reactive proliferation of astrocytes in the peri-infarct area of *WT* and *Cpt1a*$^{As+/-}$ mice at d 14 post-I/R. Hoechst: blue. Scale bars, 50 μm or 10 μm. The GFAP fluorescence intensity and ratio of Ki67^{+} GFAP^{+} cells/total GFAP^{+} cells were analyzed. Mean ± SD, n = 3 independent biological replicates. (**b**) Immunostaining showed the glial scar thickness in *WT* and *Cpt1a*$^{As+/-}$ mice. GFAP: red and Hoechst: blue. Scale bar, 20 μm. The glial scar thickness was analyzed. Mean ± SD, n = 3 independent biological replicates. (**c**) Western blotting analysis of GPX4 and SLC7A11 in cerebral cortex of *WT* and *Cpt1a*$^{As+/-}$ mice. Quantification expressed as a percentage of the *WT* group. Mean ± SD, n = 3 independent biological replicates. (**d**) The

levels of MDA and 4-HNE in cerebral cortex of *WT* and *Cpt1a*$^{As+/-}$ mice. Mean ± SD, n = 3 independent biological replicates. (**e**) The levels of Fe^{2+}, MDA, 4-HNE, ROS, T-GSH, GSSG, GSH, and GSH/GSSG in the peri-infarct area of *WT* and *Cpt1a*$^{As+/-}$ mice. Mean ± SD, n = 3 independent biological replicates. (**f, g**) Immunostaining showed the protein level of GPX4 (**f**) or SLC7A11 (**g**) in astrocytes. GFAP: red, GPX4 or SLC7A11: green and Hoechst: blue. White dotted lines, IBZ. The fluorescence intensity of GPX4 or SLC7A11 was analyzed, and the Mander's overlap coefficient was used to quantify protein colocalization. Scale bars, 50 μm or 10 μm. Mean ± SD, n = 3 independent biological replicates. One-way ANOVA followed by a post hoc Tukey's test (**a**, **e-g**). Student's *t* test (**b**, **c**). * $P < 0.05$， ** $P < 0.01$, *** $P < 0.001$, **** $P < 0.0001$.

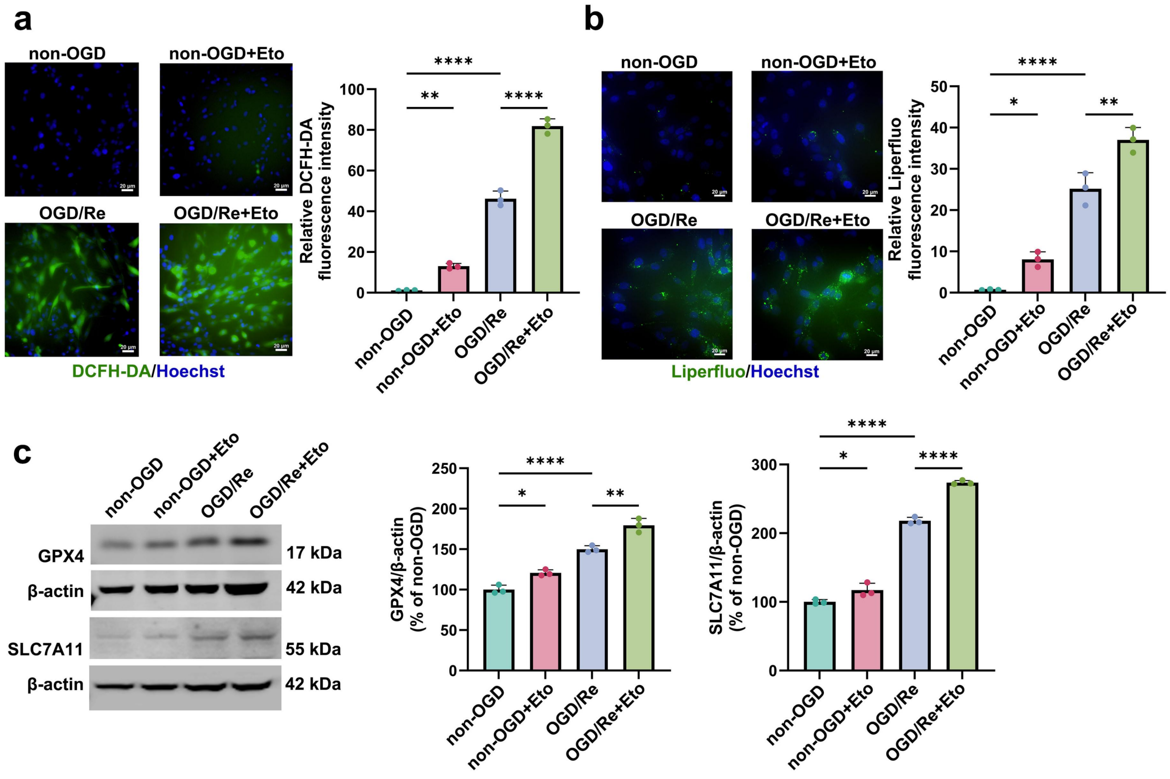

Figure S13. Pharmacological CPT1A inhibition by Eto promotes the increase of pro-ferroptosis markers and anti-ferroptosis protein in HA cells. HA cells were cultured with 10 μM Eto during reoxygenation for 24 h. (**a, b**) The levels of ROS and lipid peroxidation in Eto-treated HA cells. Scale bars, 20 μm. The fluorescence intensity of DCFH-DA or Liperfluo was analyzed, respectively. Mean ± SD, n = 3 independent biological replicates. (**c**) Western blotting analysis of GPX4 and SLC7A11 in Eto-treated HA cells. Quantification expressed as a percentage of the non-OGD group. Mean ± SD, n = 3 independent biological replicates. One-way ANOVA followed by a post hoc Tukey's test. * $P < 0.05$, ** $P < 0.01$, **** $P < 0.0001$.

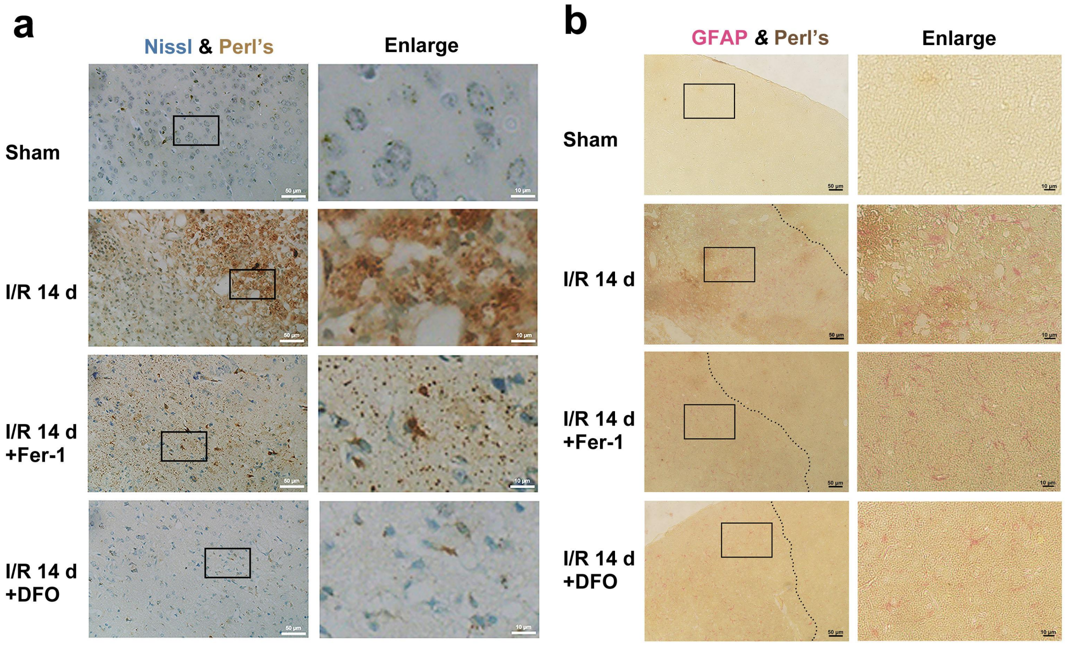

Figure S14. Antioxidant or iron chelator reduces astrocytic iron overload. (**a, b**) Fer-1 (1 mg/kg) or DFO (50 mg/kg) were intravenously injected (i.v.) at d 1 post-I/R, once every two days until to d 14 post-I/R. (**a**) Nissl & Perl's staining was used to detect the neuronal iron deposition. Iron deposition was visualized as yellow, brown, or even black granular deposits. Scale bars, 50 µm or 10 µm. n = 3 independent biological replicates. (**b**) GFAP & Perl's staining was used to detect iron deposition in astrocytes in the peri-infarct area. Alkaline phosphatase staining was used to visualize GFAP (astrocytes), and iron deposition was visualized as yellow, brown, or even black granular deposits. Scale bars, 50 µm or 10 µm. n = 3 independent biological replicates.

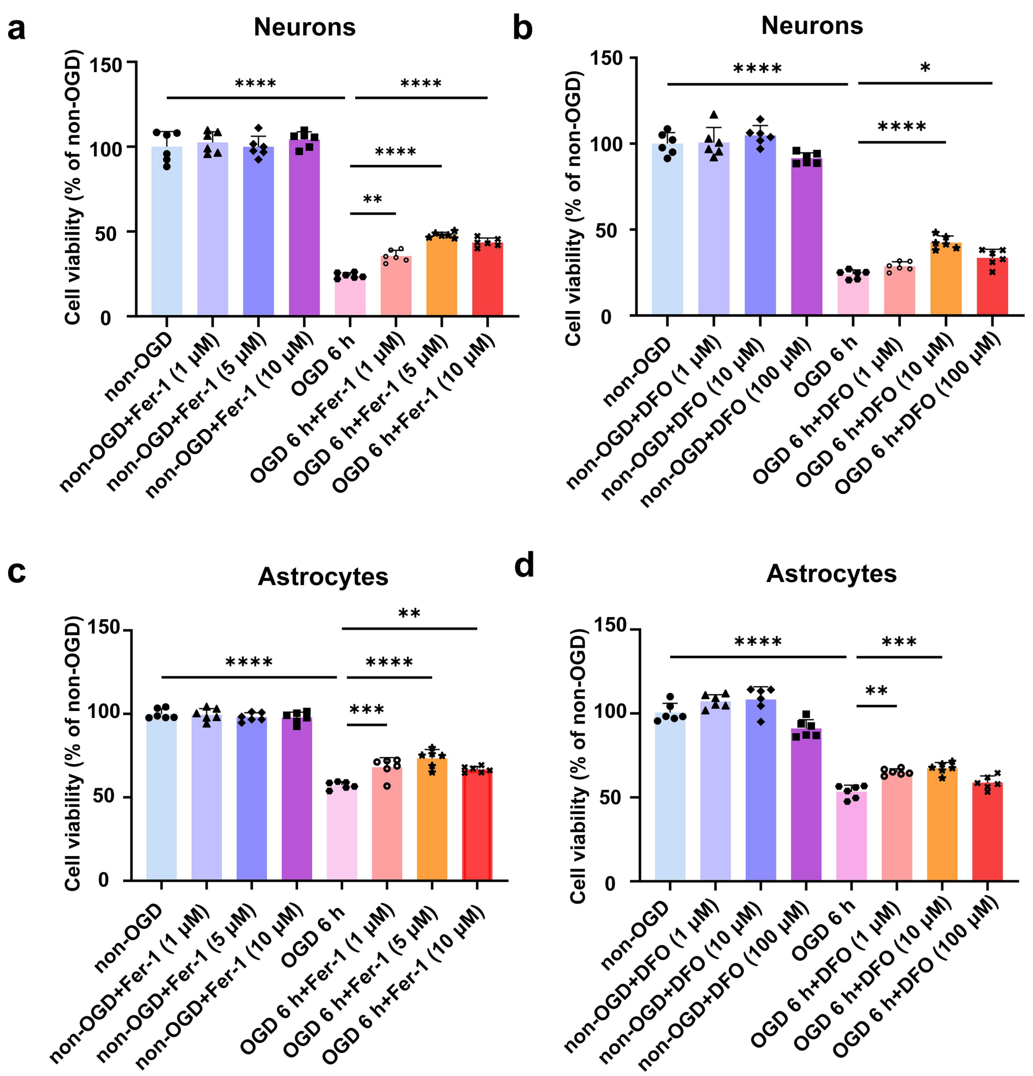

Figure S15. Antioxidant Fer-1 and iron chelator DFO reduce OGD 6 h-induced HT22 and HA cells injury. (a-b) HT22 cells were cultured with different concentrations of Fer-1 or DFO during OGD 6 h, and the cell viability was measured with CCK-8 assay. Mean ± SD, n = 6 independent biological replicates. **(c-d**) HA cells were cultured with different concentrations of Fer-1 or DFO during OGD 6 h, and the cell viability was measured with CCK-8 assay. Mean ± SD, n = 6 independent biological replicates. One-way ANOVA followed by a post hoc Tukey's test. * $P < 0.05$, ** $P < 0.01$, *** $P < 0.001$, **** $P < 0.0001$.

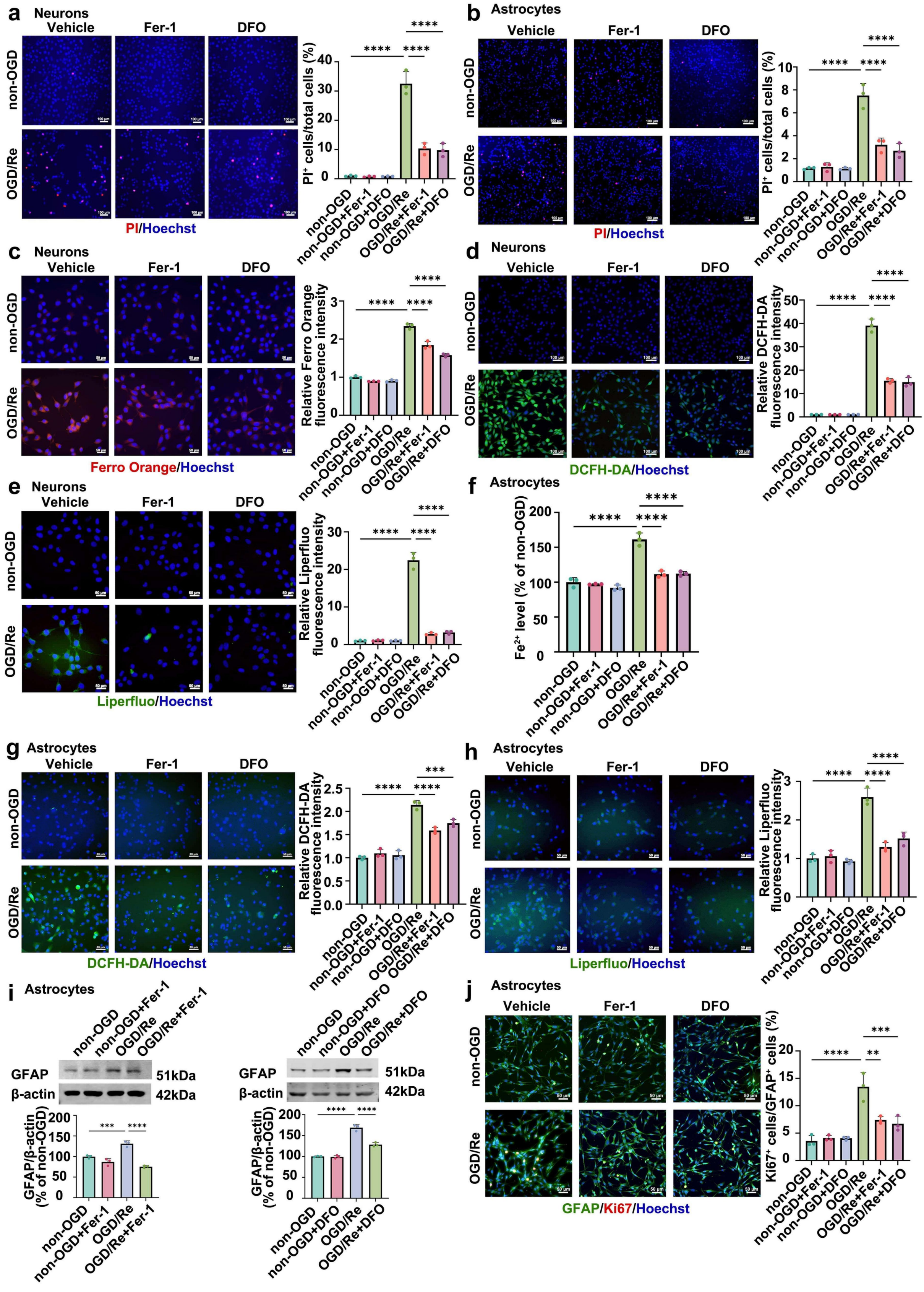

Figure S16. Fer-1 and DFO reduce neuronal cell death and astrocytic astrogliosis. HT22 cells and HA cells were cultured with 5 µM Fer-1 or 10 µM DFO during reoxygenation for 24 h. (**a, b**) PI staining showed the effects of Fer-1 and DFO on OGD/Re-induced HT22 cell (**a**) or HA cells (**b**) death. The ratio of PI^+ cells/total cells was analyzed. Scale bar: 100 µm. Mean ± SD, n = 3 independent biological replicates. (**c-e**) The levels of Fe^{2+} (**c**), ROS (**d**) or lipid

peroxidation (**e**) levels in OGD/Re-induced HT22 cells with Fer-1 or DFO treatment were detected by Ferro Orange, DCFH-DA and Liperfluo, respectively. The fluorescence intensity of Ferro Orange, DCFH-DA or Liperfluo were analyzed, respectively. Scale bar: 100 μm or 50 μm. Mean ± SD, n = 3 independent biological replicates. (**f-h**) The levels of Fe^{2+} (**f**), ROS (**g**) and lipid peroxidation (**h**) levels in OGD/Re-induced HA cells with Fer-1 or DFO treatment. The fluorescence intensity of DCFH-DA or Liperfluo was analyzed, respectively. Scale bar: 100 μm or 50 μm. Mean ± SD, n = 3 independent biological replicates. (**i**) Western blotting analysis of GFAP in OGD/Re-induced HA cells with Fer-1 or DFO treatment. Quantification expressed as a percentage of the non-OGD group. Mean ± SD, n = 3 independent biological replicates. (**j**) The effects of Fer-1 and DFO on astrogliosis in HA cells. GFAP: green, Ki67: red and Hoechst: blue. Scale bar, 50 μm. The ratio of $Ki67^{+}$ cells/total cells was analyzed. Mean ± SD, n = 3 independent biological replicates. One-way ANOVA followed by a post hoc Tukey's test. ** $P < 0.01$, *** $P < 0.001$, **** $P < 0.0001$.

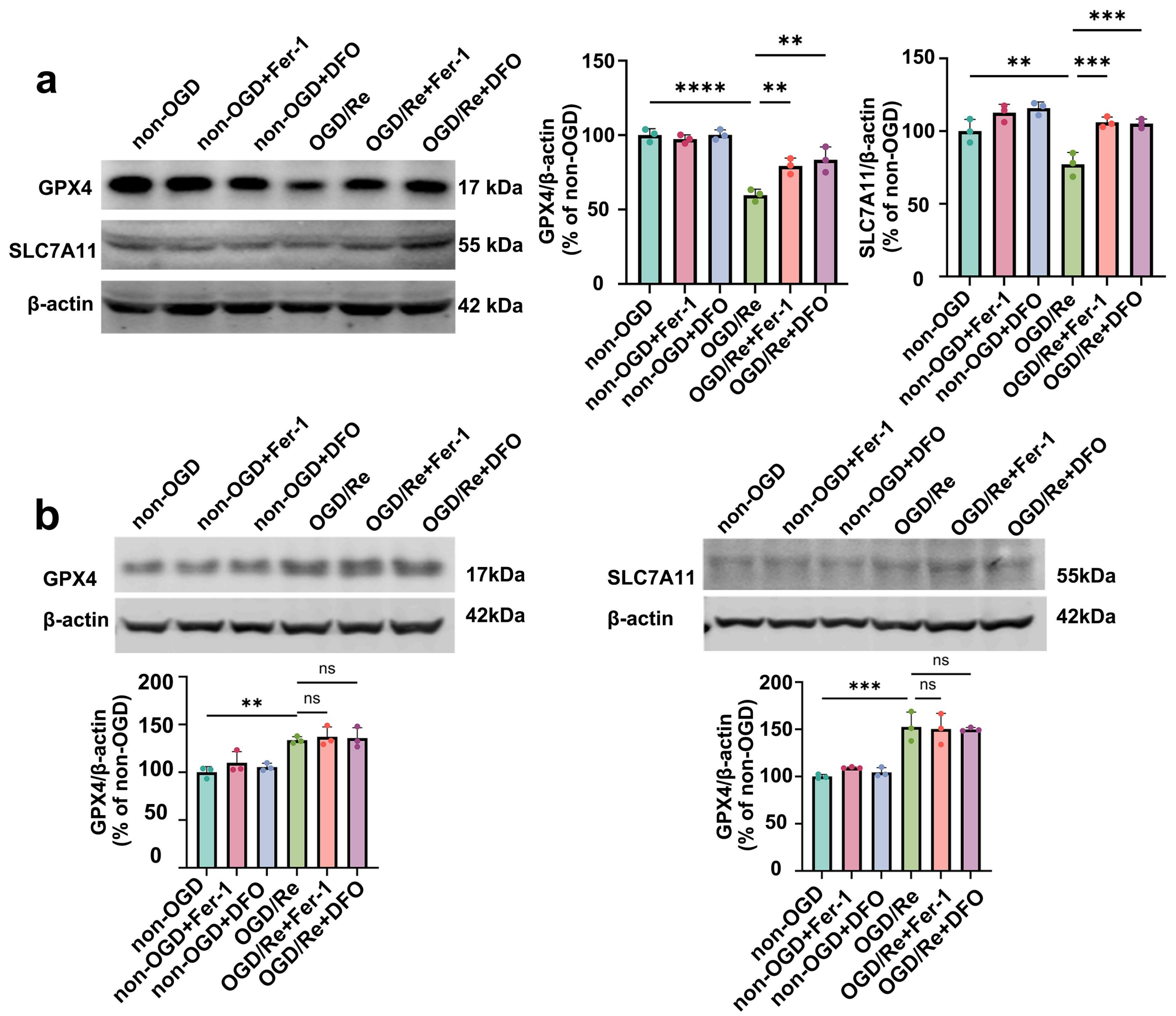

Figure S17. The levels of anti-ferroptosis protein in OGD/Re-treated HT22 cells or HA cells. HT22 cells or HA cells were cultured with 5 μM Fer-1 or 10 μM DFO during reoxygenation for 24 h. (**a**) Western blotting analysis of GPX4 and SLC7A11 in Fer-1 or DFO-treated HT22 cells. Quantification expressed as a percentage of the non-OGD group. Mean ± SD, n = 3 independent biological replicates. (**b**) Western blotting analysis of GFAP, GPX4 and SLC7A11 in Fer-1 or DFO-treated HA cells. Quantification expressed as a percentage of the non-OGD group. Mean ± SD, n = 3 independent biological replicates. One-way ANOVA followed by a post hoc Tukey's test. ** $P < 0.01$, *** $P < 0.001$, **** $P < 0.0001$.

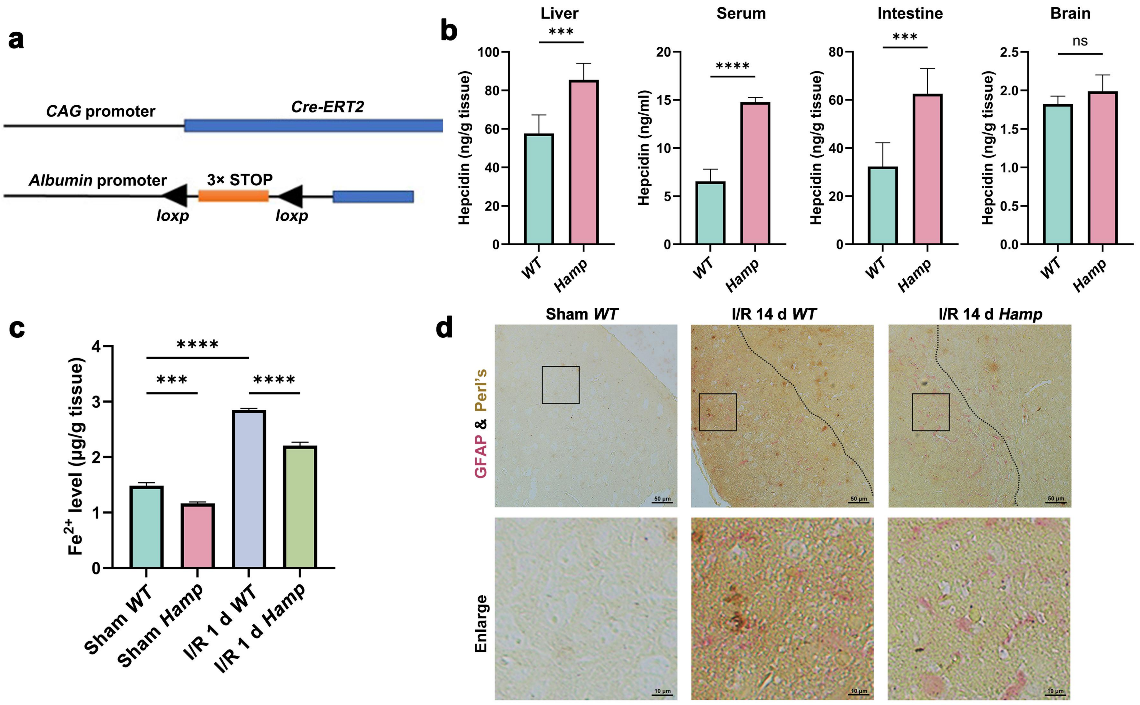

Figure S18. The Fe^{2+} level and iron deposition in cerebral cortex is reduced in *Hamp* mice with I/R compared with *WT* mice. Tamoxifen (0.1 mL per mouse, 10 mg/mL dissolved in sunflower oil) were peritoneally injected (i.p.) for consecutive 5 days to induce the overexpression of *Hamp* in liver. (**a**) Schematic of transgenic strategy of *Hamp* mice. (**b**) The level of hepcidin in the liver, small intestine, serum or brain tissue in *WT* and *Hamp* mice was detected by ELISA assay, respectively. Mean ± SD, n = 3 independent biological replicates. (**c**) The levels of Fe^{2+} in the peri-infarct area of *WT* and *Hamp* mice after I/R 1 day. Mean ± SD, n = 3 independent biological replicates. (**d**) GFAP & Perl's staining was used to detect iron deposition in astrocytes in the peri-infarct area of *WT* and *Hamp* mice. Alkaline phosphatase staining was used to visualize GFAP (astrocytes), and iron deposition was visualized as yellow, brown, or even black granular deposits. Scale bars, 50 μm or 10 μm. n = 3 independent biological replicates. Student's t test (**b**). One-way ANOVA followed by a post hoc Tukey's test (**c**). *** $P < 0.001$, **** $P < 0.0001$.

Supplementary Tables

Table S1. RT-qPCR primers for detecting genetic mouse

**Primer**	**Sequences**
Cpt1a-FWD	GCAAGGGTAGTTTGGGAGC
Cpt1a-REV	TCAGGCAGCAGGGAGAAG
GFAP-cre FWD	ACTCCTTCATAAAGCCCT
GFAP-cre REV	ATCACTCGTTGCATCGACCG
Tfrc-FWD	AATAGGGTAGCTTTGGCTGTTTTG
Tfrc-REV	TCTGGAGAAGCAAGATCAAACACT
hepcidin-ERT-FWD	GTGCCTGGCTAGAGATCCTG
hepcidin-ERT-REV	GATGTGGGAGAGGATGAGGA
hepcidin-Alb-FWD	ACCACTTGCCACCTATCACC
hepcidin-Alb-REV	GGAAAGTCCTTGGGGTCTTC

Table S2. The primary antibodies used in this study

Protein	Usage	Antibody
GFAP	IHC (1:1000), IF (1:1000)	ab4676, Abcam, UK
GFAP	WB (1:1000)	GTX108711, GeneTex, USA
NeuN	IHC (1:500)	ab177487, Abcam, UK
NeuN	IHC (1:1000), IF (1:1000)	ab134014, Abcam, UK
Ki67	IHC (1:200), IF (1:200)	ab16667, Abcam, UK
MAP2	IF (1:200)	GTX82661, GeneTex, USA
Na^+-K^+-ATPase	IF (1:200)	T55159M, Abmart, China
GPX4	IHC (1:200), WB (1:1000),	Ab125066, Abcam, UK
SLC7A11	IHC (1:200), WB (1:1000)	PA1-16893, Invitrogen, USA
SLC7A11	WB (1:1000)	T57046S, Abmart, China
Nrf-2	IHC (1:200), WB (1:1000)	GTX103322, GeneTex, USA
Nrf-2	WB (1:1000)	T55136S, Abmart, China
TfR1	WB (1:1000)	ab214039, Abcam, UK
TfR1	IP (1:100), WB (1:200), IHC (1:100)	sc-65882, Santa, USA
CPT1A	WB (1:1000), IHC (1:500)	ab128568, Abcam, UK
clathrin	IF (1:200)	4796S, CST, USA
Flag	WB (1:1000)	F1804, Sigma, USA
β-actin	WB (1:5000)	A5441, Sigma, USA

Abbreviations: WB, Western blotting; IF, Immunofluorescence; IHC, Immunohistochemistry

Table S3. The secondary antibodies used in this study

Protein	Usage	Antibody
Alexa Fluor® 594 goat anti-rabbit IgG (H+L)	IF (1:500), IHC (1:500)	A11012, Thermo, USA
Alexa Fluor® 594 goat anti-mouse IgG (H+L)	IF (1:500), IHC (1:500)	A11005, Thermo, USA
Alexa Fluor® 488 goat anti-rabbit IgG (H+L)	IF (1:500), IHC (1:500)	A11008, Thermo, USA
Alexa Fluor® 488 goat anti-mouse IgG (H+L)	IF (1:500), IHC (1:500)	A11001, Thermo, USA
Alexa Fluor® 647 goat anti-chicken IgG (H+L)	IHC (1:500)	ab150171, Abcam, UK
Alexa Fluor® 633 goat anti-rabbit IgG (H+L)	IHC (1:500)	A21070, Thermo, USA
Anti-Mouse lgG(H+L), Antibody, Dy Light M 680-Labeled	WB (1:10000)	042-06-18-06, KPL, USA
Anti-Rabbit IgG(H+L) Antibody, Dy Light M 800-Labeled	WB (1:10000)	042-06-15-06, KPL, USA

Abbreviations: WB, Western blotting; IF, Immunofluorescence, IHC, Immunohistochemistry

Table S4. The primer sequences for qPCR in this study

Sequences	Species
GPX4-FWD: ACAAGAACGGCTGCGTGGTGAA	human
GPX4-REV: GCCACACACTTGTGGAGCTAGA	human
SLC7A11-FWD: TCCTGCTTTGGCTCCATGAACG	human
SLC7A11-REV: AGAGGAGTGTGCTTGCGGACAT	human
GPX4-FWD: CCTCTGCTGCAAGAGCCTCCC	mouse
GPX4-REV: CTTATCCAGGCAGACCATGTGC	mouse
SLC7A11-FWD: CTTTGTTGCCCTCTCCTGCTTC	mouse
SLC7A11-REV: CAGAGGAGTGTGCTTGTGGACA	mouse
